# Patterns of Genomic Variations in the Plant Pathogen *Dickeya solani*

**DOI:** 10.3390/microorganisms10112254

**Published:** 2022-11-14

**Authors:** Slimane Khayi, Kok-Gan Chan, Denis Faure

**Affiliations:** 1 Biotechnology Research Unit, Regional Center of Agricultural Research of Rabat, National Institute of Agricultural Research (INRA), Avenue Ennasr, BP 415 Rabat Principale, Rabat 10090, Morocco; 2 Division of Genetics and Molecular Biology, Institute of Biological Sciences, Faculty of Science, University of Malaya, Kuala Lumpur 50603, Malaysia; 3 International Genome Centre, Jiangsu University, Zhenjiang 212100, China; 4 University of Paris-Saclay, CEA, CNRS, Institute for Integrative Biology of the Cell (I2BC), 91190 Gif-sur-Yvette, France

**Keywords:** soft rot, population genomics, horizontal gene transfer, phylogenomics

## Abstract

The plant pathogen *Dickeya solani* causes soft rot and blackleg diseases in several crops including *Solanum tuberosum*. Unveiling the patterns of its diversity contributes to understanding the emergence and virulence of this pathogen in potato agro-systems. In this study, we analyzed the genome of several *D. solani* strains exhibiting an atypically high number of genetic variations. Variant calling and phylogenomics support the evidence that the strains RNS10-105-1A, A623S-20A-17 and RNS05.1.2A belong to a divergent sub-group of *D. solani* for which we proposed RNS05.1.2A as a reference strain. In addition, we showed that the variations (1253 to 1278 snp/indels) in strains RNS13-30-1A, RNS13-31-1A and RNS13-48-1A were caused by a horizontal gene transfer event from a donor belonging to the *D. solani* RNS05.1.2A subgroup. The overall results highlight the patterns driving the diversification in *D. solani* species. This work contributes to understanding patterns and causes of diversity in the emerging pathogen *D. solani.*

## 1. Introduction

*Dickeya solani* is a plant pathogen causing soft rot and blackleg diseases in several plants, including potato and bulb plants [1]. This pathogen emerged in potato tuber cultivation (*Solanum tuberosum*) in Europe at the beginning of the 2000s [2]. Like the other necrotrophic pathogens of *Dickeya* and *Pectobacterium* genera, *D. solani* expresses a wide repertoire of plant-cell-wall-macerating enzymes and proliferates in plant lesions by exploiting cell remains [3]. 

A population genomics approach (76 genomes) pinpointed a low number of variations, such as single nucleotide polymorphisms (SNPs) and short (<10 bp) insertions and deletions (Indels), in a majority of the *D. solani* isolates collected in Europe and the Mediterranean Basin over the two last decades [4]. This low diversity is consistent with a bottleneck associated with a recent spread from a small-size inoculum. The primary hosts and environmental reservoirs, where a wider diversity of *D. solani* is expected, are still unknown. 

One of the clues to understanding natural diversity in *D. solani* is the isolate RNS05.1.2A, collected in a potato plant exhibiting lesions. More than 30,000 SNPs and Indels are scattered in its genome as compared to archetypical isolates that are the strain type IPO2222 and other extensively studied strains Ds0432.1 and RNS08.23.3.1A (=PRI3337) [5]. The strain RNS05.1.2A clearly emerged with a long branch in distance trees based on gene or protein sequences of *D. solani* isolates [4]. Other clues are events of horizontal gene transfers (HGT), either based on insertion and recombination of mobile elements (mainly phages), or gene replacing by homologous recombination [6]. Phages contribute to an important part of the accessory pangenome of *D. solani*. Gene-replacing events are less frequently observed [5], but they highlight the capacity of *D. solani* to recombine with DNA sequences from genetically related bacteria. For instance, *D. solani* strains PPO9019 and PPO9134 contain distinctive replacing events with sequences from *D. dianthicola*, while strain RNS07.7.3B contains several replacing events with sequences from a bacterium that is phylogenetically close to the isolate RNS05.1.2A [5].

In a previous study, we compared genetic variations (SNPs and Indels) of 76 *D. solani* genomes, including those available in databases [4]. Among them, ten isolates showed a high number of SNPs/Indels: RNS05.1.2A which is presently the most divergent isolate in *D. solani*, PPO9134 and PPO9019, which exhibit replacing events from *D. dianthicola*, RNS07.7.3B with replacing events from an unknown RNS05.1.2A-related bacterium, and RNS10-105-1A, RNS13-30-1A, RNS13-31-1A, RNS13-48-1A and RNS15-102-1A in which variations are still uncharacterized. In this work, we combined PacBio and Illumina approaches to obtain a complete genome sequence of the strain RNS05.1.2A and then we analyzed causes of variations in RNS10-105-1A, RNS13-30-1A, RNS13-31-1A, RNS13-48-1A and RNS15-102-1A. This work contributes to understanding patterns of diversity in the emerging pathogen *D. solani*. 

## 2. Materials and Methods

### 2.1. Bacterial Strains

The *D. solani* strains used in this study were collected from symptomatic plant tissues using a selective crystal violet pectate medium [7] from a different date in France (Table S1). The strains were routinely cultured in a TY medium (tryptone 5 g/L, yeast extract 3 g/L and agar 1.5%) at 28 °C.

### 2.2. Genome DNA Extraction, Sequencing and Assembly

The total DNA was extracted from the *D. solani* strains RNS10-105-1A, RNS13-30-1B, RNS13-31-1A, RNS13-48-1A, RNS15-102-1A and RNS05.1.2A using MasterPure Complete DNA and RNA Purification Kit (Epicentre, Illumina). The quantity and quality controls of extracted DNA were performed using a NanoDrop (ND 1000) device and agarose gel electrophoresis at 1.0% (*w/v*), respectively. 

Paired-end libraries were constructed for RNS10-105-1A, RNS13-30-1B, RNS13-31-1A, RNS13-48-1A and RNS15-102-1A and the sequencing was performed on an Illumina NextSeq500 instrument using a High Output Sequencing Kit with 75 × 2 cycles at I2BC-platform (Gif-sur-Yvette, France). For each strain, about 6.9–18 million reads were obtained, corresponding to an average coverage ranging from 100× to 270×. The reads were trimmed (quality score threshold 0.05) and assembled using CLC genomics workbench V12 (Qiagen, Denmark). The annotation of the strains was performed using Prokka software V1.14.6 [8] with the following parameters (—addgenes —genus Dickeya —species solani —kingdom Bacteria —gcode 11 —usegenus).

In the case of *D. solani* RNS05.1.2A, a draft genome (37 contigs, JWMJ00000000.1) was previously generated from Illumina HiSeq 2000 version 3, sequencing paired-pair libraries [5]. To achieve the complete assembly of the genome, the genomic DNA was sequenced using the PacBio RS II sequencing platform (Pacific Biosciences, Menlo Park, CA, USA) and Illumina reads were used to correct the assembled PacBio genome.

The phages content analysis was performed using the platform PHASTER [9,10].

### 2.3. Phylogenomics

To highlight phylogenetic relationships between the *D. solani* isolates, we used a core-proteome phylogenomics approach. In addition to the predicted proteomes of RNS10-105-1A, RNS13-30-1B, RNS13-31-1A, RNS13-48-1A, RNS15-102-1A and RNS05.1.2A, we retrieved those of 34 *D. solani* (Table S1) available at GenBank (https://www.ncbi.nlm.nih.gov/genome/, accessed on 13 January 2022). The 40 proteomes were clustered using OrthoFinder software version 2.4.0 [11] with the following command: orthofinder -t 56 -M msa -A mafft -T iqtree -f orthofineder/. The phylogenetic tree was inferred from concatenated core-proteomes using IQTREE version 2.1.4-beta [12] with the following command: iqtree -s SpeciesTreeAlignement.phylip -nt AUTO -ntmax 50 -seed 12345 -bb 1000. The species *Dickeya dadantii* was used as an outgroup.

The calculations of the pairwise ANI (Average Nucleotide Identity) values were performed using FastANI software [13] and the tree plot was performed with ggtree [14]. All the commands are available through the GitHub repository (https://github.com/SolayMane/Dsolani_HGT_paper.md/, accessed on 13 January 2022).

### 2.4. Variant Calling and Analysis

We conducted a variant calling analysis of *D. solani* strains RNS10-105-1A, RNS13-30-1B, RNS13-31-1A, RNS13-48-1A and RNS15-102-1A, as well as seven other *D. solani* strains: IPO2222^T^, Ds0432.1, RNS07.7.3B, RNS05.1.2A, A623S-20A-17, PPO9019 and PPO9134. These strains were included in this study because they exhibited a variable degree of divergence according to phylogenetic analysis. The variant calling was performed using a Snippy pipeline (version 4.6.0) [15] and a complete genome of the strain RNS08.23.3.1A (NZ_CP016928.1/CP016928.1) as a reference sequence. The variants’ densities were calculated and plotted using a karyoploteR package [16]. 

### 2.5. Phylogenetic Analysis and Annotation of Variants’ Hotspot Regions within D. solani Strains

To analyze the phylogenetic relationship of the variants’ hotspot within *D. solani* strains, we extracted the 1 kbp window sequences harboring those variants based on a cut-off of variant density above 9 variants/1kbp. To do so, we retrieved the coordinates of the 1 kbp windows based on the predefined cut-off, then using these coordinates to obtain the corresponding sequences for each species from the whole genome SNP alignment including invariant sites generated by Snippy. In this analysis, we included all the 40 *D. solani* strains used in phylogeny and the *D. dadantii* 3937 sequence as an outgroup. The workflow and commands are available at: https://github.com/SolayMane/Dsolani_HGT_paper.md/blob/main/hgt_regions.md, accessed on 13 January 2022

For each alignment extracted, corresponding to the 45 regions that were identified, a phylogenetic tree was inferred using FastTree software version 2.1.11 [17] with the following parameters: -nt -boot 1000 -gtr.

Furthermore, to investigate the functional characteristics of the genes with variations in the four strains RNS13-48-1A, RNS13-30-1B, RNS15-102-1A and RNS13-31-1A, we extracted the genes of the 45 identified regions. Functional annotation of the 63 unique genes was performed using emapper-2.1 [18] based on EggNOG orthology data [19]. Sequence searches were performed using Diamond software [20].

## 3. Results

### 3.1. Complete Genome Sequence of Atypical Strain D. solani RNS05.1.2A

*D. solani* RNS05.1.2A exhibits a high number of variations as compared to *D. solani* RNS08.23.3.1A [5]. To unveil the genomic characteristics behind this variability, we obtained a complete genome sequence of the strain RNS05.1.2A through PacBio sequencing followed by a PacBio-homopolymer error correction step using Illumina shotgun sequencing data. The complete genome sequence of *D. solani* RNS05.1.2A consists of a single circular chromosome totaling 5,069,883 bp with a GC content of 56%. The RAST annotation process generated a total of 4833 predicted genes including 4736 Coding protein sequence, 75 tRNA and 22 rRNA organized in seven operons.

**Figure 1:**
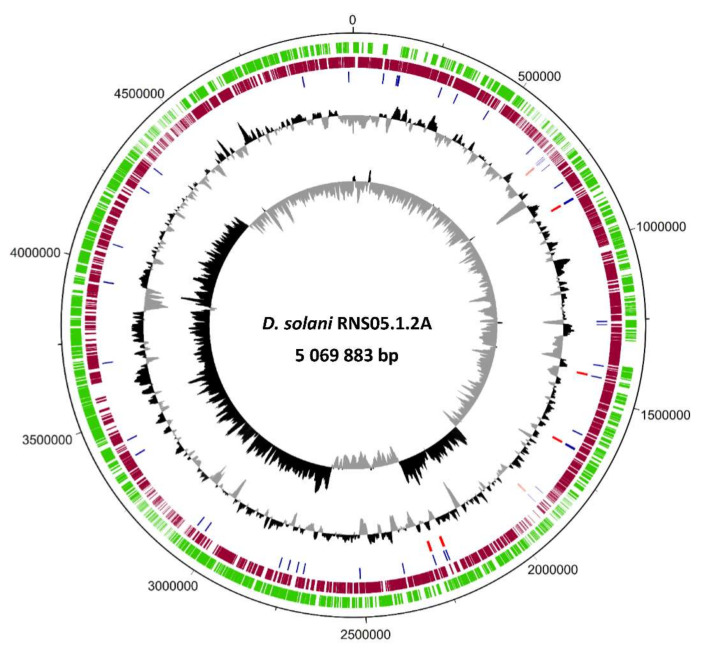
A circular map of *D. solani* RNS05.1.2A chromosome sequence. The genome map was generated using DNAPlotter. The two inner circles show the GS-skew plotter, followed by GC content. The red bars indicate positions of the rRNAs’ genes and the blue ones indicate the tRNAs’. The coding sequences are indicated by bars in green (forward direction) and dark red (reverse direction).

Figure 1 represents a circular map of the complete genome with annotations. Interestingly, we identified a fragment of 347,817 bp inverted within the *D. solani* RNS05.1.2A chromosome. Using Illumina reads, we verified the correct mapping at the junction of this inversion. The flanking region 5′ of this fragment harbors a gene coding for a phage tail length tape measure protein (GpT), which is known to dictate the tail length and facilitate DNA transit to the cell cytoplasm during infection. The flanking region 3′ encodes a phage integrase that is known to mediate unidirectional site-specific recombination between the phage attachment site and the bacterial attachment site [21]. The genome sequence was deposited at NCBI with the accession number CP104920.

**Figure 2:**
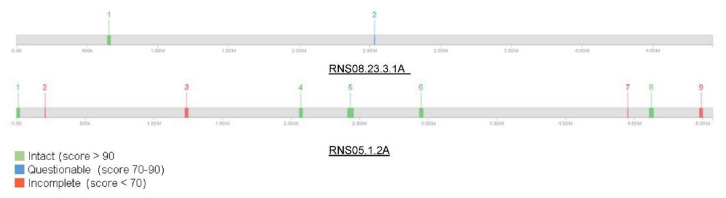
Graphical representation of phage distribution in *D. solani* strains RNS05.1.2A and RNS 08.23.3.1A genome sequences. The numbers 1 to 9 represent the detected phages content.

We analyzed further phages content of *D. solani* RNS05.1.2A and RNS08.23.3.1A using PHASTER software. The analysis showed that the *D. solani* RNS05.1.2A genome is richer in phages elements as compared to RNS08.23.3.1A (Figure 2). Strain RNS05.1.2A harbors nine regions including five intact and four questionable prophage regions totaling 250 kbp. In contrast, the strain RNS08.23.3.1A contained only two prophage regions, one intact (27.4 Kbp) and the other questionable (9.2 kbp) (Table S4). 

### 3.2. Phylogenomics of D. solani Strains RNS10-105-1A, RNS13-30-1B, RNS13-31-1A, RNS13-48-1A and RNS15-102-1A

The draft genome sequences of *D. solani* strains RNS10-105-1A, RNS13-30-1B, RNS13-31-1A, RNS13-48-1A and RNS15-102-1A were assembled using CLC Genomics V12 and then annotated using Prokka. The assembly and annotation statistics are provided in Table S2. In addition to these five strains, we retrieved 35 *Dickeya solani* genome sequences available at GenBank (https://www.ncbi.nlm.nih.gov/genome/, accessed on 13 January 2022) to perform core-genome based phylogenetic analysis. The predicted proteomes of the 40 strains were clustered using OrthoFinder software. The analysis showed that 99.6% of the genes are clustered into orthogroups with a total of 4600 genes that represent the pan-genome of *D. solani* species. The core-genome fraction included a total of 3457 genes representing 75% of the pan-genome, while the accessory genome contained 1143 genes (25%) (Table S3).

**Figure 3:**
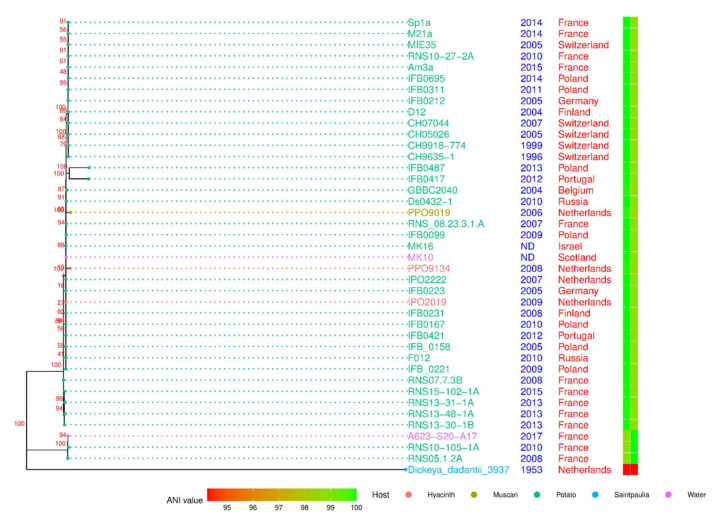
Core-genome maximum-likelihood phylogenetic tree of 40 *D. solani* strains. *D. dadantii* 3937 was used as an outgroup. The tree was inferred from an alignment of 3181 protein sequences for each strain. The date and geographical location of isolation of the *D. solani* strains are mentioned. The heatmap indicates ANI value calculated against the strains RNS08.23.3.1.A (**left**) and RNS05.1.2A (**right**).

The phylogenomic tree was constructed using the concatenated genes of the core genome (Figure 3) with *D. dadantii* as an outgroup. The general topology shows an overall genomic homogeneity of the *D. solani* population sampled to date. However, some long branches emerged, indicating a high number of variations in a few of the strains. Noticeably, the strains RNS10-105-1A and A623-S20-A17 were grouped together with RNS05.1.2A (100% bootstrap value) in a cluster that we called the RNS05.1.2A subgroup. Using RNS08.23.3.1A as a reference, the pairwise ANI values were equal to or above 99.9, but those of strains of the *D. solani* RNS05.1.2A subgroup were below 98.7% (Figure 3). Hence, phylogeny and ANI approaches support the existence of the RNS05.1.2A subgroup within the *D. solani* species [22]. As we observed in the case of the strain RNS05.1.2A, the strains RNS10-105-1A and A623-S20-A17 were rich in phage sequences (Table S4). 

In the rooted distance tree (Figure 3), five strains were also grouped (100% bootstrap value): RNS07.7.3B, RNS13-30-1B, RNS13-31-1A, RNS13-48-1A and RNS15-102-1A. Their phylogenetic position indicated that they could share some variations with strains of the RNS05.1.2A subgroup. Two other strains, PPO9019 and PPO9134, exhibit a long branch. A previous study revealed that they acquired genes from *D. dianthicola* by HGT [5]. The strains IFB0417 and IFB0487 also showed a long branch, but the sequencing approach (a PacBio technology with uncorrected homopolymer errors) could explain the observed variations. These two strains were not considered further in our study.

### 3.3. SNP and Indel Variations in D. solani Strains

To uncover the genetic variability within *D. solani* strains, the variants (Complex, Del, INS, MNP, SNP) against the *D. solani* RNS08.23.3.1A genome (CP016928.1) were identified using Snippy software (Table S5). We analyzed twelve strains: RNS10-105-1A, A623-S20-A17 and RNS05.1.2A (from the RNS05.1.2A subgroup), RNS07.7.3B, RNS13-30-1B, RNS13-31-1A, RNS13-48-1A and RNS15-102-1A (all five in the same phylogenetic cluster), strains PPO9019 and PPO9134 (with HGT from *D. dianthicola*) and IPO2222^T^ and Ds0432.1 exhibiting a low number of variations.

As compared to RNS08.23.3.1A, the number of variants ranged from 9 (Ds0432.1) to 39,743 (RNS05.1.2A). Table 1 summarizes the variant analysis. The RNS05.1.2A subgroup (RNS10-105-1A, A623-S20-A17 and RNS05.1.2A) exhibited the highest number of genes with variations (3854–3883), while only 8 to 10 genes with variants were in strains IPO2222 and Ds0432.1. On the other hand, a moderate number of genes with variations, ranging from 47 to 154 genes, was observed in the remaining strains: RNS07.7.3B, RNS13-30-1B, RNS13-31-1A, RNS13-48-1A and RNS15-102-1A, PPO9019 and PPO9134. 

**Figure 4:**
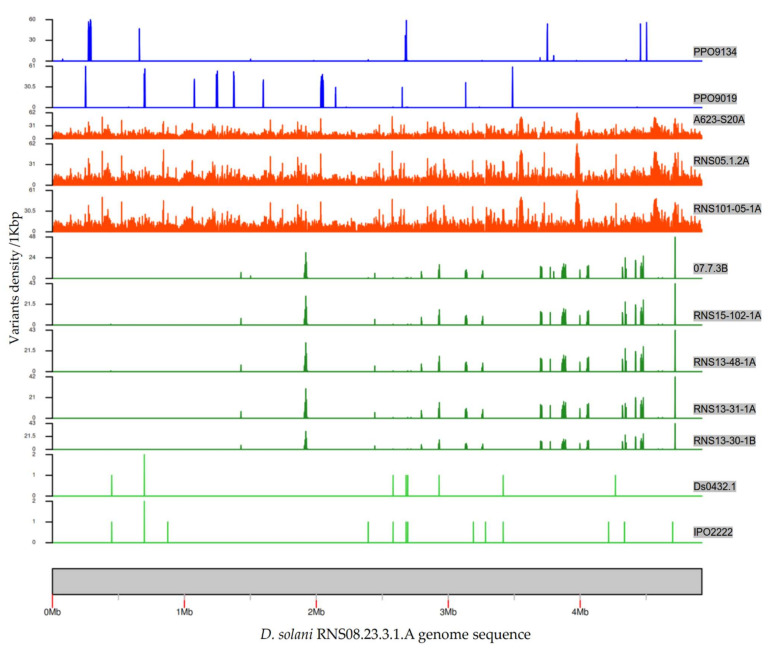
Graphical representation of the densities of variants in each *D. solani* strain. The *D. solani* RNS 08.23.3.1A genome was used as a reference. The densities were calculated based on a window size of 1 kbp. Scale of variants’ density/kbp was automatically adjusted, hence was not the same in each plot.

To represent the chromosomal distribution of the variants, we calculated and plotted the variants’ densities across the RNS08.23.3.1A genome sequence (Figure 4). In the five strains RNS07.1.2B, RNS13-30-1B, RNS13-31-1A, RNS13-48-1A and RNS15-102-1A, the variants were found to be clustered on identical coordinates across the reference genome sequence. In these five strains, the average of variations was 0.25 variants per kbp. In strains PPO9019 and PPO9134, variations were grouped in other regions that were shown to be acquired from *D. dianthicola* [3]. In strains RNS101.05.1A, RNS05.1.2A and A623-S20A, the variants were scattered along the chromosome sequence with an average of seven variants per kbp. This high variability is in accordance with the branch length of the RNS05.1.2A subgroup within the phylogenomic tree (Figure 3). 

### 3.4. Characteristics of HGT Events in D. solani Strains RNS13-30-1B, RNS13-31-1A, RNS13-48-1A and RNS15-102-1A

**Figure 5:**
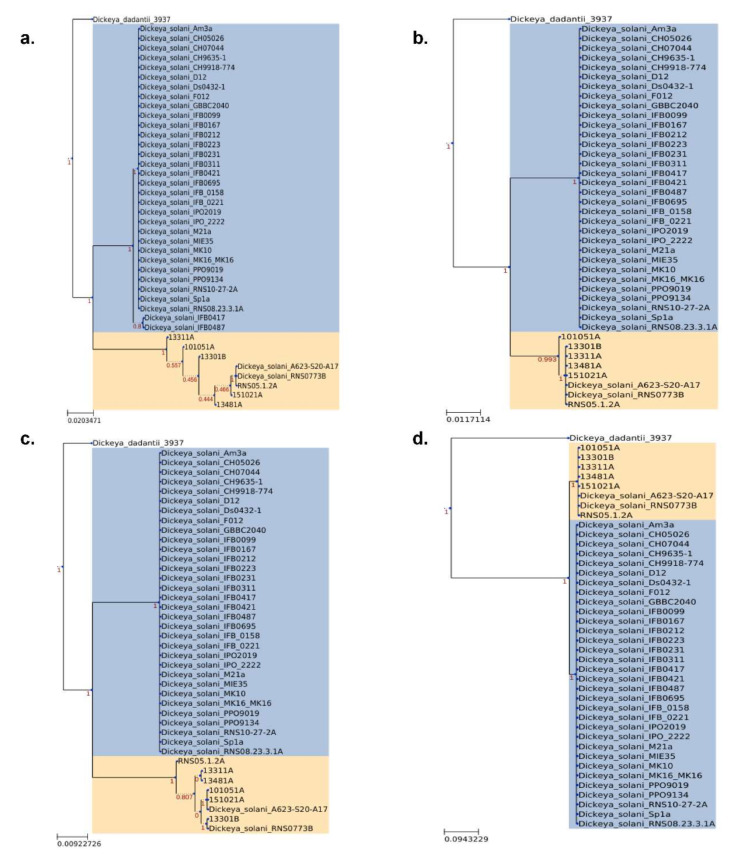
Phylogenetic analysis of four regions highlighting high variant densities. In (**a**–**d**) are, respectively, regions 1, 2, 3 and 4 with 42, 30, 28 and 21 variants/kbp. The bootstrap values are indicated in red. *D. dadantii* 3937 sequence was used as an outgroup.

We further analyzed the pattern of variations in the strains RNS13-30-1B, RNS13-31-1A, RNS13-48-1A and RNS15-102-1A and RNS07.7.3B. To do so, we extracted all the 1 kbp window sequences with a variant density >9 variants/1 kbp and performed phylogenetic analysis. In total, 45 regions were extracted and numbered from 1 to 45. Figure 5 presents a phylogenetic analysis of four of them: region 1 (42 variants/1 kbp), region 2 (30 variants/1 kbp), region 3 (28 variants/1 kbp) and region 4 (21 variants/1 kbp). The topology of the trees highlighted two distinct clades that are supported by high bootstrap value (>0.9): the first one (Figure 5, in beige) includes sequences of the strains RNS13-30-1B, RNS13-31-1A, RNS13-48-1A, RNS15-102-1A, RNS07.7.3B, RNS10-105-1A, A623S20A17 and RNS05.1.2A; the second one (Figure 5, in blue) includes those of the other *D. solani* strains. Phylogenies of the remaining 41 regions showed a similar pattern and are provided in the supplementary data (Figure S1). Hence, in RNS13-30-1B, RNS13-31-1A, RNS13-48-1A, RNS15-102-1A as well as RNS07.7.3B, our phylogenetic analysis revealed a plausible origin of the variants through horizontal gene transfer and replacing from strain(s) of the *D. solani* RNS05.1.2A subgroup. 

### 3.5. Functional Annotation of the Variable Regions in D. solain Strains

**Figure 6:**
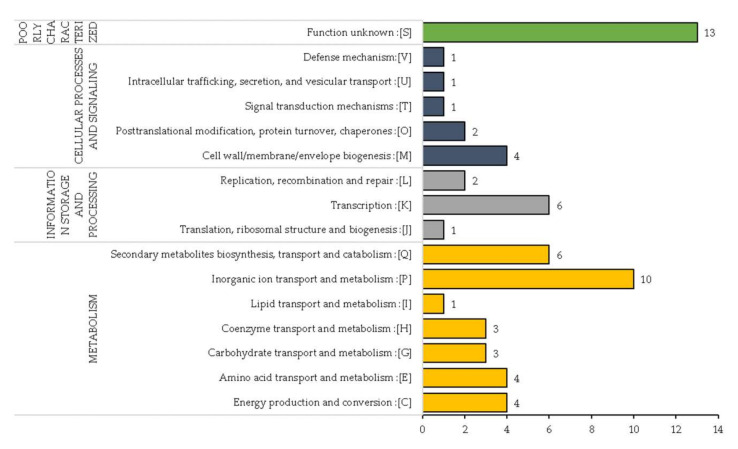
Classification of COG annotations of the genes into 4 general functional categories. The numbers close to the bars indicate the number of genes associated to each COG subcategory.

In strains RNS13-48-1A, RNS13-30-1B, RNS15-102-1A and RNS13-31-1A, we investigated gene function within the 45 identified regions with variations. In these four strains, 63 unique genes exhibited variations. The genes were scanned against the EggNOG database (v5.0) in order to categorize them according to their Gene Ontology (GO) category. Out of these 63 genes, the seed orthologs search resulted in 59 genes with significant hits that were used to annotate the genes (Table S6). In total, 62 COGs were assigned to 59 genes where the COG annotations were classified into four general functional categories including Metabolism, Poorly Characterized, Cellular Processes and Signaling and Information Storage and Processing, with 31 (50%), 13 (21%), 9 (15%) and 9 (14%) hits, respectively (Figure 6). The top three COG functional categories were 1—Unknown function, 2—Inorganic ion transport and metabolism and 3—Transcription with, respectively, 13, 10 and 6 genes.

In addition, among the 59 analyzed genes, 35 were also assigned to at least one KEGG Orthology identifier. These genes were used to query the KEGG ORTHOLOGY Database to highlight the pathways in which those genes are involved. The majority of the genes were involved in Metabolic Pathways (map01100), ABC transporters (map02010), Quorum Sensing (map02024), the Bacterial secretion system (map03070) and the Biosynthesis of secondary metabolites (map01110). The results are detailed in Tables S7 and S8.

**Figure 7:**
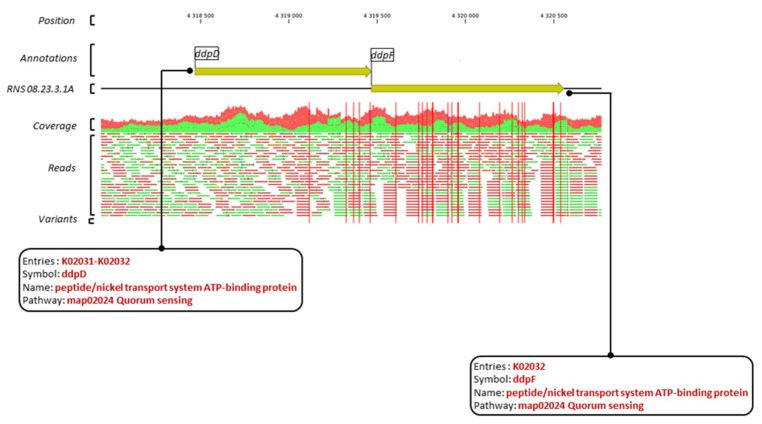
Graphical representation of variants within the *ddpD* and *ddpF* genes and their respective annotations. The red vertical bars indicate position of the variants. Horizontal bars in green and red represent the mapped forward and reverse reads against the reference sequence.

Figure 7 exemplifies region 26 in which the *ddpF* and *ddpD* genes, which display 5 and 16 variants, respectively, encode components of a putative peptide/nickel ABC-transport system.

## 4. Discussion

This work highlighted different patterns of variations in the emerging pathogen *D. solani*. 

*D. solani* isolates distribute into two different subgroups, diverging with more than 30,000 variations (SNPs and Indels) along the chromosome. In the first subgroup is the strain type IPO2222^T^, as well as most of the isolated strains such as Ds0432.1 and RNS08.23.3.1A, while in the second one are RNS101-05-1A, RNS05.1.2A and A623-S20-A17. We are proposing the strain RNS05.1.2A as a reference strain for this subgroup, as we obtained and deposited in GenBank its complete genome sequence (CP104920). The strains RNS08.23.3.1A and RNS05.1.2A reached the same level of aggressiveness in tuber maceration assay [5].

Aside SNP and Indels, we observed that integrated phage genes also contribute to the variability of *D. solani* strains of both RNS05.1.2A and IPO2222^T^ subgroups. Phage gene integration contributes to additive HGT events; hence, they also contribute to extending the pangenome repertoire [6,23]. Phages may also facilitate recombination, causing inversion or deletion. In the case of RNS05.1.2A, a large inverted region is flanked by phage genes that could have contributed to a recombination event. 

Another cause of variations in *D. solani* is replacing HGT events. These variations were observed in several strains belonging to the IPO2222^T^ subgroup. They are caused by replacing one or more genes by their orthologs from genomes of the *D. solani* RNS05.1.2A subgroup (this work) and *D. dianthicola* [5]. The precise process by which these events occur is not known. Remarkably, in the same genome, several regions were affected, suggesting an integration of several DNA fragments from a same origin. Phage integrases could be involved in the integration of multiple DNA fragments acquired by HGT. Phage-independent processes of HGT could also drive genomic variations such as transformations and cell fusion [24]. These replacing HGT events contribute to a local increase of variations (SNPs). In coding regions, these variations provoke synonymous or non-synonymous variations in proteins [5]. A wide panel of functions are affected by replacing HGT, including transport of metabolites and inorganic compounds. The core genes (PCWDEs and their regulons) known to be involved in the blackleg and soft rot symptoms [25] were not found to be concerned by these genomic variations in the five strains with the exception of the protease gene *prtA* (D083_3571) that shows 17 variants conferring seven amino acid changes (Table S7).

The strains RNS07.7.3B, RNS13-30-1B, RNS13-31-1A, RNS13-48-1A and RNS15-102-1A exhibited a remarkable pattern of replacing HGT events: all occurred at the same positions. This pattern may be caused by either independent HGT events at the same position in the genome of different individual cells, or by sampling a same clone several times (years 2007, 2013 and 2015). We believe the second hypothesis is the most probable. Hence, this pattern suggests that the HGT events occurred in a common ancestor of the studied strains. This would exemplify persistence of a same *D. solani* clone over years. The causes of this persistence are not known, nor is whether replacing HGT variations could contribute to it. Noticeably, the isolates belonging to the RNS05.1.2A subgroup were collected in France and the isolates, which acquired genes by HGT from the RNS05.1.2A subgroup, were also collected in France. It would be interesting to further analyze the distribution of this rare subgroup in other countries.

More generally, the emergence of *D. solani* suggests two successive steps: firstly, a drastic reduction of diversity at the transfer time onto the potato host, and then a potential diversification based on stochastic variations (SNPs, Indels, phages, replacing HGT). Whether some variations could contribute to the fitness and maintenance of the *D. solani* of the potato host remains an open question. 

## Figures and Tables

**Table 1:** Statistics of variants identified within 12 *D. solani* strains against the strain RNS08.23.3.1A.

Strain	Total	Genic	IG ^1^	NG ^2^	Syn ^3^	NSy ^4^	Cp ^5^	SNP ^6^	MNP ^7^	DEL ^8^	INS ^9^
IPO2222	14	11	3	10	6	3	0	10	0	3	1
Ds0432.1	9	9	0	8	6	1	0	7	0	1	1
RNS13-30-1B	1268	1148	120	151	877	263	133	1101	20	6	8
RNS13-31-1A	1253	1144	109	151	877	261	128	1090	23	5	7
RNS13-48-1A	1269	1150	119	152	879	263	131	1102	23	6	7
RNS15.102.1A	1273	1153	120	152	882	263	127	1105	27	6	8
RNS07.7.3B	1278	1157	121	154	879	265	156	1105	0	8	9
RNS10.105.1A	39,235	34,251	4984	3877	26,557	7465	4367	33,885	516	248	219
RNS05.1.2A	39,743	34,627	5116	3883	26,715	7648	4445	34,044	750	264	240
A623S20A17	38,623	33,803	4820	3854	26,126	7375	4868	33,163	1	301	290
PPO9019	2573	2374	199	68	1906	449	589	1961	0	14	9
PPO9134	1842	1751	91	47	1350	396	492	1341	0	5	4

^1^ Intergenic, ^2^ Number of genes, ^3^ Synonymous, ^4^ Non-synonymous, ^5^ Complex (combination of SNP/MNP), ^6^ Single Nucleotide Polymorphism, ^7^ Multiple Nucleotide Polymorphism, ^8^ Deletion, ^9^ Insertion.

## Data Availability

Sequence data were deposited at NCBI under accession number PRJNA882758.

## Supplementary Materials

The following are available online at https://www.mdpi.com/article/10.3390/microorganisms10112254/s1. Table S1: Metadata of *D. solani* strains used in this study; Table S2: Statistics of genome assemblies and their annotations; Table S3: Statistics of OrthoFinder analysis on 40 proteomes of *D. solani* strains; Table S4: Phages content analysis of *D. solani* strains; Table S5: Statistics of variant calling on 11 *D. solani* strains against the reference strain; Table S6: Functional annotation of 59 genes with EggNOG database; Table S7: Functional analysis of 35 genes on KEGG ORTHOLOGY Database; Table S8: Summary of the number of genes within pathways; Figure S1: Individual phylogenetic trees of 41 hotspot regions presented in order from region 5 to region 45. References [4,5,23,26,27,28,29,30,31,32] are cited in the supplementary materials.
