# Supplementary material for: Patterns of Genomic Variations in the Plant Pathogen Dickeya solani
Source: Microorganisms. 2022 Nov 14;10(11):2254. doi: 10.3390/microorganisms10112254 (PMC9699125; doi:10.3390/microorganisms10112254)
Supplement: Supplementary file 1 [file microorganisms-10-02254-s001.zip › microorganisms-1997831-Figure S1.pdf]

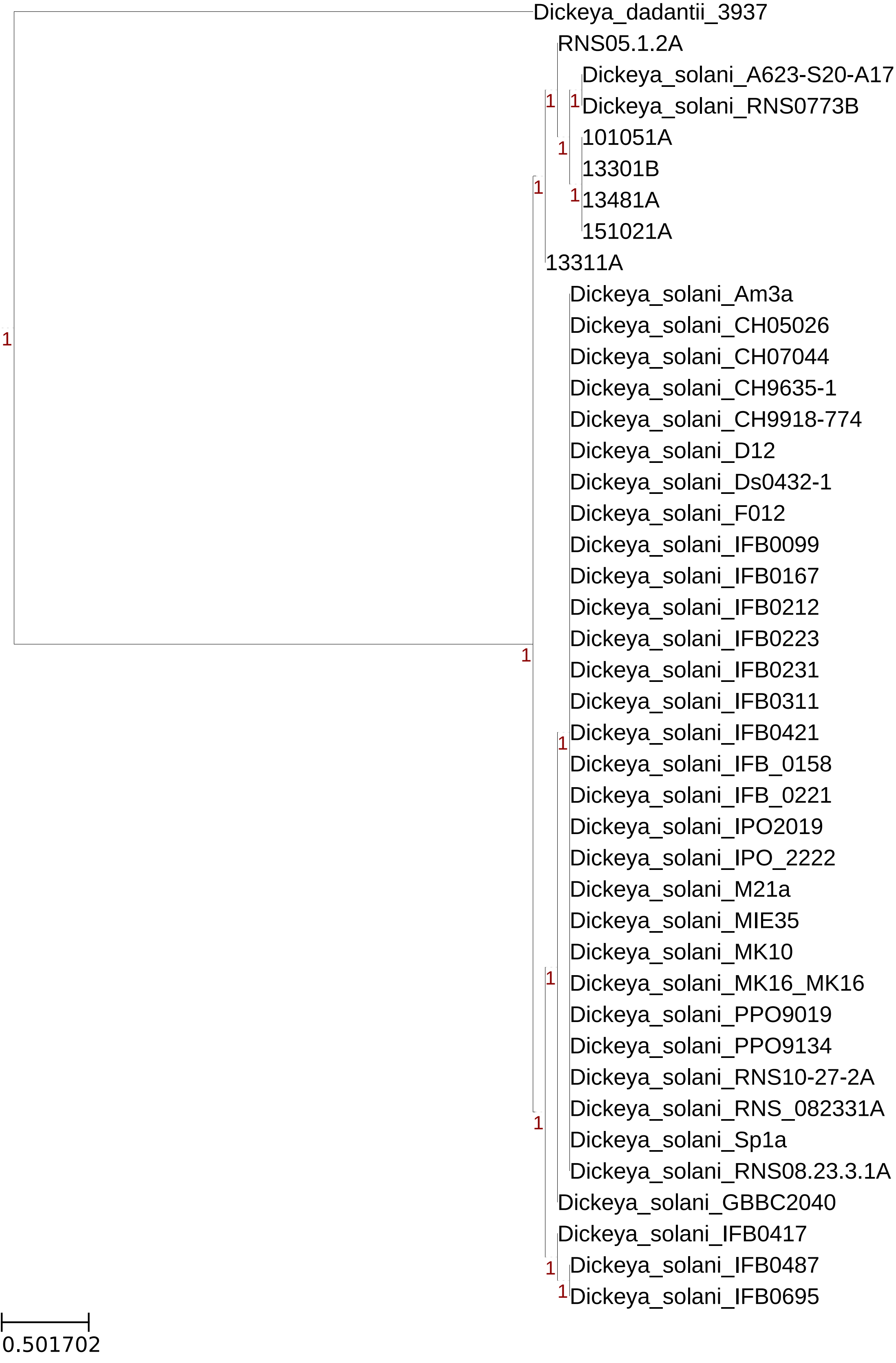
Dickeya_dadantii_3937
RNS05.1.2A
Dickeya_solani_A623-S20-A17
Dickeya_solani_RNS0773B
101051A
13301B
13481A
151021A
13311A
Dickeya_solani_Am3a
Dickeya_solani_CH05026
Dickeya_solani_CH07044
Dickeya_solani_CH9635-1
Dickeya_solani_CH9918-774
Dickeya_solani_D12
Dickeya_solani_Ds0432-1
Dickeya_solani_F012
Dickeya_solani_IFB0099
Dickeya_solani_IFB0167
Dickeya_solani_IFB0212
Dickeya_solani_IFB0223
Dickeya_solani_IFB0231
Dickeya_solani_IFB0311
Dickeya_solani_IFB0421
Dickeya_solani_IFB_0158
Dickeya_solani_IFB_0221
Dickeya_solani_IPO2019
Dickeya_solani_IPO_2222
Dickeya_solani_M21a
Dickeya_solani_MIE35
Dickeya_solani_MK10
Dickeya_solani_MK16_MK16
Dickeya_solani_PPO9019
Dickeya_solani_PPO9134
Dickeya_solani_RNS10-27-2A
Dickeya_solani_RNS_082331A
Dickeya_solani_Sp1a
Dickeya_solani_RNS08.23.3.1A
Dickeya_solani_GBBC2040
Dickeya_solani_IFB0417
Dickeya_solani_IFB0487
Dickeya_solani_IFB0695
1
1
1
1
1
1
1
1
1
1
1
1
0.501702

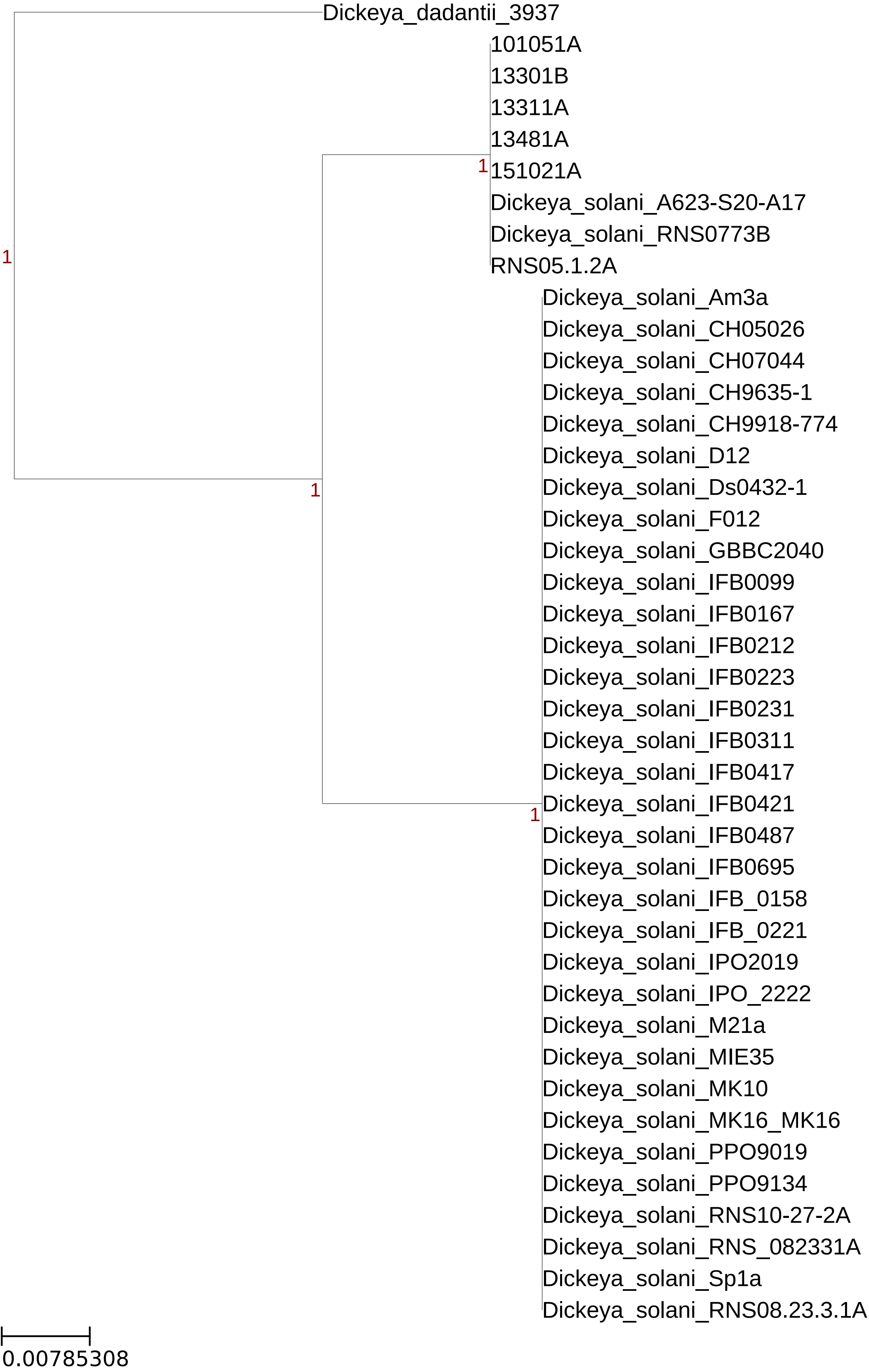

Dickeya_dadantii_3937
101051A
13301B
13311A
13481A
151021A
Dickeya_solani_A623-S20-A17
Dickeya_solani_RNS0773B
RNS05.1.2A
Dickeya_solani_Am3a
Dickeya_solani_CH05026
Dickeya_solani_CH07044
Dickeya_solani_CH9635-1
Dickeya_solani_CH9918-774
Dickeya_solani_D12
Dickeya_solani_Ds0432-1
Dickeya_solani_F012
Dickeya_solani_GBBC2040
Dickeya_solani_IFB0099
Dickeya_solani_IFB0167
Dickeya_solani_IFB0212
Dickeya_solani_IFB0223
Dickeya_solani_IFB0231
Dickeya_solani_IFB0311
Dickeya_solani_IFB0417
Dickeya_solani_IFB0421
Dickeya_solani_IFB0487
Dickeya_solani_IFB0695
Dickeya_solani_IFB_0158
Dickeya_solani_IFB_0221
Dickeya_solani_IPO2019
Dickeya_solani_IPO_2222
Dickeya_solani_M21a
Dickeya_solani_MIE35
Dickeya_solani_MK10
Dickeya_solani_MK16_MK16
Dickeya_solani_PPO9019
Dickeya_solani_PPO9134
Dickeya_solani_RNS10-27-2A
Dickeya_solani_RNS_082331A
Dickeya_solani_Sp1a
Dickeya_solani_RNS08.23.3.1A
1
1
1
1
0.00785308

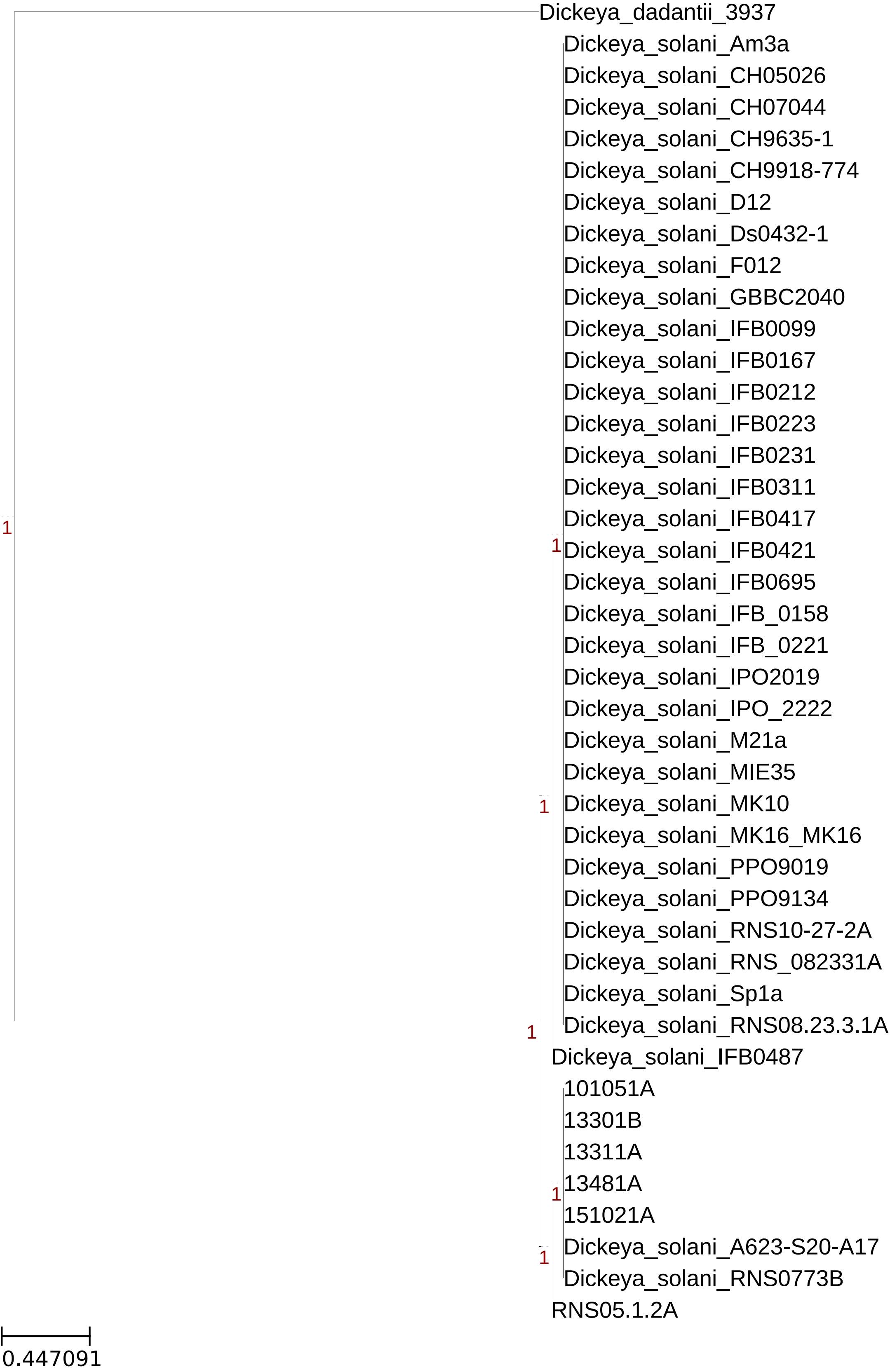
Dickeya_dadantii_3937
Dickeya_solani_Am3a
Dickeya_solani_CH05026
Dickeya_solani_CH07044
Dickeya_solani_CH9635-1
Dickeya_solani_CH9918-774
Dickeya_solani_D12
Dickeya_solani_Ds0432-1
Dickeya_solani_F012
Dickeya_solani_GBBC2040
Dickeya_solani_IFB0099
Dickeya_solani_IFB0167
Dickeya_solani_IFB0212
Dickeya_solani_IFB0223
Dickeya_solani_IFB0231
Dickeya_solani_IFB0311
Dickeya_solani_IFB0417
Dickeya_solani_IFB0421
Dickeya_solani_IFB0695
Dickeya_solani_IFB_0158
Dickeya_solani_IFB_0221
Dickeya_solani_IPO2019
Dickeya_solani_IPO_2222
Dickeya_solani_M21a
Dickeya_solani_MIE35
Dickeya_solani_MK10
Dickeya_solani_MK16_MK16
Dickeya_solani_PPO9019
Dickeya_solani_PPO9134
Dickeya_solani_RNS10-27-2A
Dickeya_solani_RNS_082331A
Dickeya_solani_Sp1a
Dickeya_solani_RNS08.23.3.1A
Dickeya_solani_IFB0487
101051A
13301B
13311A
13481A
151021A
Dickeya_solani_A623-S20-A17
Dickeya_solani_RNS0773B
RNS05.1.2A
1
1
1
1
1
1
0.447091

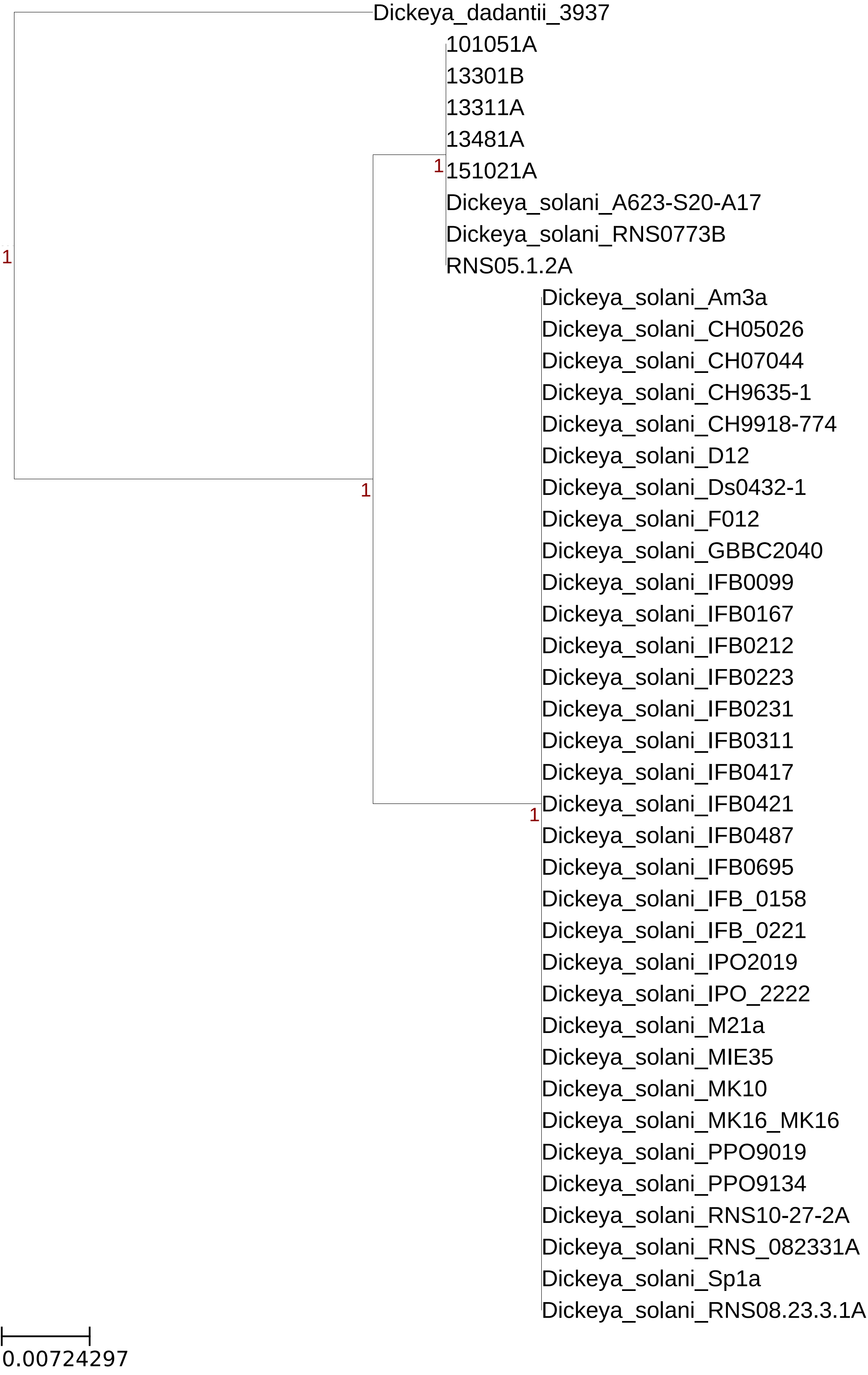

Dickeya_dadantii_3937
101051A
13301B
13311A
13481A
151021A
Dickeya_solani_A623-S20-A17
Dickeya_solani_RNS0773B
RNS05.1.2A
Dickeya_solani_Am3a
Dickeya_solani_CH05026
Dickeya_solani_CH07044
Dickeya_solani_CH9635-1
Dickeya_solani_CH9918-774
Dickeya_solani_D12
Dickeya_solani_Ds0432-1
Dickeya_solani_F012
Dickeya_solani_GBBC2040
Dickeya_solani_IFB0099
Dickeya_solani_IFB0167
Dickeya_solani_IFB0212
Dickeya_solani_IFB0223
Dickeya_solani_IFB0231
Dickeya_solani_IFB0311
Dickeya_solani_IFB0417
Dickeya_solani_IFB0421
Dickeya_solani_IFB0487
Dickeya_solani_IFB0695
Dickeya_solani_IFB_0158
Dickeya_solani_IFB_0221
Dickeya_solani_IPO2019
Dickeya_solani_IPO_2222
Dickeya_solani_M21a
Dickeya_solani_MIE35
Dickeya_solani_MK10
Dickeya_solani_MK16_MK16
Dickeya_solani_PPO9019
Dickeya_solani_PPO9134
Dickeya_solani_RNS10-27-2A
Dickeya_solani_RNS_082331A
Dickeya_solani_Sp1a
Dickeya_solani_RNS08.23.3.1A
1
1
1
1
0.00724297

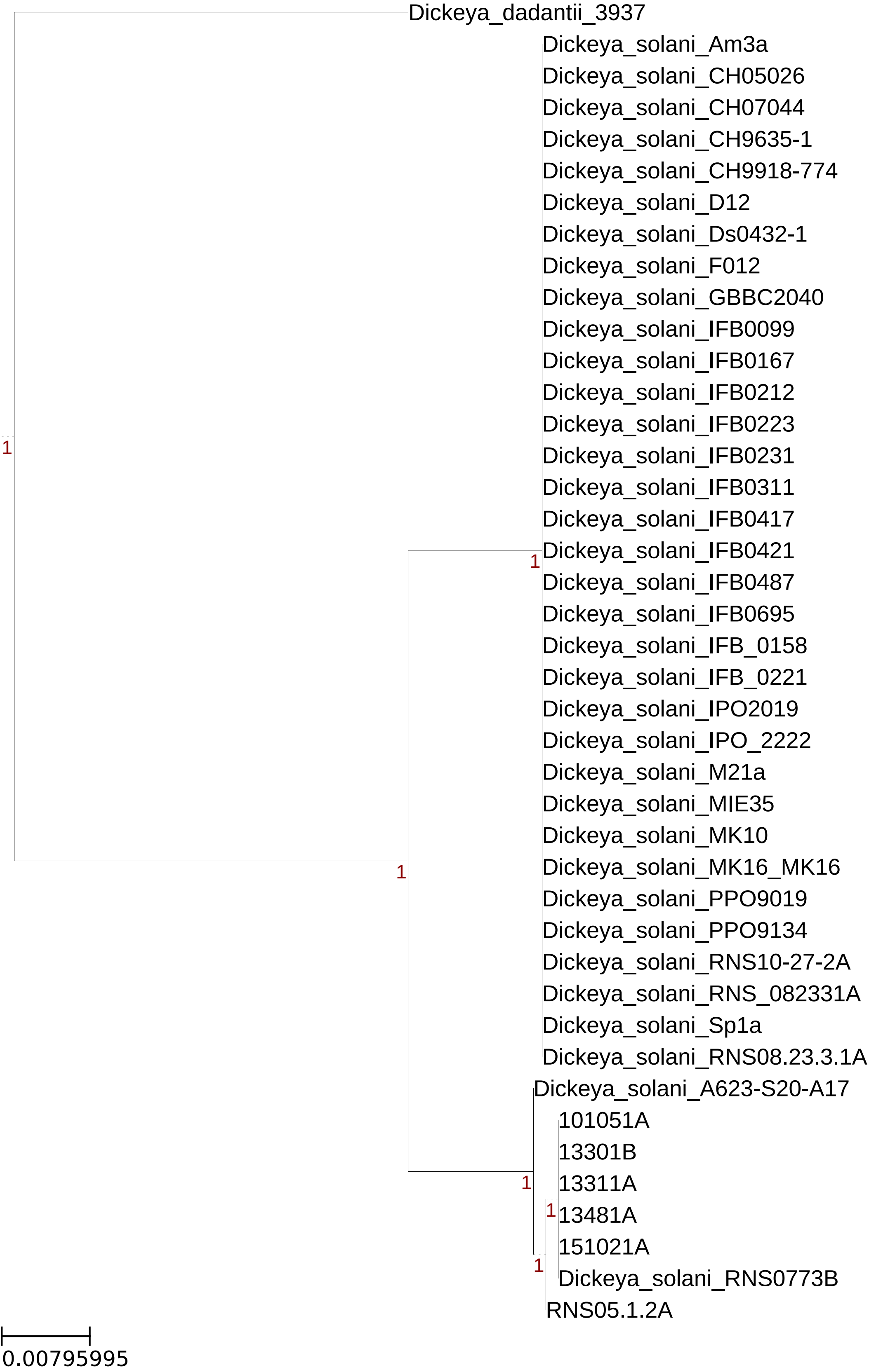
Dickeya_dadantii_3937
Dickeya_solani_Am3a
Dickeya_solani_CH05026
Dickeya_solani_CH07044
Dickeya_solani_CH9635-1
Dickeya_solani_CH9918-774
Dickeya_solani_D12
Dickeya_solani_Ds0432-1
Dickeya_solani_F012
Dickeya_solani_GBBC2040
Dickeya_solani_IFB0099
Dickeya_solani_IFB0167
Dickeya_solani_IFB0212
Dickeya_solani_IFB0223
Dickeya_solani_IFB0231
Dickeya_solani_IFB0311
Dickeya_solani_IFB0417
Dickeya_solani_IFB0421
Dickeya_solani_IFB0487
Dickeya_solani_IFB0695
Dickeya_solani_IFB_0158
Dickeya_solani_IFB_0221
Dickeya_solani_IPO2019
Dickeya_solani_IPO_2222
Dickeya_solani_M21a
Dickeya_solani_MIE35
Dickeya_solani_MK10
Dickeya_solani_MK16_MK16
Dickeya_solani_PPO9019
Dickeya_solani_PPO9134
Dickeya_solani_RNS10-27-2A
Dickeya_solani_RNS_082331A
Dickeya_solani_Sp1a
Dickeya_solani_RNS08.23.3.1A
Dickeya_solani_A623-S20-A17
101051A
13301B
13311A
13481A
151021A
Dickeya_solani_RNS0773B
RNS05.1.2A
1
1
1
1
1
1
0.00795995

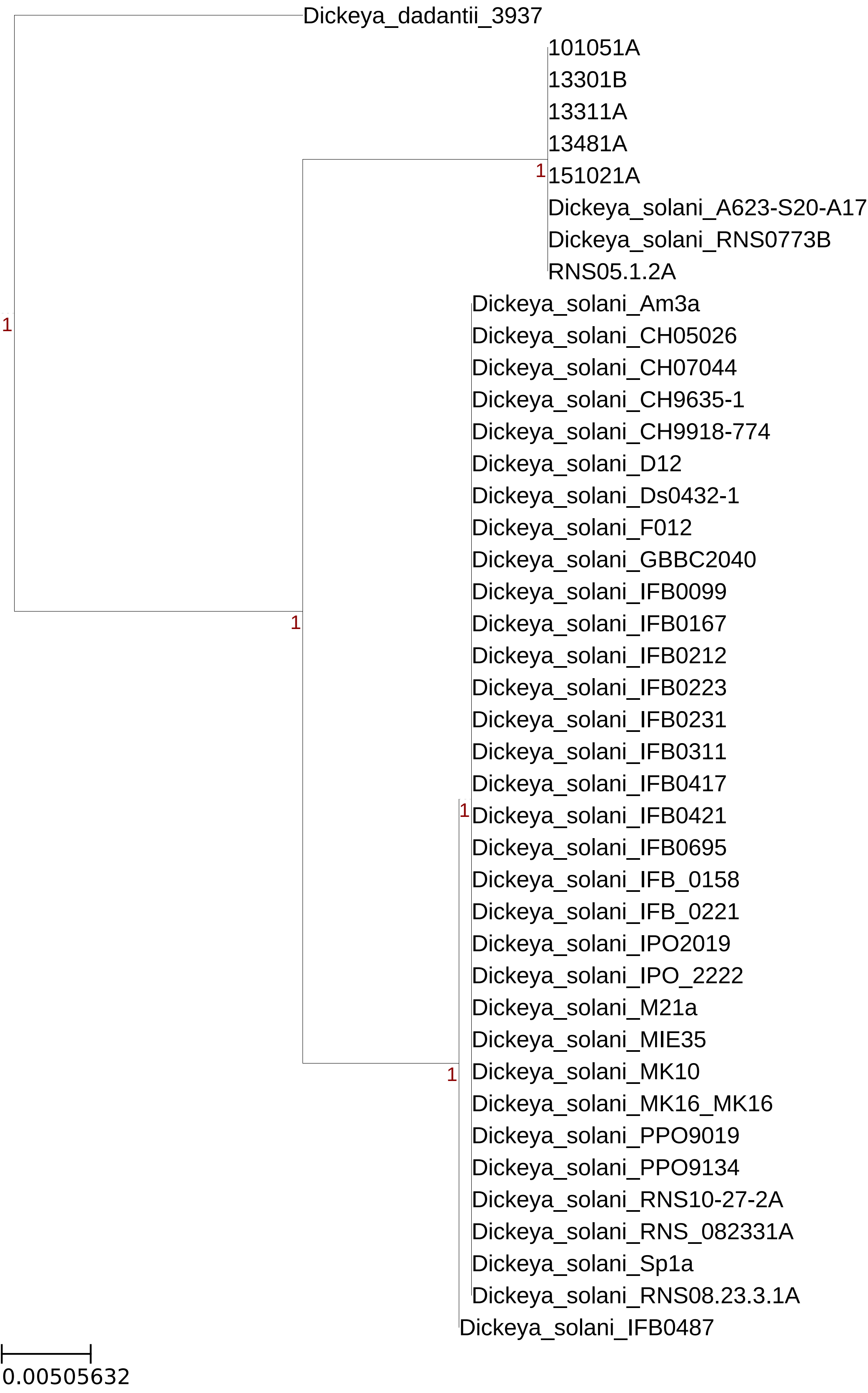
Dickeya_dadantii_3937
101051A
13301B
13311A
13481A
151021A
Dickeya_solani_A623-S20-A17
Dickeya_solani_RNS0773B
RNS05.1.2A
Dickeya_solani_Am3a
Dickeya_solani_CH05026
Dickeya_solani_CH07044
Dickeya_solani_CH9635-1
Dickeya_solani_CH9918-774
Dickeya_solani_D12
Dickeya_solani_Ds0432-1
Dickeya_solani_F012
Dickeya_solani_GBBC2040
Dickeya_solani_IFB0099
Dickeya_solani_IFB0167
Dickeya_solani_IFB0212
Dickeya_solani_IFB0223
Dickeya_solani_IFB0231
Dickeya_solani_IFB0311
Dickeya_solani_IFB0417
Dickeya_solani_IFB0421
Dickeya_solani_IFB0695
Dickeya_solani_IFB_0158
Dickeya_solani_IFB_0221
Dickeya_solani_IPO2019
Dickeya_solani_IPO_2222
Dickeya_solani_M21a
Dickeya_solani_MIE35
Dickeya_solani_MK10
Dickeya_solani_MK16_MK16
Dickeya_solani_PPO9019
Dickeya_solani_PPO9134
Dickeya_solani_RNS10-27-2A
Dickeya_solani_RNS_082331A
Dickeya_solani_Sp1a
Dickeya_solani_RNS08.23.3.1A
Dickeya_solani_IFB0487
1
1
1
1
1
0.00505632

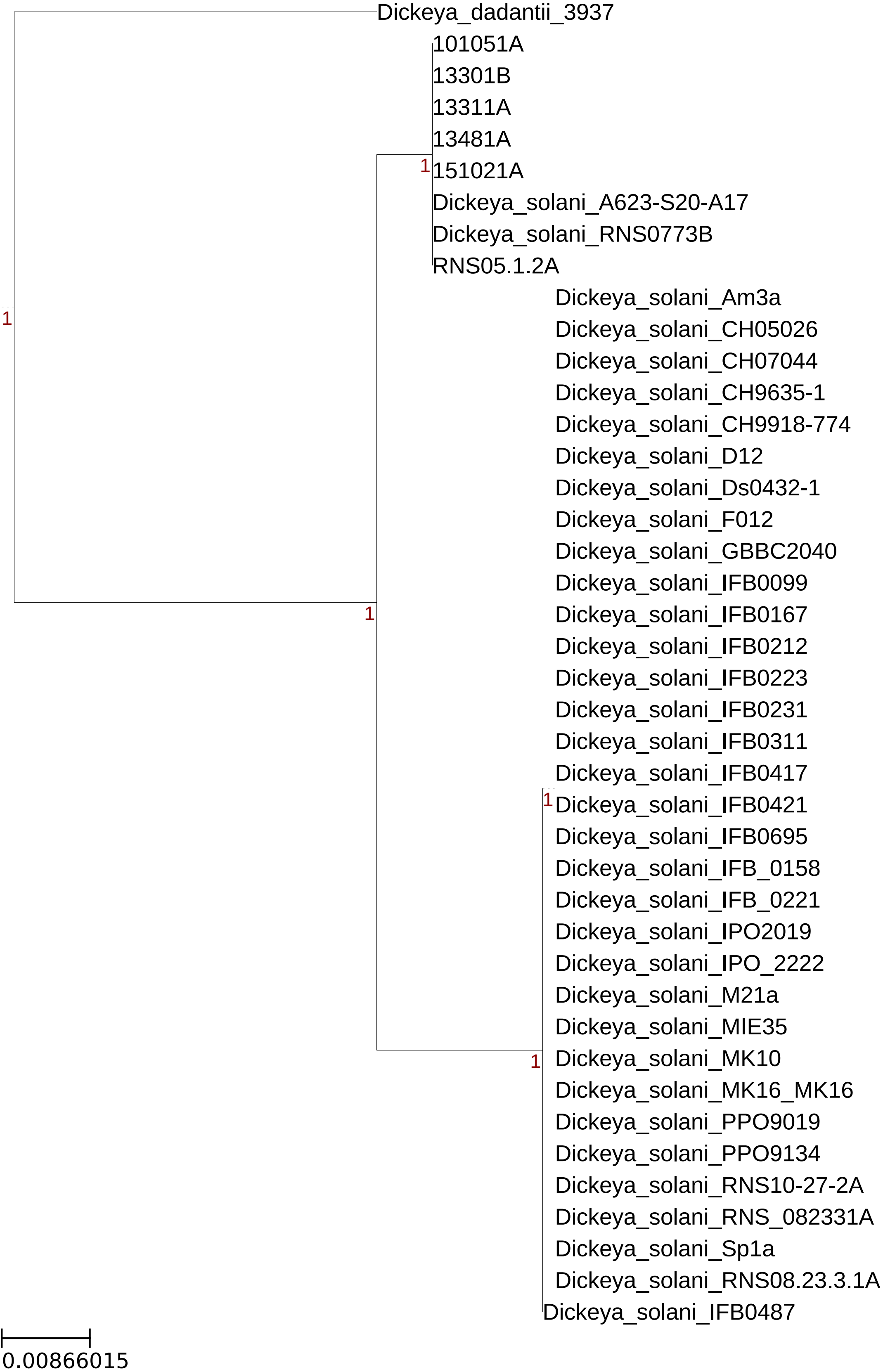
Dickeya_dadantii_3937
101051A
13301B
13311A
13481A
1
151021A
Dickeya_solani_A623-S20-A17
Dickeya_solani_RNS0773B
RNS05.1.2A
1
Dickeya_solani_Am3a
Dickeya_solani_CH05026
Dickeya_solani_CH07044
Dickeya_solani_CH9635-1
Dickeya_solani_CH9918-774
Dickeya_solani_D12
Dickeya_solani_Ds0432-1
Dickeya_solani_F012
Dickeya_solani_GBBC2040
Dickeya_solani_IFB0099
1
Dickeya_solani_IFB0167
Dickeya_solani_IFB0212
Dickeya_solani_IFB0223
Dickeya_solani_IFB0231
Dickeya_solani_IFB0311
Dickeya_solani_IFB0417
1
Dickeya_solani_IFB0421
Dickeya_solani_IFB0695
Dickeya_solani_IFB_0158
Dickeya_solani_IFB_0221
Dickeya_solani_IPO2019
Dickeya_solani_IPO_2222
Dickeya_solani_M21a
Dickeya_solani_MIE35
1
Dickeya_solani_MK10
Dickeya_solani_MK16_MK16
Dickeya_solani_PPO9019
Dickeya_solani_PPO9134
Dickeya_solani_RNS10-27-2A
Dickeya_solani_RNS_082331A
Dickeya_solani_Sp1a
Dickeya_solani_RNS08.23.3.1A
Dickeya_solani_IFB0487
0.00866015

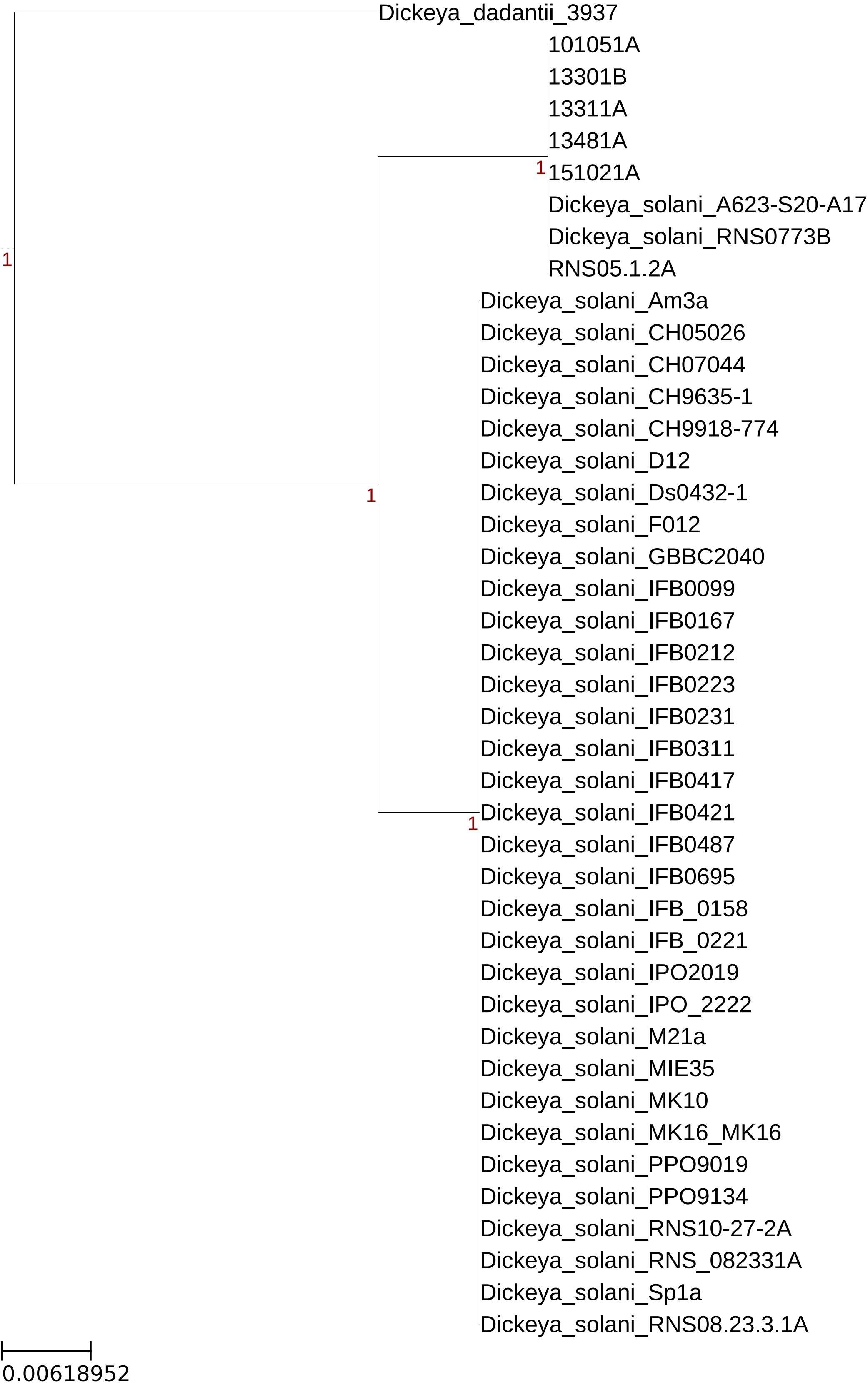
Dickeya_dadantii_3937
101051A
13301B
13311A
13481A
151021A
Dickeya_solani_A623-S20-A17
Dickeya_solani_RNS0773B
RNS05.1.2A
Dickeya_solani_Am3a
Dickeya_solani_CH05026
Dickeya_solani_CH07044
Dickeya_solani_CH9635-1
Dickeya_solani_CH9918-774
Dickeya_solani_D12
Dickeya_solani_Ds0432-1
Dickeya_solani_F012
Dickeya_solani_GBBC2040
Dickeya_solani_IFB0099
Dickeya_solani_IFB0167
Dickeya_solani_IFB0212
Dickeya_solani_IFB0223
Dickeya_solani_IFB0231
Dickeya_solani_IFB0311
Dickeya_solani_IFB0417
Dickeya_solani_IFB0421
Dickeya_solani_IFB0487
Dickeya_solani_IFB0695
Dickeya_solani_IFB_0158
Dickeya_solani_IFB_0221
Dickeya_solani_IPO2019
Dickeya_solani_IPO_2222
Dickeya_solani_M21a
Dickeya_solani_MIE35
Dickeya_solani_MK10
Dickeya_solani_MK16_MK16
Dickeya_solani_PPO9019
Dickeya_solani_PPO9134
Dickeya_solani_RNS10-27-2A
Dickeya_solani_RNS_082331A
Dickeya_solani_Sp1a
Dickeya_solani_RNS08.23.3.1A
1
1
1
1
0.00618952

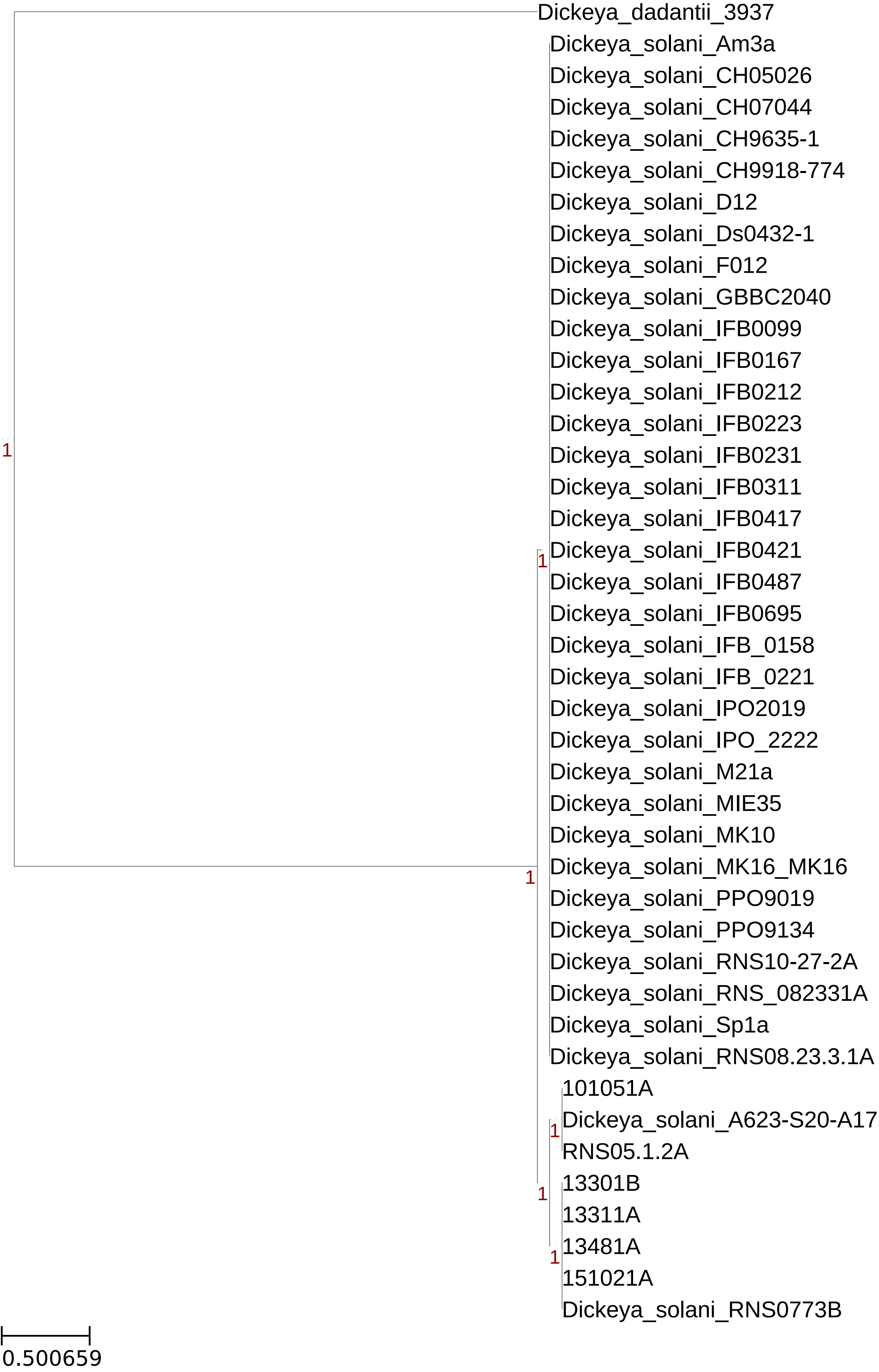
Dickeya_dadantii_3937
Dickeya_solani_Am3a
Dickeya_solani_CH05026
Dickeya_solani_CH07044
Dickeya_solani_CH9635-1
Dickeya_solani_CH9918-774
Dickeya_solani_D12
Dickeya_solani_Ds0432-1
Dickeya_solani_F012
Dickeya_solani_GBBC2040
Dickeya_solani_IFB0099
Dickeya_solani_IFB0167
Dickeya_solani_IFB0212
Dickeya_solani_IFB0223
Dickeya_solani_IFB0231
Dickeya_solani_IFB0311
Dickeya_solani_IFB0417
Dickeya_solani_IFB0421
Dickeya_solani_IFB0487
Dickeya_solani_IFB0695
Dickeya_solani_IFB_0158
Dickeya_solani_IFB_0221
Dickeya_solani_IPO2019
Dickeya_solani_IPO_2222
Dickeya_solani_M21a
Dickeya_solani_MIE35
Dickeya_solani_MK10
Dickeya_solani_MK16_MK16
Dickeya_solani_PPO9019
Dickeya_solani_PPO9134
Dickeya_solani_RNS10-27-2A
Dickeya_solani_RNS_082331A
Dickeya_solani_Sp1a
Dickeya_solani_RNS08.23.3.1A
101051A
Dickeya_solani_A623-S20-A17
RNS05.1.2A
13301B
13311A
13481A
151021A
Dickeya_solani_RNS0773B
1
1
1
1
1
1
0.500659

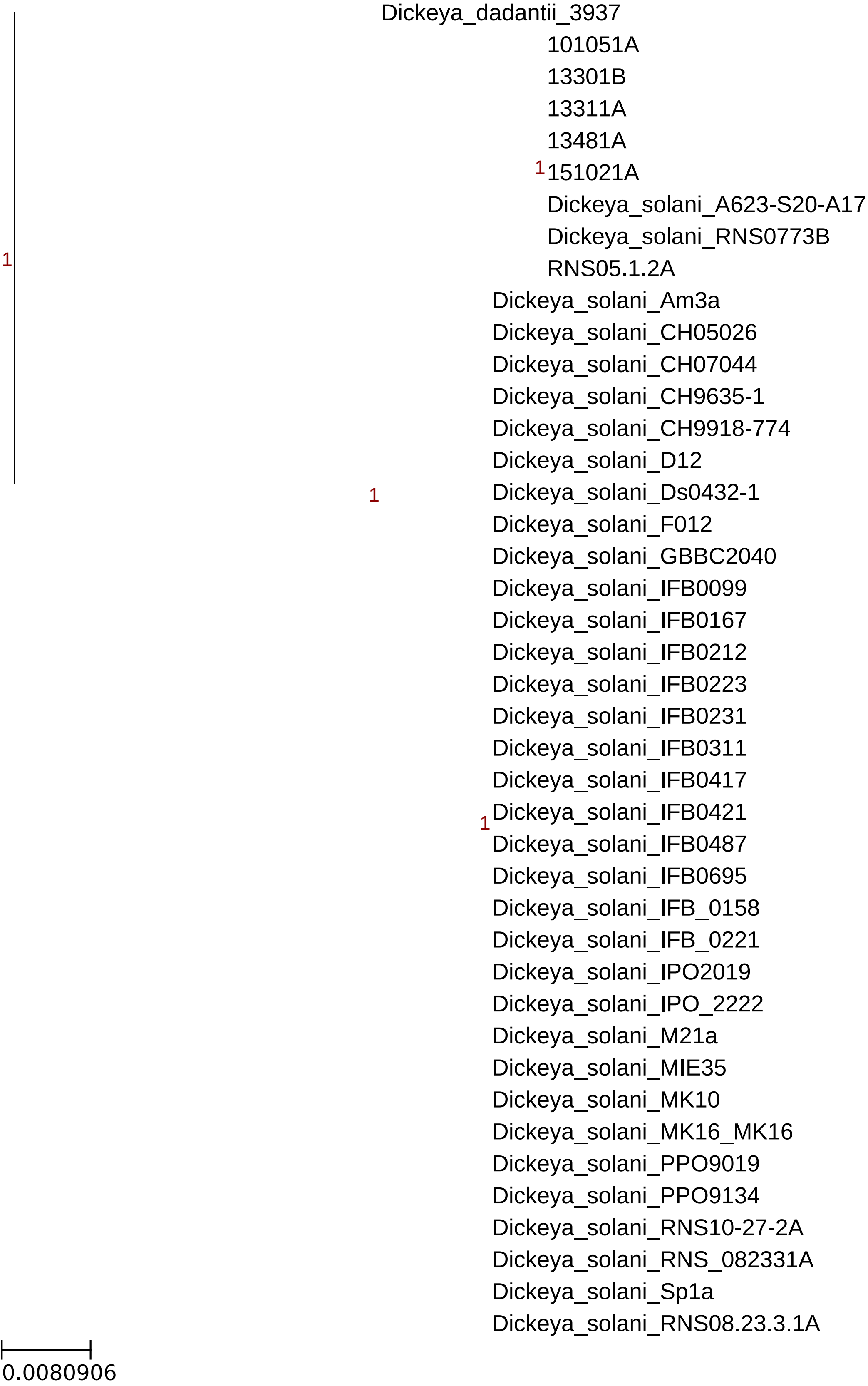
Dickeya_dadantii_3937
101051A
13301B
13311A
13481A
151021A
Dickeya_solani_A623-S20-A17
Dickeya_solani_RNS0773B
RNS05.1.2A
Dickeya_solani_Am3a
Dickeya_solani_CH05026
Dickeya_solani_CH07044
Dickeya_solani_CH9635-1
Dickeya_solani_CH9918-774
Dickeya_solani_D12
Dickeya_solani_Ds0432-1
Dickeya_solani_F012
Dickeya_solani_GBBC2040
Dickeya_solani_IFB0099
Dickeya_solani_IFB0167
Dickeya_solani_IFB0212
Dickeya_solani_IFB0223
Dickeya_solani_IFB0231
Dickeya_solani_IFB0311
Dickeya_solani_IFB0417
Dickeya_solani_IFB0421
Dickeya_solani_IFB0487
Dickeya_solani_IFB0695
Dickeya_solani_IFB_0158
Dickeya_solani_IFB_0221
Dickeya_solani_IPO2019
Dickeya_solani_IPO_2222
Dickeya_solani_M21a
Dickeya_solani_MIE35
Dickeya_solani_MK10
Dickeya_solani_MK16_MK16
Dickeya_solani_PPO9019
Dickeya_solani_PPO9134
Dickeya_solani_RNS10-27-2A
Dickeya_solani_RNS_082331A
Dickeya_solani_Sp1a
Dickeya_solani_RNS08.23.3.1A
1
1
1
1
0.0080906

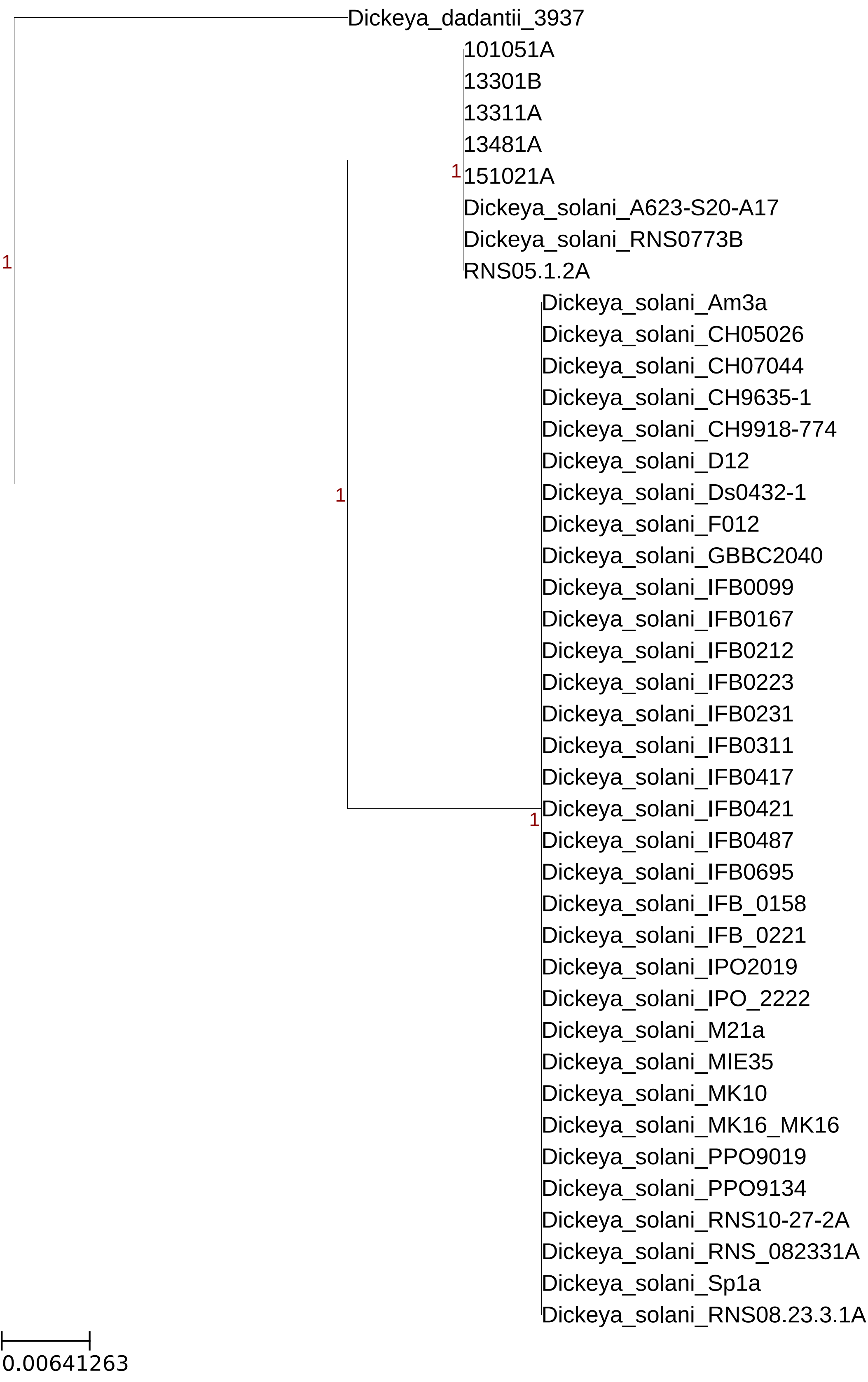
Dickeya_dadantii_3937
101051A
13301B
13311A
13481A
151021A
Dickeya_solani_A623-S20-A17
Dickeya_solani_RNS0773B
RNS05.1.2A
Dickeya_solani_Am3a
Dickeya_solani_CH05026
Dickeya_solani_CH07044
Dickeya_solani_CH9635-1
Dickeya_solani_CH9918-774
Dickeya_solani_D12
Dickeya_solani_Ds0432-1
Dickeya_solani_F012
Dickeya_solani_GBBC2040
Dickeya_solani_IFB0099
Dickeya_solani_IFB0167
Dickeya_solani_IFB0212
Dickeya_solani_IFB0223
Dickeya_solani_IFB0231
Dickeya_solani_IFB0311
Dickeya_solani_IFB0417
Dickeya_solani_IFB0421
Dickeya_solani_IFB0487
Dickeya_solani_IFB0695
Dickeya_solani_IFB_0158
Dickeya_solani_IFB_0221
Dickeya_solani_IPO2019
Dickeya_solani_IPO_2222
Dickeya_solani_M21a
Dickeya_solani_MIE35
Dickeya_solani_MK10
Dickeya_solani_MK16_MK16
Dickeya_solani_PPO9019
Dickeya_solani_PPO9134
Dickeya_solani_RNS10-27-2A
Dickeya_solani_RNS_082331A
Dickeya_solani_Sp1a
Dickeya_solani_RNS08.23.3.1A
1
1
1
1
0.00641263

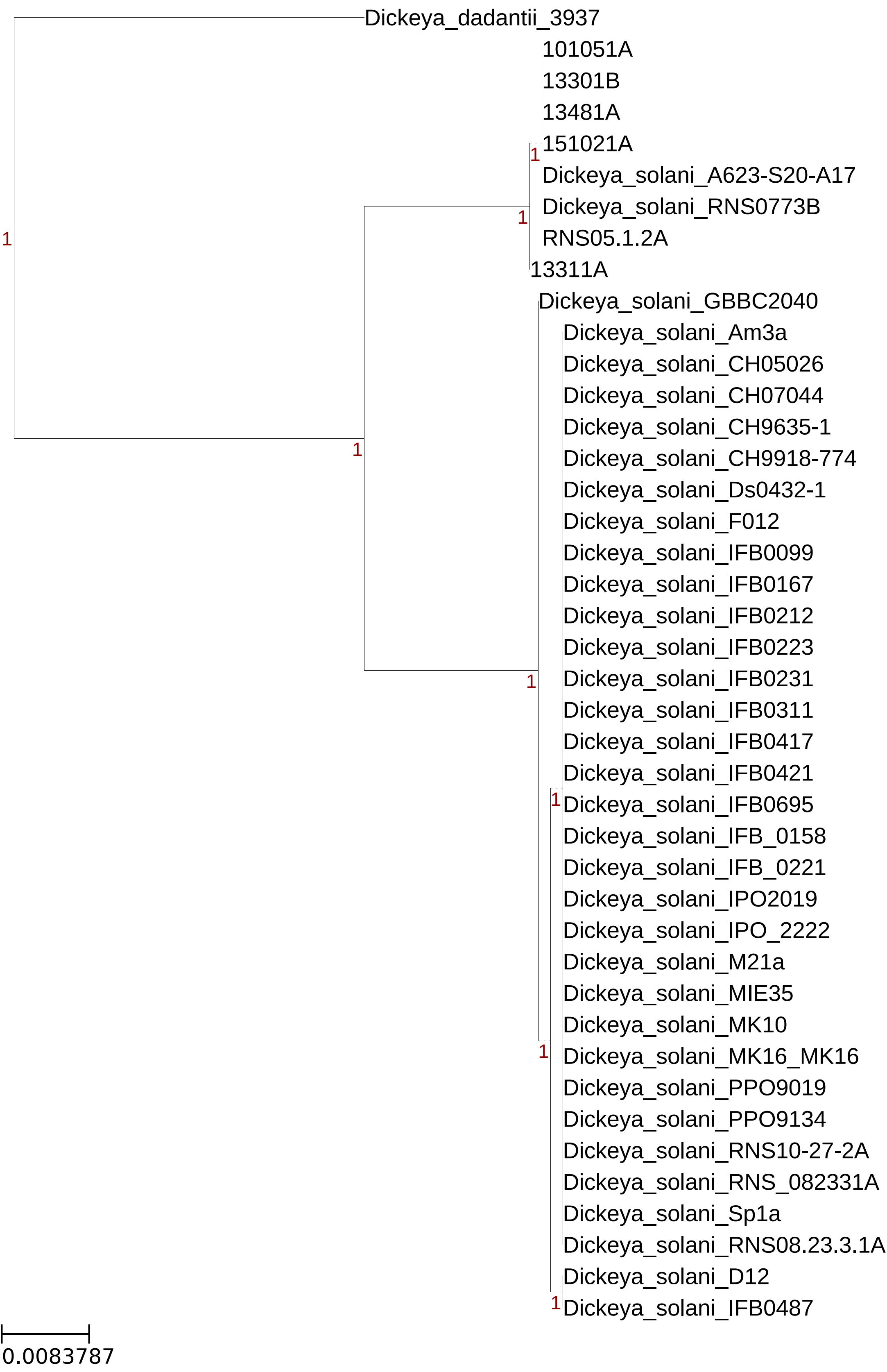
Dickeya_dadantii_3937
101051A
13301B
13481A
151021A
Dickeya_solani_A623-S20-A17
Dickeya_solani_RNS0773B
RNS05.1.2A
13311A
Dickeya_solani_GBBC2040
Dickeya_solani_Am3a
Dickeya_solani_CH05026
Dickeya_solani_CH07044
Dickeya_solani_CH9635-1
Dickeya_solani_CH9918-774
Dickeya_solani_Ds0432-1
Dickeya_solani_F012
Dickeya_solani_IFB0099
Dickeya_solani_IFB0167
Dickeya_solani_IFB0212
Dickeya_solani_IFB0223
Dickeya_solani_IFB0231
Dickeya_solani_IFB0311
Dickeya_solani_IFB0417
Dickeya_solani_IFB0421
Dickeya_solani_IFB0695
Dickeya_solani_IFB_0158
Dickeya_solani_IFB_0221
Dickeya_solani_IPO2019
Dickeya_solani_IPO_2222
Dickeya_solani_M21a
Dickeya_solani_MIE35
Dickeya_solani_MK10
Dickeya_solani_MK16_MK16
Dickeya_solani_PPO9019
Dickeya_solani_PPO9134
Dickeya_solani_RNS10-27-2A
Dickeya_solani_RNS_082331A
Dickeya_solani_Sp1a
Dickeya_solani_RNS08.23.3.1A
Dickeya_solani_D12
Dickeya_solani_IFB0487
1
0.0083787

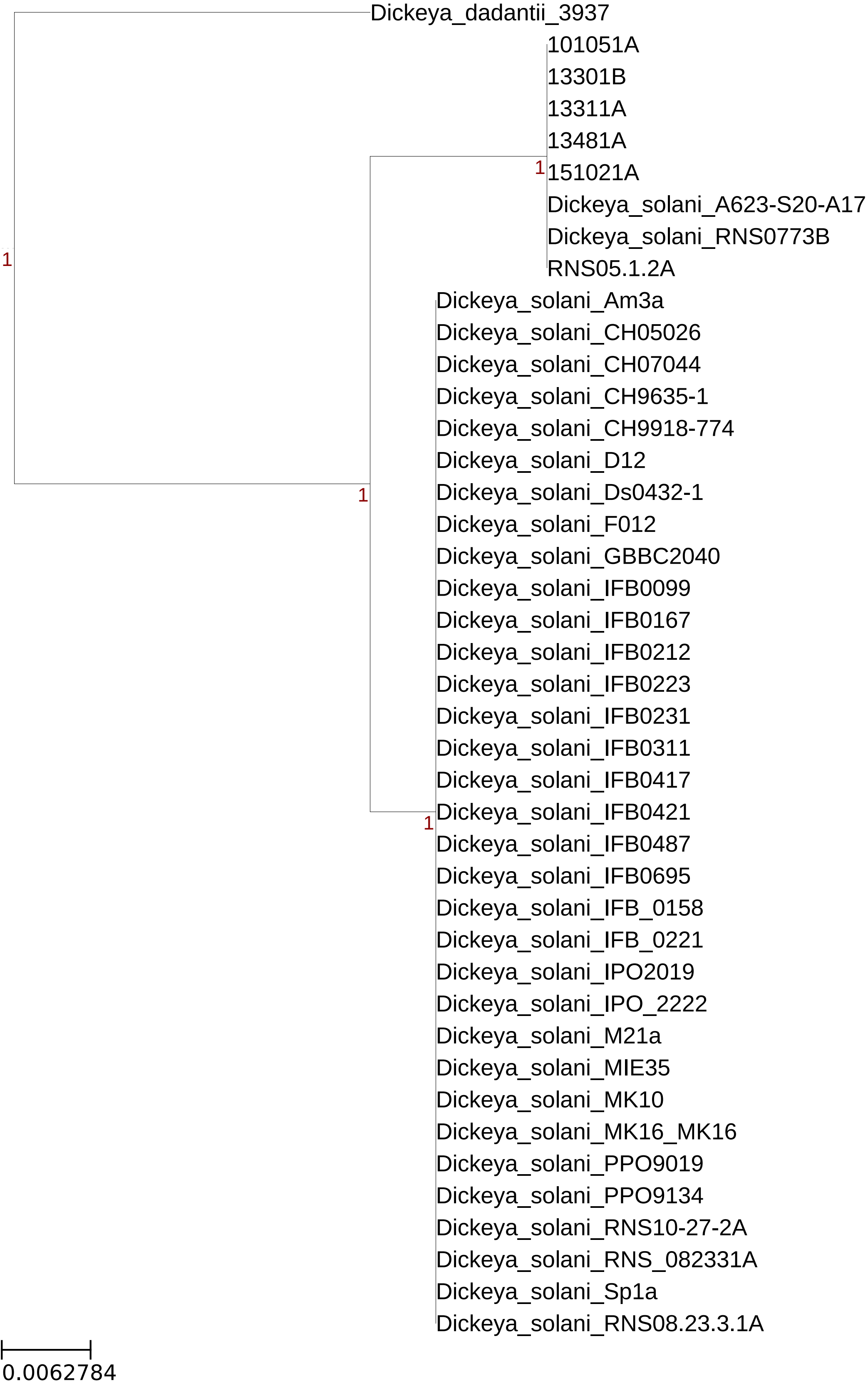
Dickeya_dadantii_3937
101051A
13301B
13311A
13481A
151021A
Dickeya_solani_A623-S20-A17
Dickeya_solani_RNS0773B
RNS05.1.2A
Dickeya_solani_Am3a
Dickeya_solani_CH05026
Dickeya_solani_CH07044
Dickeya_solani_CH9635-1
Dickeya_solani_CH9918-774
Dickeya_solani_D12
Dickeya_solani_Ds0432-1
Dickeya_solani_F012
Dickeya_solani_GBBC2040
Dickeya_solani_IFB0099
Dickeya_solani_IFB0167
Dickeya_solani_IFB0212
Dickeya_solani_IFB0223
Dickeya_solani_IFB0231
Dickeya_solani_IFB0311
Dickeya_solani_IFB0417
Dickeya_solani_IFB0421
Dickeya_solani_IFB0487
Dickeya_solani_IFB0695
Dickeya_solani_IFB_0158
Dickeya_solani_IFB_0221
Dickeya_solani_IPO2019
Dickeya_solani_IPO_2222
Dickeya_solani_M21a
Dickeya_solani_MIE35
Dickeya_solani_MK10
Dickeya_solani_MK16_MK16
Dickeya_solani_PPO9019
Dickeya_solani_PPO9134
Dickeya_solani_RNS10-27-2A
Dickeya_solani_RNS_082331A
Dickeya_solani_Sp1a
Dickeya_solani_RNS08.23.3.1A
1
1
1
1
0.0062784

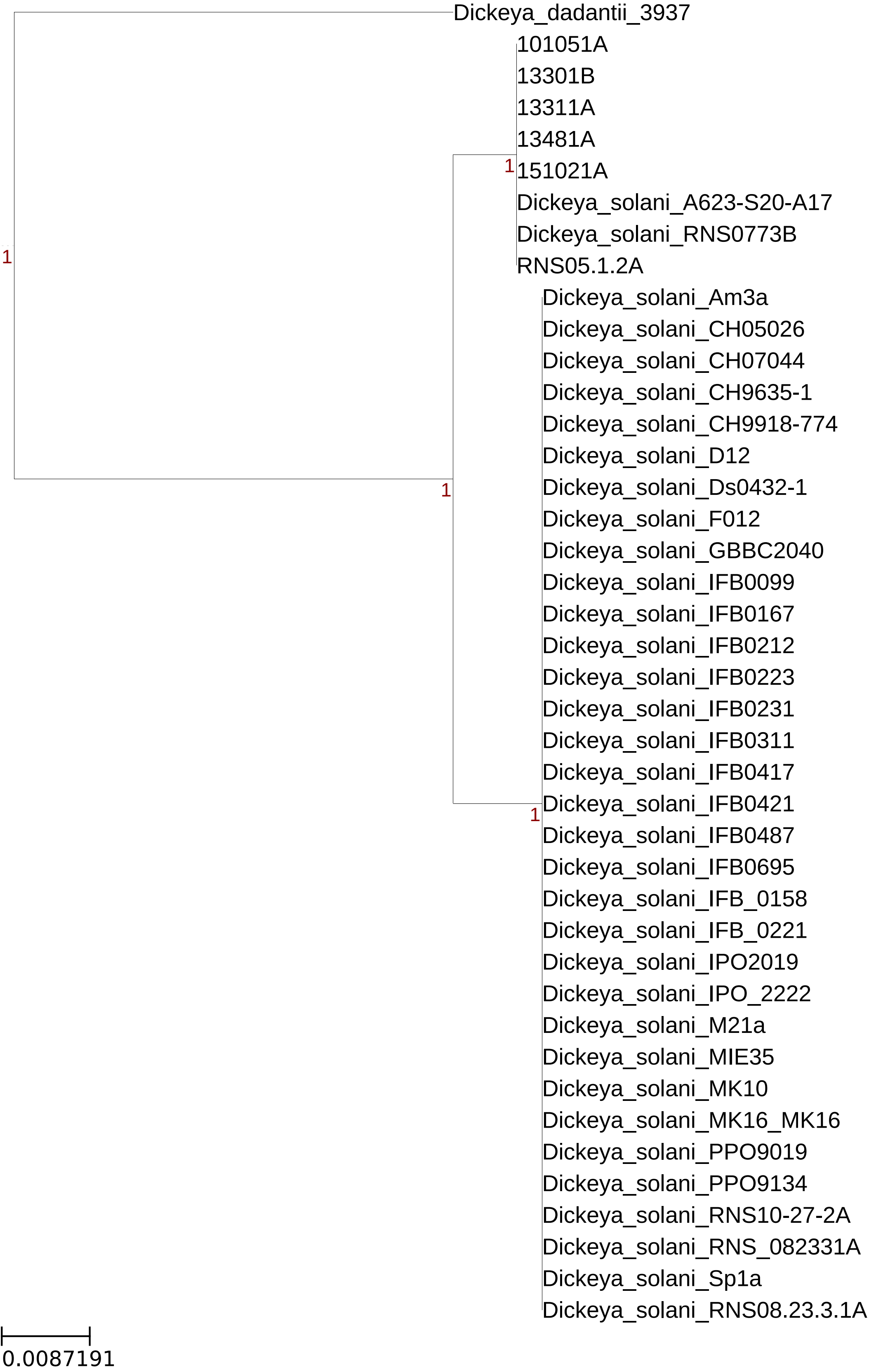
Dickeya_dadantii_3937
101051A
13301B
13311A
13481A
151021A
Dickeya_solani_A623-S20-A17
Dickeya_solani_RNS0773B
RNS05.1.2A
Dickeya_solani_Am3a
Dickeya_solani_CH05026
Dickeya_solani_CH07044
Dickeya_solani_CH9635-1
Dickeya_solani_CH9918-774
Dickeya_solani_D12
Dickeya_solani_Ds0432-1
Dickeya_solani_F012
Dickeya_solani_GBBC2040
Dickeya_solani_IFB0099
Dickeya_solani_IFB0167
Dickeya_solani_IFB0212
Dickeya_solani_IFB0223
Dickeya_solani_IFB0231
Dickeya_solani_IFB0311
Dickeya_solani_IFB0417
Dickeya_solani_IFB0421
Dickeya_solani_IFB0487
Dickeya_solani_IFB0695
Dickeya_solani_IFB_0158
Dickeya_solani_IFB_0221
Dickeya_solani_IPO2019
Dickeya_solani_IPO_2222
Dickeya_solani_M21a
Dickeya_solani_MIE35
Dickeya_solani_MK10
Dickeya_solani_MK16_MK16
Dickeya_solani_PPO9019
Dickeya_solani_PPO9134
Dickeya_solani_RNS10-27-2A
Dickeya_solani_RNS_082331A
Dickeya_solani_Sp1a
Dickeya_solani_RNS08.23.3.1A
1
1
1
1
0.0087191

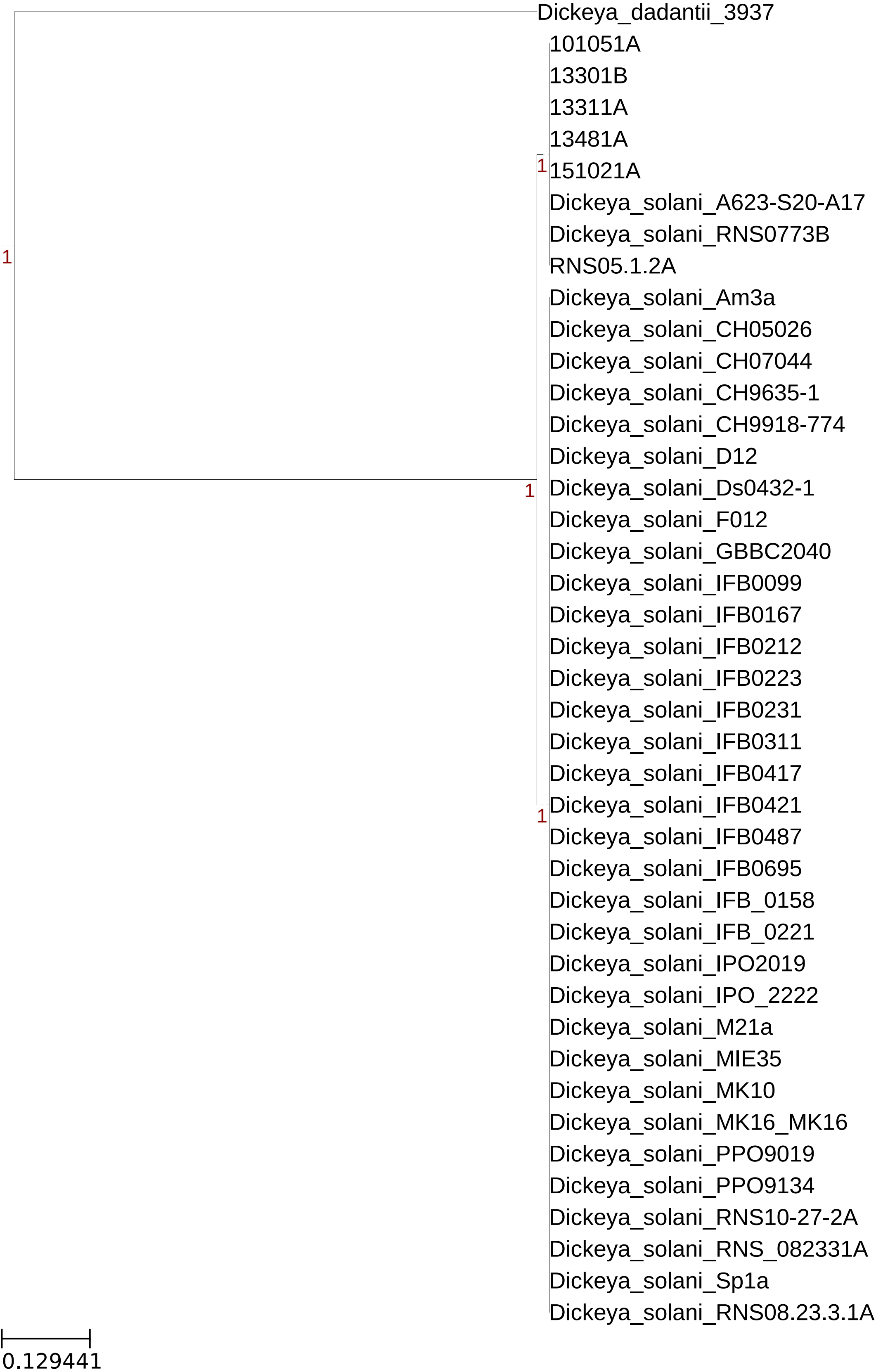
Dickeya_dadantii_3937
101051A
13301B
13311A
13481A
151021A
Dickeya_solani_A623-S20-A17
Dickeya_solani_RNS0773B
RNS05.1.2A
Dickeya_solani_Am3a
Dickeya_solani_CH05026
Dickeya_solani_CH07044
Dickeya_solani_CH9635-1
Dickeya_solani_CH9918-774
Dickeya_solani_D12
Dickeya_solani_Ds0432-1
Dickeya_solani_F012
Dickeya_solani_GBBC2040
Dickeya_solani_IFB0099
Dickeya_solani_IFB0167
Dickeya_solani_IFB0212
Dickeya_solani_IFB0223
Dickeya_solani_IFB0231
Dickeya_solani_IFB0311
Dickeya_solani_IFB0417
Dickeya_solani_IFB0421
Dickeya_solani_IFB0487
Dickeya_solani_IFB0695
Dickeya_solani_IFB_0158
Dickeya_solani_IFB_0221
Dickeya_solani_IPO2019
Dickeya_solani_IPO_2222
Dickeya_solani_M21a
Dickeya_solani_MIE35
Dickeya_solani_MK10
Dickeya_solani_MK16_MK16
Dickeya_solani_PPO9019
Dickeya_solani_PPO9134
Dickeya_solani_RNS10-27-2A
Dickeya_solani_RNS_082331A
Dickeya_solani_Sp1a
Dickeya_solani_RNS08.23.3.1A
1
1
1
1
0.129441

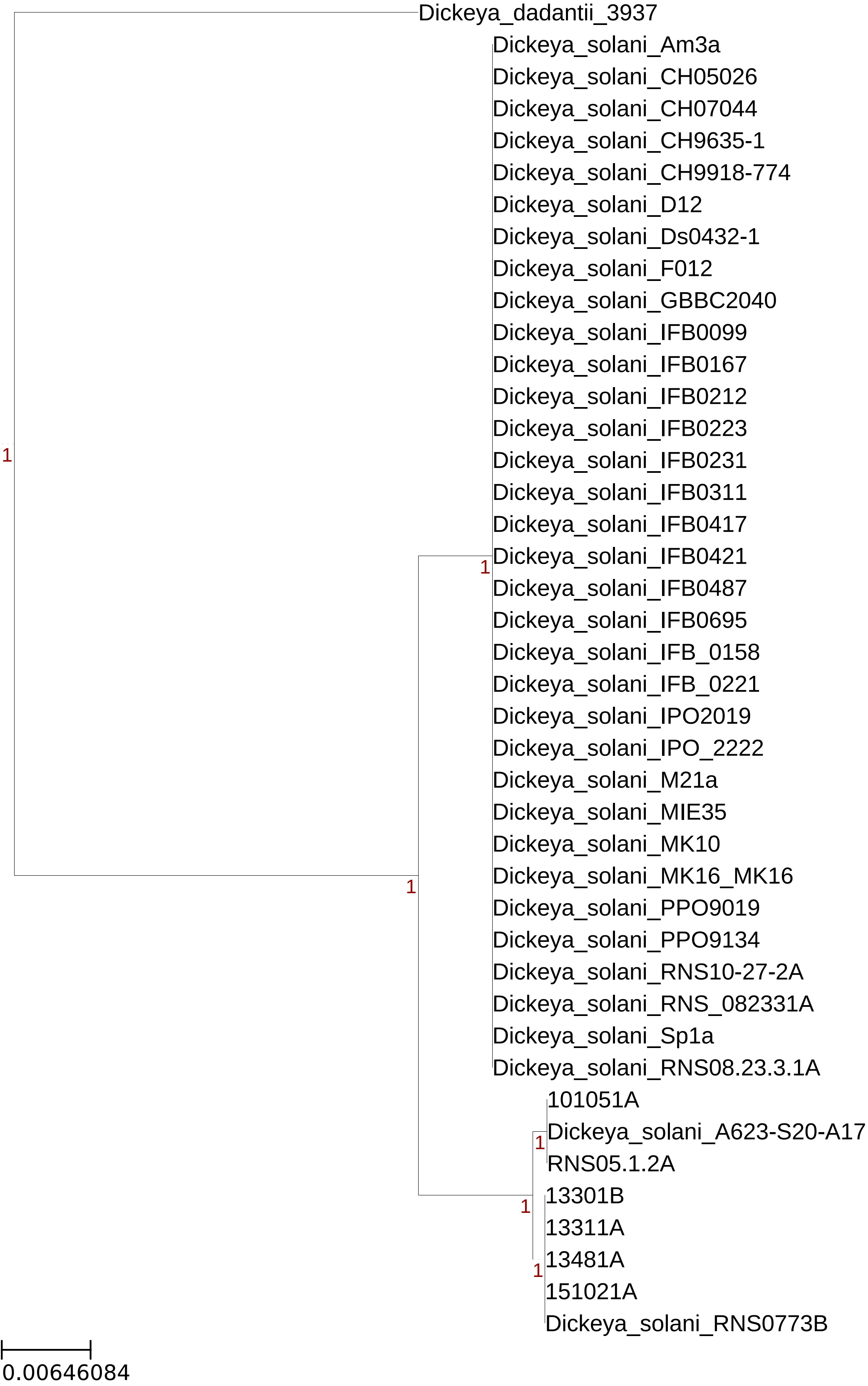
Dickeya_dadantii_3937
Dickeya_solani_Am3a
Dickeya_solani_CH05026
Dickeya_solani_CH07044
Dickeya_solani_CH9635-1
Dickeya_solani_CH9918-774
Dickeya_solani_D12
Dickeya_solani_Ds0432-1
Dickeya_solani_F012
Dickeya_solani_GBBC2040
Dickeya_solani_IFB0099
Dickeya_solani_IFB0167
Dickeya_solani_IFB0212
Dickeya_solani_IFB0223
Dickeya_solani_IFB0231
Dickeya_solani_IFB0311
Dickeya_solani_IFB0417
Dickeya_solani_IFB0421
Dickeya_solani_IFB0487
Dickeya_solani_IFB0695
Dickeya_solani_IFB_0158
Dickeya_solani_IFB_0221
Dickeya_solani_IPO2019
Dickeya_solani_IPO_2222
Dickeya_solani_M21a
Dickeya_solani_MIE35
Dickeya_solani_MK10
Dickeya_solani_MK16_MK16
Dickeya_solani_PPO9019
Dickeya_solani_PPO9134
Dickeya_solani_RNS10-27-2A
Dickeya_solani_RNS_082331A
Dickeya_solani_Sp1a
Dickeya_solani_RNS08.23.3.1A
101051A
Dickeya_solani_A623-S20-A17
RNS05.1.2A
13301B
13311A
13481A
151021A
Dickeya_solani_RNS0773B
1
1
1
1
1
1
0.00646084

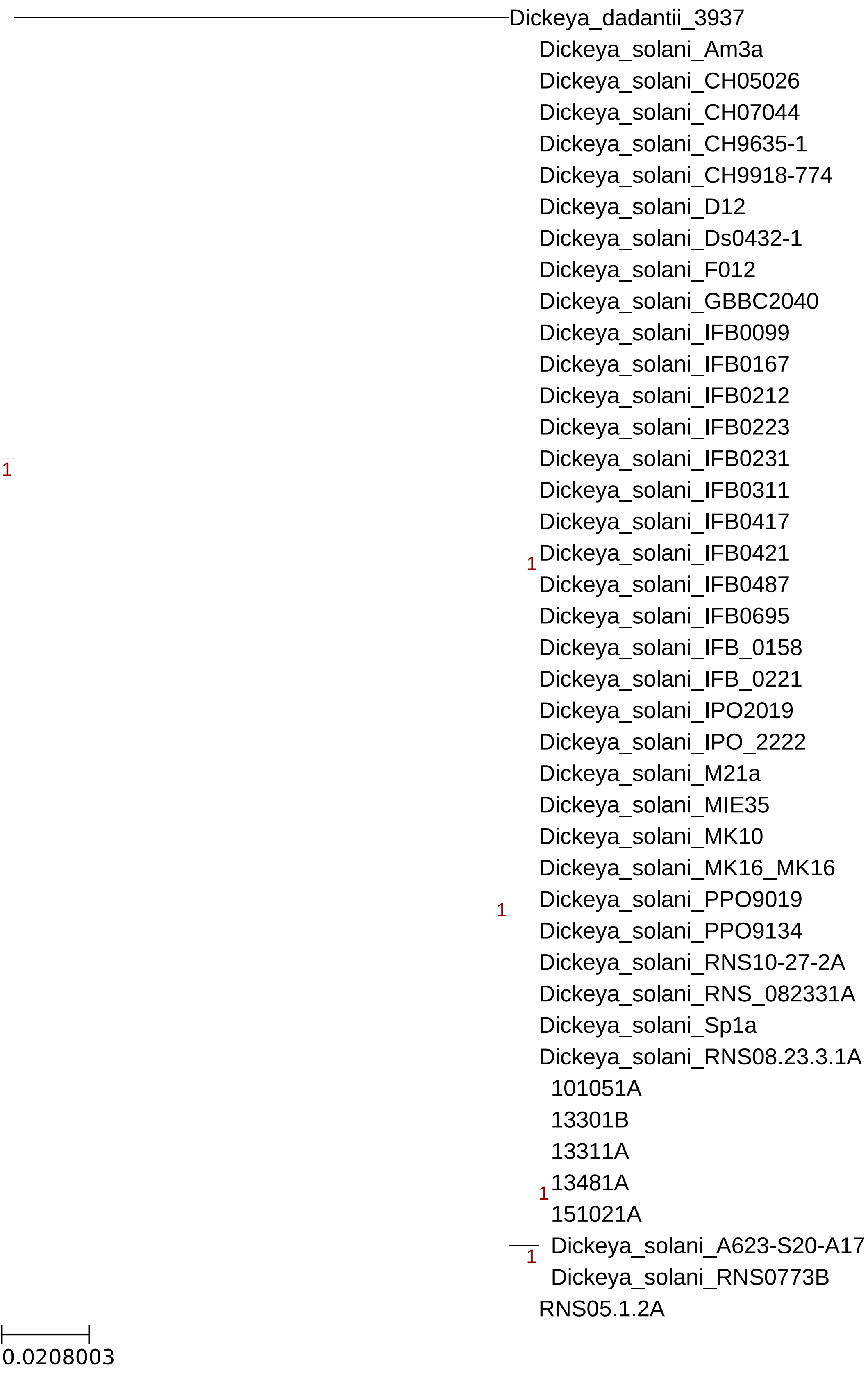

Dickeya_dadantii_3937
Dickeya_solani_Am3a
Dickeya_solani_CH05026
Dickeya_solani_CH07044
Dickeya_solani_CH9635-1
Dickeya_solani_CH9918-774
Dickeya_solani_D12
Dickeya_solani_Ds0432-1
Dickeya_solani_F012
Dickeya_solani_GBBC2040
Dickeya_solani_IFB0099
Dickeya_solani_IFB0167
Dickeya_solani_IFB0212
Dickeya_solani_IFB0223
Dickeya_solani_IFB0231
Dickeya_solani_IFB0311
Dickeya_solani_IFB0417
Dickeya_solani_IFB0421
Dickeya_solani_IFB0487
Dickeya_solani_IFB0695
Dickeya_solani_IFB_0158
Dickeya_solani_IFB_0221
Dickeya_solani_IPO2019
Dickeya_solani_IPO_2222
Dickeya_solani_M21a
Dickeya_solani_MIE35
Dickeya_solani_MK10
Dickeya_solani_MK16_MK16
Dickeya_solani_PPO9019
Dickeya_solani_PPO9134
Dickeya_solani_RNS10-27-2A
Dickeya_solani_RNS_082331A
Dickeya_solani_Sp1a
Dickeya_solani_RNS08.23.3.1A
101051A
13301B
13311A
13481A
151021A
Dickeya_solani_A623-S20-A17
Dickeya_solani_RNS0773B
RNS05.1.2A
1
1
1
1
1
0.0208003

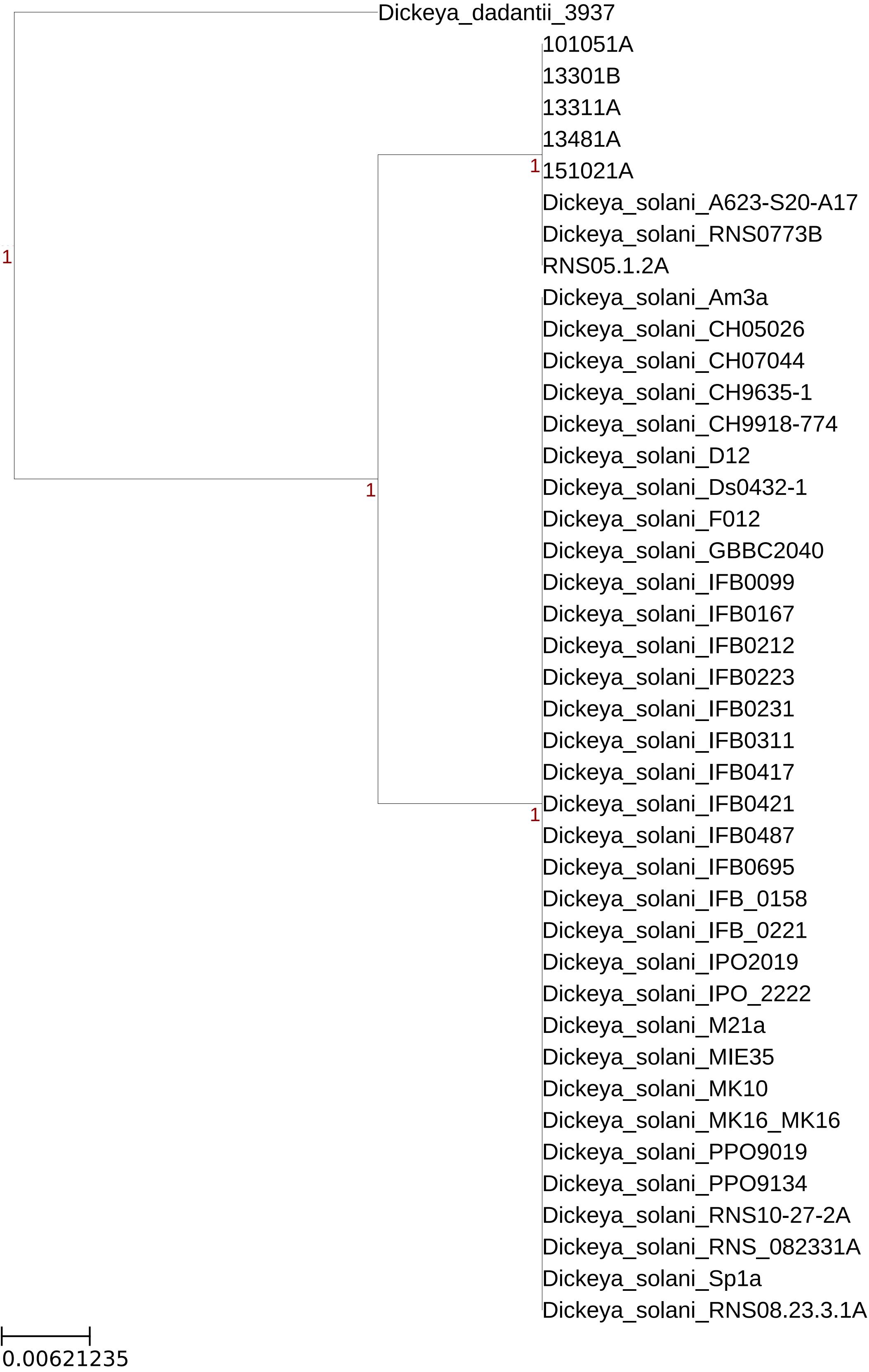
Dickeya_dadantii_3937
101051A
13301B
13311A
13481A
151021A
Dickeya_solani_A623-S20-A17
Dickeya_solani_RNS0773B
RNS05.1.2A
Dickeya_solani_Am3a
Dickeya_solani_CH05026
Dickeya_solani_CH07044
Dickeya_solani_CH9635-1
Dickeya_solani_CH9918-774
Dickeya_solani_D12
Dickeya_solani_Ds0432-1
Dickeya_solani_F012
Dickeya_solani_GBBC2040
Dickeya_solani_IFB0099
Dickeya_solani_IFB0167
Dickeya_solani_IFB0212
Dickeya_solani_IFB0223
Dickeya_solani_IFB0231
Dickeya_solani_IFB0311
Dickeya_solani_IFB0417
Dickeya_solani_IFB0421
Dickeya_solani_IFB0487
Dickeya_solani_IFB0695
Dickeya_solani_IFB_0158
Dickeya_solani_IFB_0221
Dickeya_solani_IPO2019
Dickeya_solani_IPO_2222
Dickeya_solani_M21a
Dickeya_solani_MIE35
Dickeya_solani_MK10
Dickeya_solani_MK16_MK16
Dickeya_solani_PPO9019
Dickeya_solani_PPO9134
Dickeya_solani_RNS10-27-2A
Dickeya_solani_RNS_082331A
Dickeya_solani_Sp1a
Dickeya_solani_RNS08.23.3.1A
1
1
1
1
0.00621235

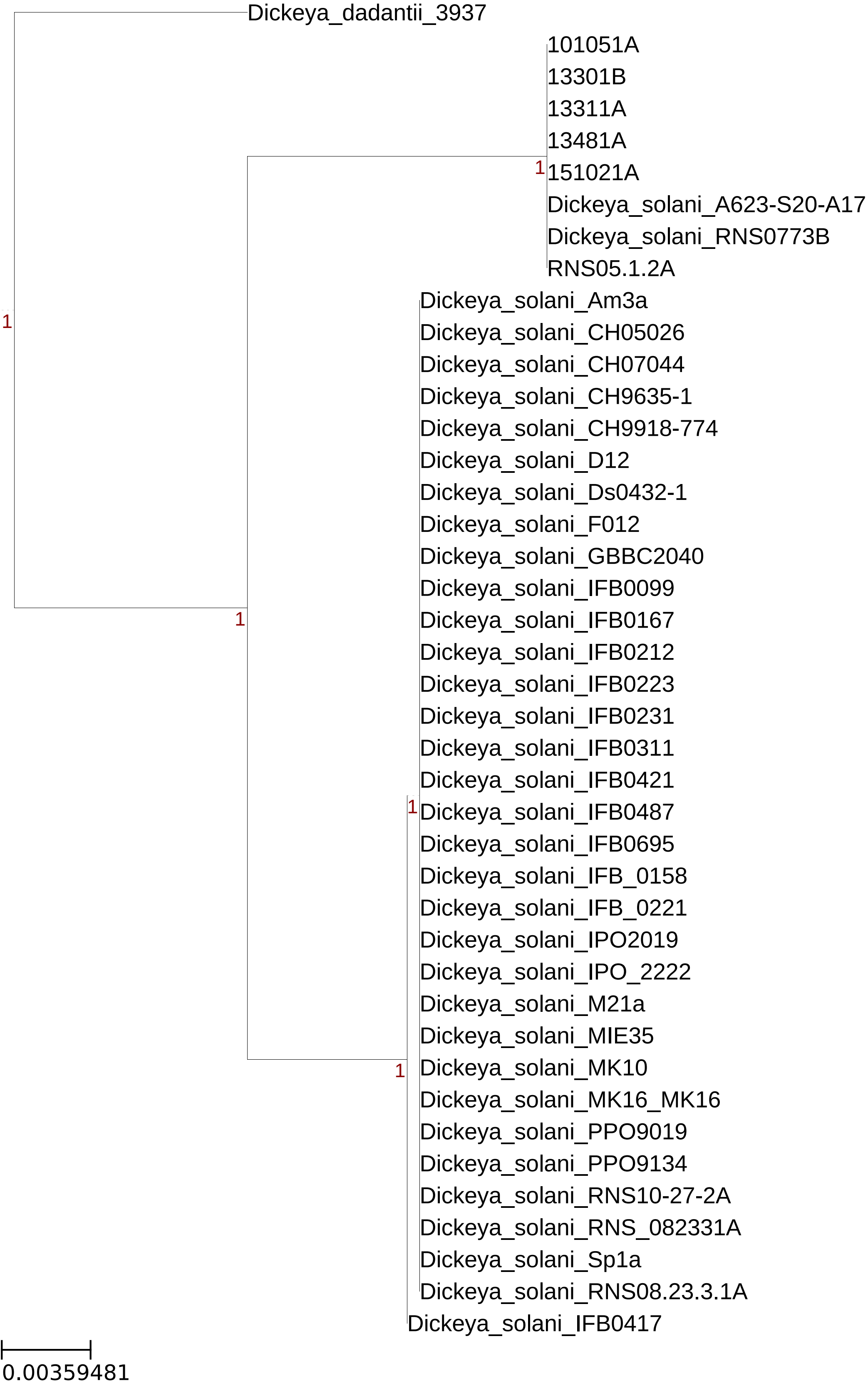

Dickeya_dadantii_3937
101051A
13301B
13311A
13481A
151021A
Dickeya_solani_A623-S20-A17
Dickeya_solani_RNS0773B
RNS05.1.2A
Dickeya_solani_Am3a
Dickeya_solani_CH05026
Dickeya_solani_CH07044
Dickeya_solani_CH9635-1
Dickeya_solani_CH9918-774
Dickeya_solani_D12
Dickeya_solani_Ds0432-1
Dickeya_solani_F012
Dickeya_solani_GBBC2040
Dickeya_solani_IFB0099
Dickeya_solani_IFB0167
Dickeya_solani_IFB0212
Dickeya_solani_IFB0223
Dickeya_solani_IFB0231
Dickeya_solani_IFB0311
Dickeya_solani_IFB0421
Dickeya_solani_IFB0487
Dickeya_solani_IFB0695
Dickeya_solani_IFB_0158
Dickeya_solani_IFB_0221
Dickeya_solani_IPO2019
Dickeya_solani_IPO_2222
Dickeya_solani_M21a
Dickeya_solani_MIE35
Dickeya_solani_MK10
Dickeya_solani_MK16_MK16
Dickeya_solani_PPO9019
Dickeya_solani_PPO9134
Dickeya_solani_RNS10-27-2A
Dickeya_solani_RNS_082331A
Dickeya_solani_Sp1a
Dickeya_solani_RNS08.23.3.1A
Dickeya_solani_IFB0417
1
1
1
1
1
0.00359481

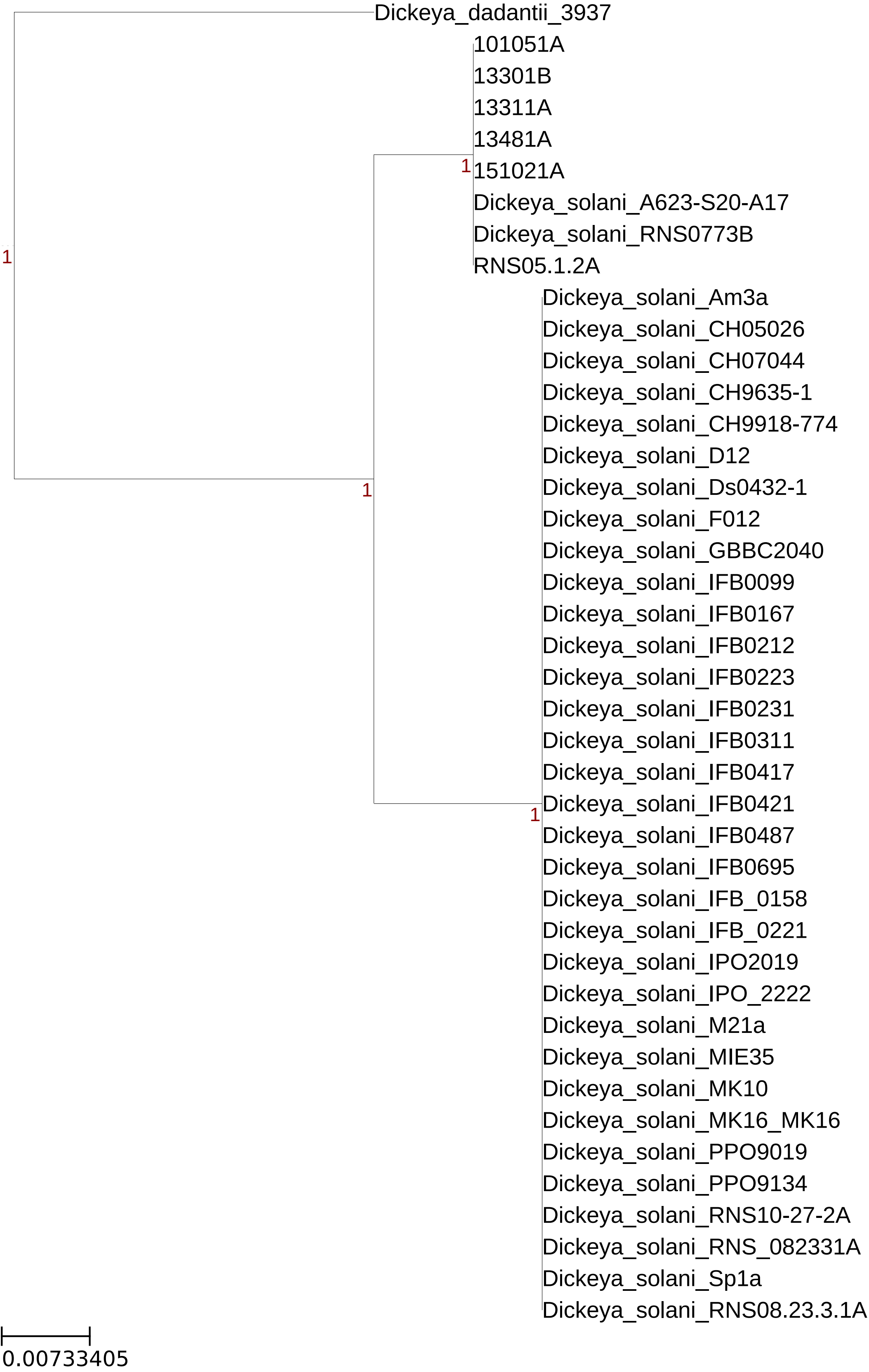
Dickeya_dadantii_3937
101051A
13301B
13311A
13481A
151021A
Dickeya_solani_A623-S20-A17
Dickeya_solani_RNS0773B
RNS05.1.2A
Dickeya_solani_Am3a
Dickeya_solani_CH05026
Dickeya_solani_CH07044
Dickeya_solani_CH9635-1
Dickeya_solani_CH9918-774
Dickeya_solani_D12
Dickeya_solani_Ds0432-1
Dickeya_solani_F012
Dickeya_solani_GBBC2040
Dickeya_solani_IFB0099
Dickeya_solani_IFB0167
Dickeya_solani_IFB0212
Dickeya_solani_IFB0223
Dickeya_solani_IFB0231
Dickeya_solani_IFB0311
Dickeya_solani_IFB0417
Dickeya_solani_IFB0421
Dickeya_solani_IFB0487
Dickeya_solani_IFB0695
Dickeya_solani_IFB_0158
Dickeya_solani_IFB_0221
Dickeya_solani_IPO2019
Dickeya_solani_IPO_2222
Dickeya_solani_M21a
Dickeya_solani_MIE35
Dickeya_solani_MK10
Dickeya_solani_MK16_MK16
Dickeya_solani_PPO9019
Dickeya_solani_PPO9134
Dickeya_solani_RNS10-27-2A
Dickeya_solani_RNS_082331A
Dickeya_solani_Sp1a
Dickeya_solani_RNS08.23.3.1A
1
1
1
1
0.00733405

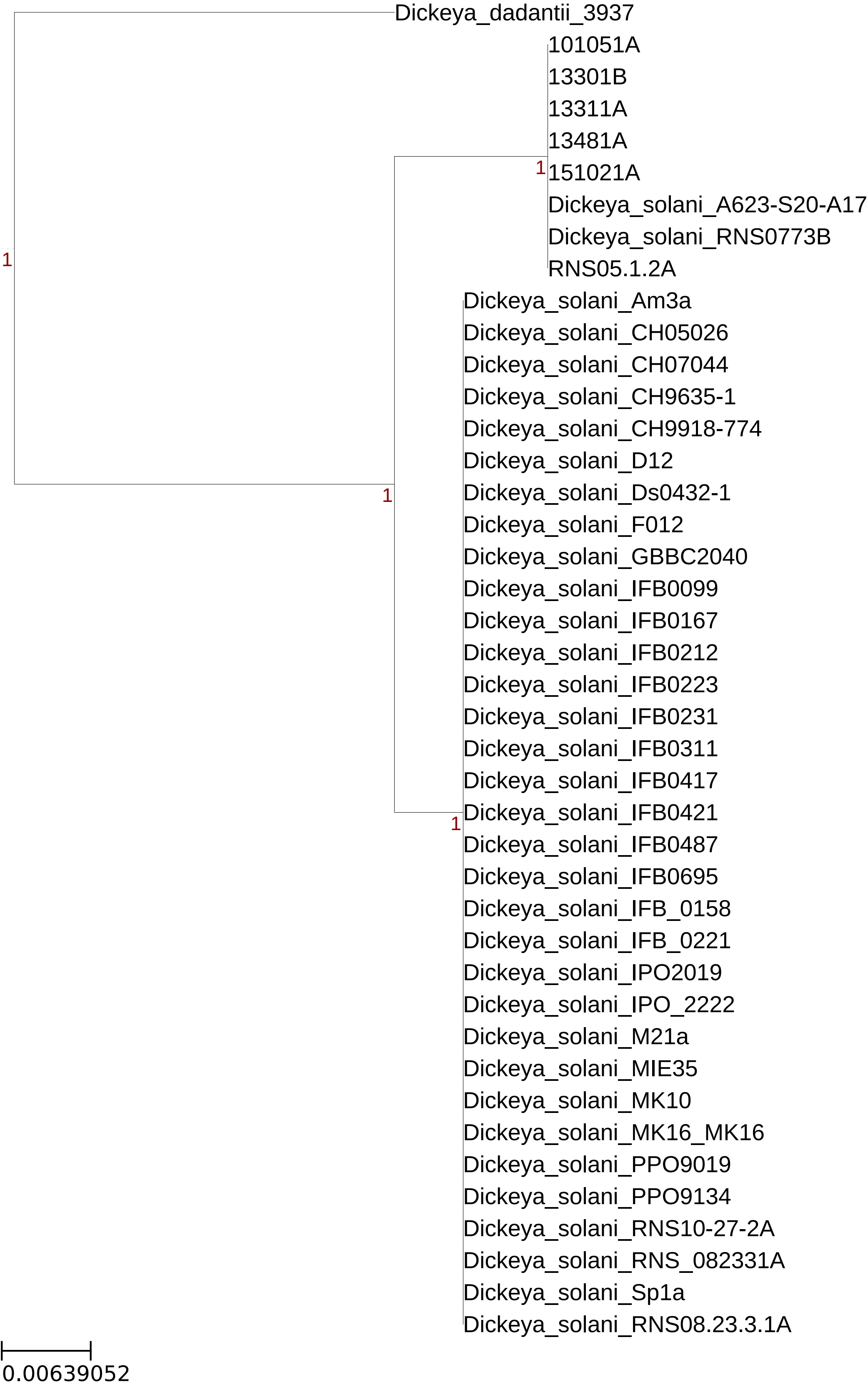
Dickeya_dadantii_3937
101051A
13301B
13311A
13481A
151021A
Dickeya_solani_A623-S20-A17
Dickeya_solani_RNS0773B
RNS05.1.2A
Dickeya_solani_Am3a
Dickeya_solani_CH05026
Dickeya_solani_CH07044
Dickeya_solani_CH9635-1
Dickeya_solani_CH9918-774
Dickeya_solani_D12
Dickeya_solani_Ds0432-1
Dickeya_solani_F012
Dickeya_solani_GBBC2040
Dickeya_solani_IFB0099
Dickeya_solani_IFB0167
Dickeya_solani_IFB0212
Dickeya_solani_IFB0223
Dickeya_solani_IFB0231
Dickeya_solani_IFB0311
Dickeya_solani_IFB0417
Dickeya_solani_IFB0421
Dickeya_solani_IFB0487
Dickeya_solani_IFB0695
Dickeya_solani_IFB_0158
Dickeya_solani_IFB_0221
Dickeya_solani_IPO2019
Dickeya_solani_IPO_2222
Dickeya_solani_M21a
Dickeya_solani_MIE35
Dickeya_solani_MK10
Dickeya_solani_MK16_MK16
Dickeya_solani_PPO9019
Dickeya_solani_PPO9134
Dickeya_solani_RNS10-27-2A
Dickeya_solani_RNS_082331A
Dickeya_solani_Sp1a
Dickeya_solani_RNS08.23.3.1A
1
1
1
1
0.00639052

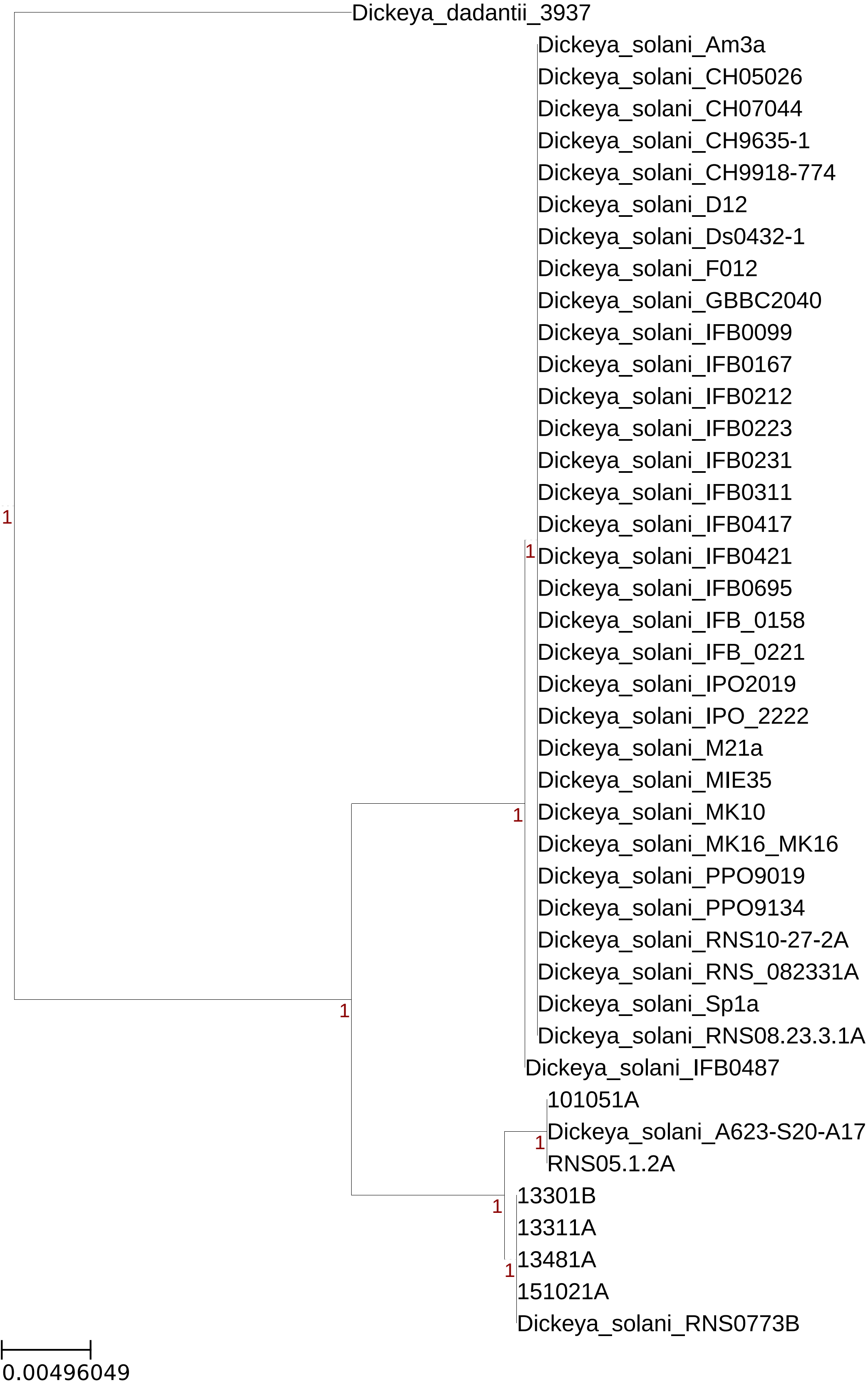
Dickeya_dadantii_3937
Dickeya_solani_Am3a
Dickeya_solani_CH05026
Dickeya_solani_CH07044
Dickeya_solani_CH9635-1
Dickeya_solani_CH9918-774
Dickeya_solani_D12
Dickeya_solani_Ds0432-1
Dickeya_solani_F012
Dickeya_solani_GBBC2040
Dickeya_solani_IFB0099
Dickeya_solani_IFB0167
Dickeya_solani_IFB0212
Dickeya_solani_IFB0223
Dickeya_solani_IFB0231
Dickeya_solani_IFB0311
Dickeya_solani_IFB0417
Dickeya_solani_IFB0421
Dickeya_solani_IFB0695
Dickeya_solani_IFB_0158
Dickeya_solani_IFB_0221
Dickeya_solani_IPO2019
Dickeya_solani_IPO_2222
Dickeya_solani_M21a
Dickeya_solani_MIE35
Dickeya_solani_MK10
Dickeya_solani_MK16_MK16
Dickeya_solani_PPO9019
Dickeya_solani_PPO9134
Dickeya_solani_RNS10-27-2A
Dickeya_solani_RNS_082331A
Dickeya_solani_Sp1a
Dickeya_solani_RNS08.23.3.1A
Dickeya_solani_IFB0487
101051A
Dickeya_solani_A623-S20-A17
RNS05.1.2A
13301B
13311A
13481A
151021A
Dickeya_solani_RNS0773B
1
1
1
1
1
1
1
0.00496049

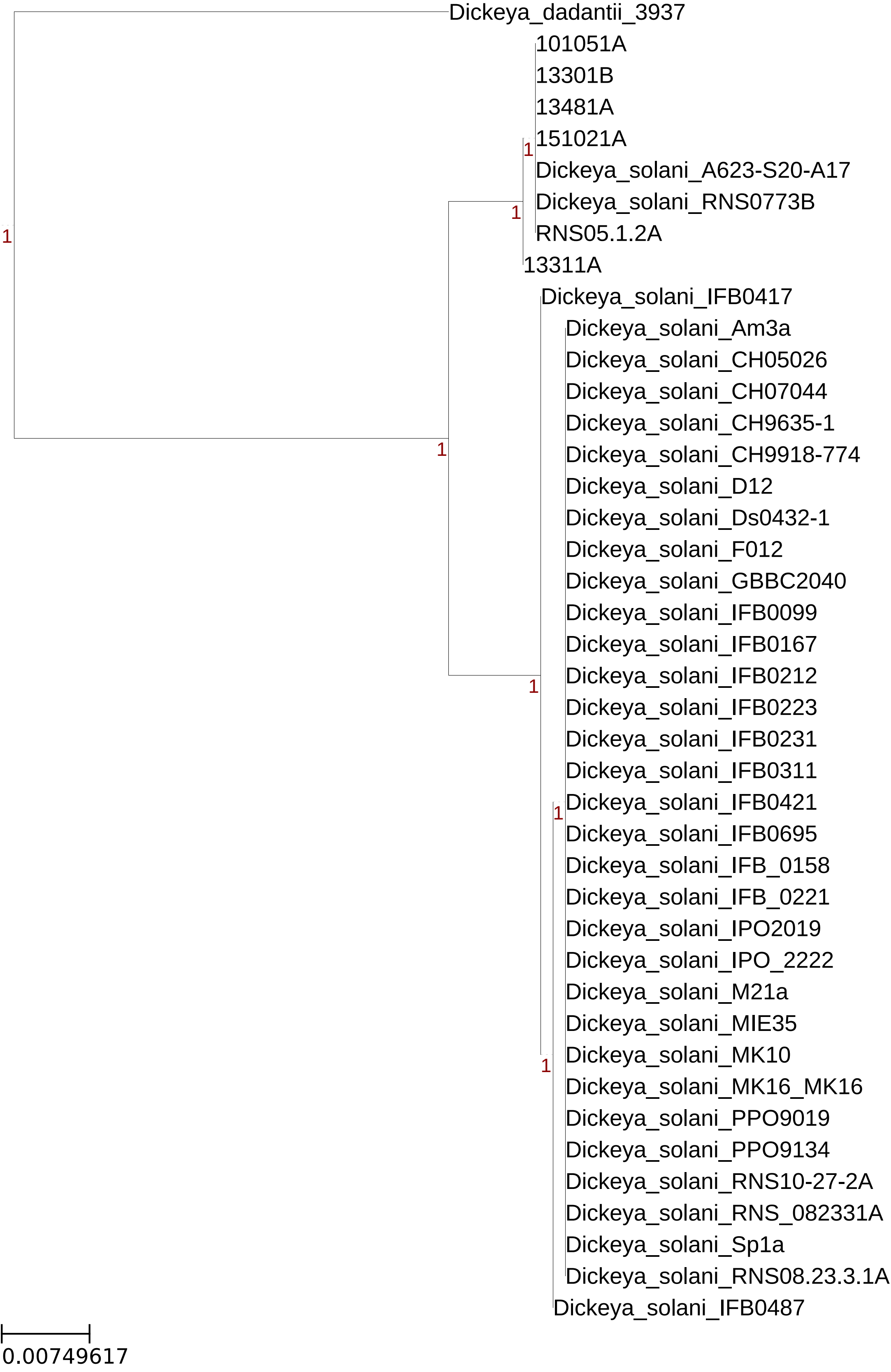
Dickeya_dadantii_3937
101051A
13301B
13481A
151021A
Dickeya_solani_A623-S20-A17
Dickeya_solani_RNS0773B
RNS05.1.2A
13311A
Dickeya_solani_IFB0417
Dickeya_solani_Am3a
Dickeya_solani_CH05026
Dickeya_solani_CH07044
Dickeya_solani_CH9635-1
Dickeya_solani_CH9918-774
Dickeya_solani_D12
Dickeya_solani_Ds0432-1
Dickeya_solani_F012
Dickeya_solani_GBBC2040
Dickeya_solani_IFB0099
Dickeya_solani_IFB0167
Dickeya_solani_IFB0212
Dickeya_solani_IFB0223
Dickeya_solani_IFB0231
Dickeya_solani_IFB0311
Dickeya_solani_IFB0421
Dickeya_solani_IFB0695
Dickeya_solani_IFB_0158
Dickeya_solani_IFB_0221
Dickeya_solani_IPO2019
Dickeya_solani_IPO_2222
Dickeya_solani_M21a
Dickeya_solani_MIE35
Dickeya_solani_MK10
Dickeya_solani_MK16_MK16
Dickeya_solani_PPO9019
Dickeya_solani_PPO9134
Dickeya_solani_RNS10-27-2A
Dickeya_solani_RNS_082331A
Dickeya_solani_Sp1a
Dickeya_solani_RNS08.23.3.1A
Dickeya_solani_IFB0487
1
1
1
1
1
1
1
0.00749617

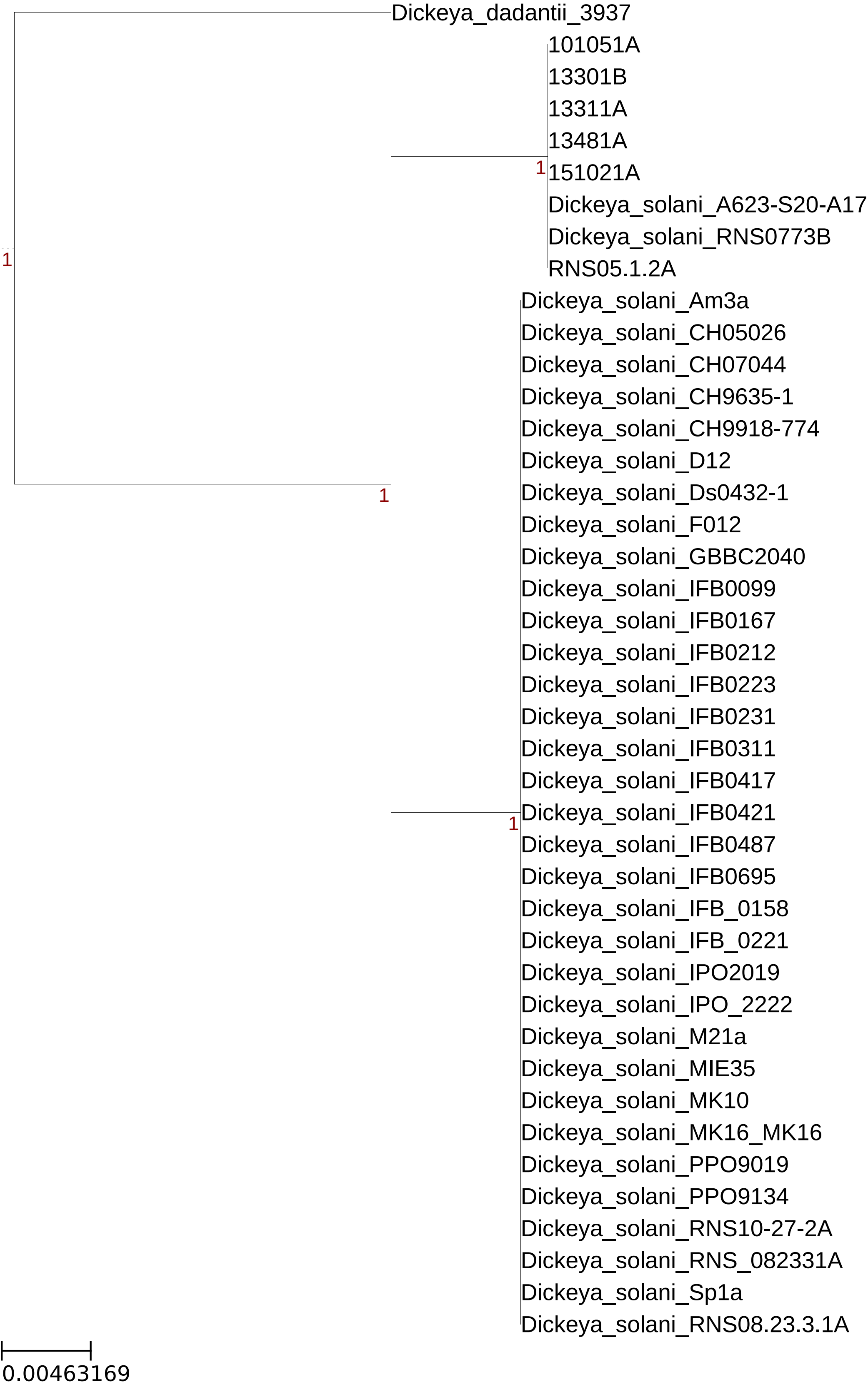
Dickeya_dadantii_3937
101051A
13301B
13311A
13481A
151021A
Dickeya_solani_A623-S20-A17
Dickeya_solani_RNS0773B
RNS05.1.2A
Dickeya_solani_Am3a
Dickeya_solani_CH05026
Dickeya_solani_CH07044
Dickeya_solani_CH9635-1
Dickeya_solani_CH9918-774
Dickeya_solani_D12
Dickeya_solani_Ds0432-1
Dickeya_solani_F012
Dickeya_solani_GBBC2040
Dickeya_solani_IFB0099
Dickeya_solani_IFB0167
Dickeya_solani_IFB0212
Dickeya_solani_IFB0223
Dickeya_solani_IFB0231
Dickeya_solani_IFB0311
Dickeya_solani_IFB0417
Dickeya_solani_IFB0421
Dickeya_solani_IFB0487
Dickeya_solani_IFB0695
Dickeya_solani_IFB_0158
Dickeya_solani_IFB_0221
Dickeya_solani_IPO2019
Dickeya_solani_IPO_2222
Dickeya_solani_M21a
Dickeya_solani_MIE35
Dickeya_solani_MK10
Dickeya_solani_MK16_MK16
Dickeya_solani_PPO9019
Dickeya_solani_PPO9134
Dickeya_solani_RNS10-27-2A
Dickeya_solani_RNS_082331A
Dickeya_solani_Sp1a
Dickeya_solani_RNS08.23.3.1A
1
1
1
1
0.00463169

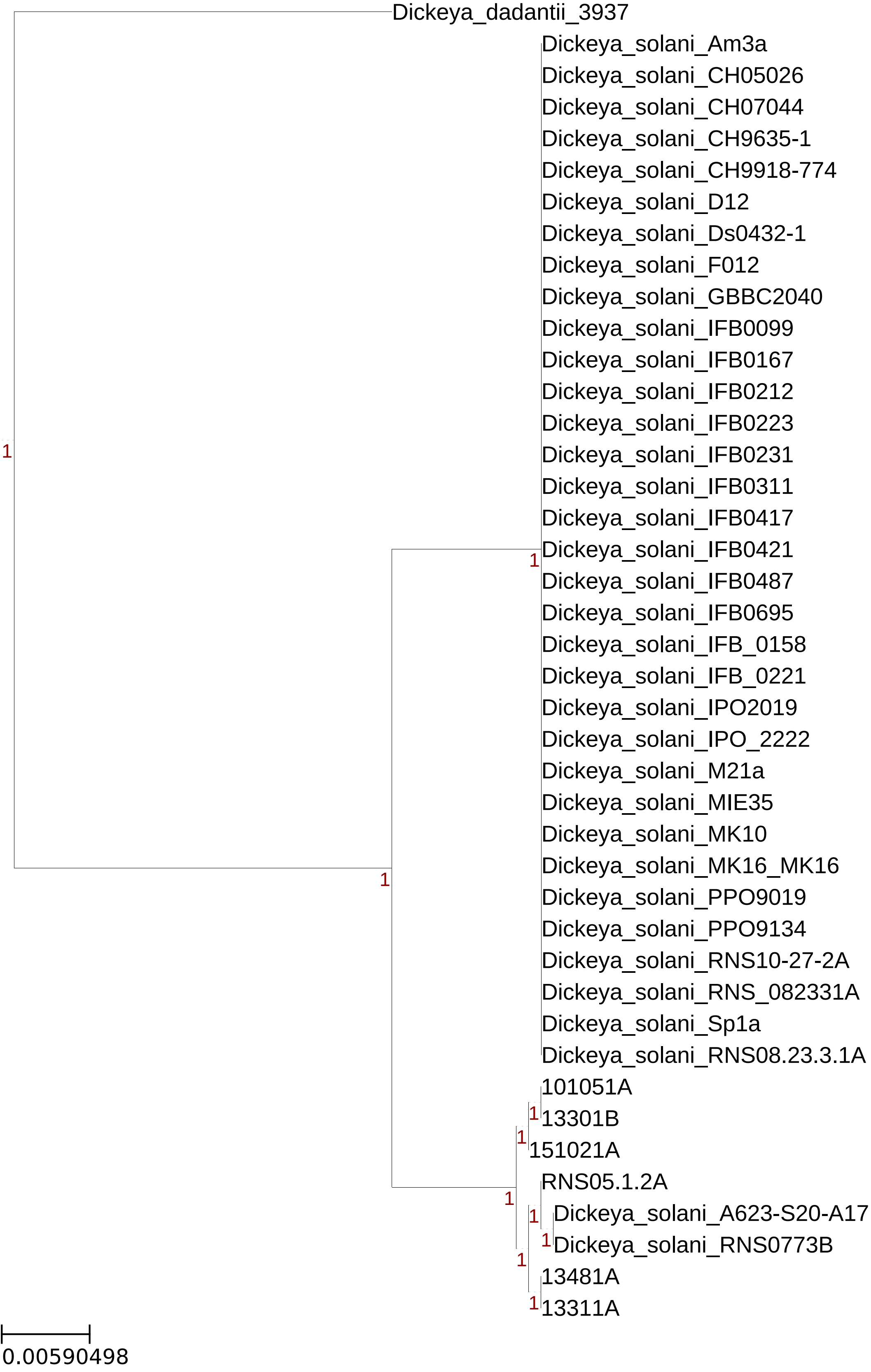
Dickeya_dadantii_3937
Dickeya_solani_Am3a
Dickeya_solani_CH05026
Dickeya_solani_CH07044
Dickeya_solani_CH9635-1
Dickeya_solani_CH9918-774
Dickeya_solani_D12
Dickeya_solani_Ds0432-1
Dickeya_solani_F012
Dickeya_solani_GBBC2040
Dickeya_solani_IFB0099
Dickeya_solani_IFB0167
Dickeya_solani_IFB0212
Dickeya_solani_IFB0223
Dickeya_solani_IFB0231
Dickeya_solani_IFB0311
Dickeya_solani_IFB0417
Dickeya_solani_IFB0421
Dickeya_solani_IFB0487
Dickeya_solani_IFB0695
Dickeya_solani_IFB_0158
Dickeya_solani_IFB_0221
Dickeya_solani_IPO2019
Dickeya_solani_IPO_2222
Dickeya_solani_M21a
Dickeya_solani_MIE35
Dickeya_solani_MK10
Dickeya_solani_MK16_MK16
Dickeya_solani_PPO9019
Dickeya_solani_PPO9134
Dickeya_solani_RNS10-27-2A
Dickeya_solani_RNS_082331A
Dickeya_solani_Sp1a
Dickeya_solani_RNS08.23.3.1A
101051A
13301B
151021A
RNS05.1.2A
Dickeya_solani_A623-S20-A17
Dickeya_solani_RNS0773B
13481A
13311A
0.00590498

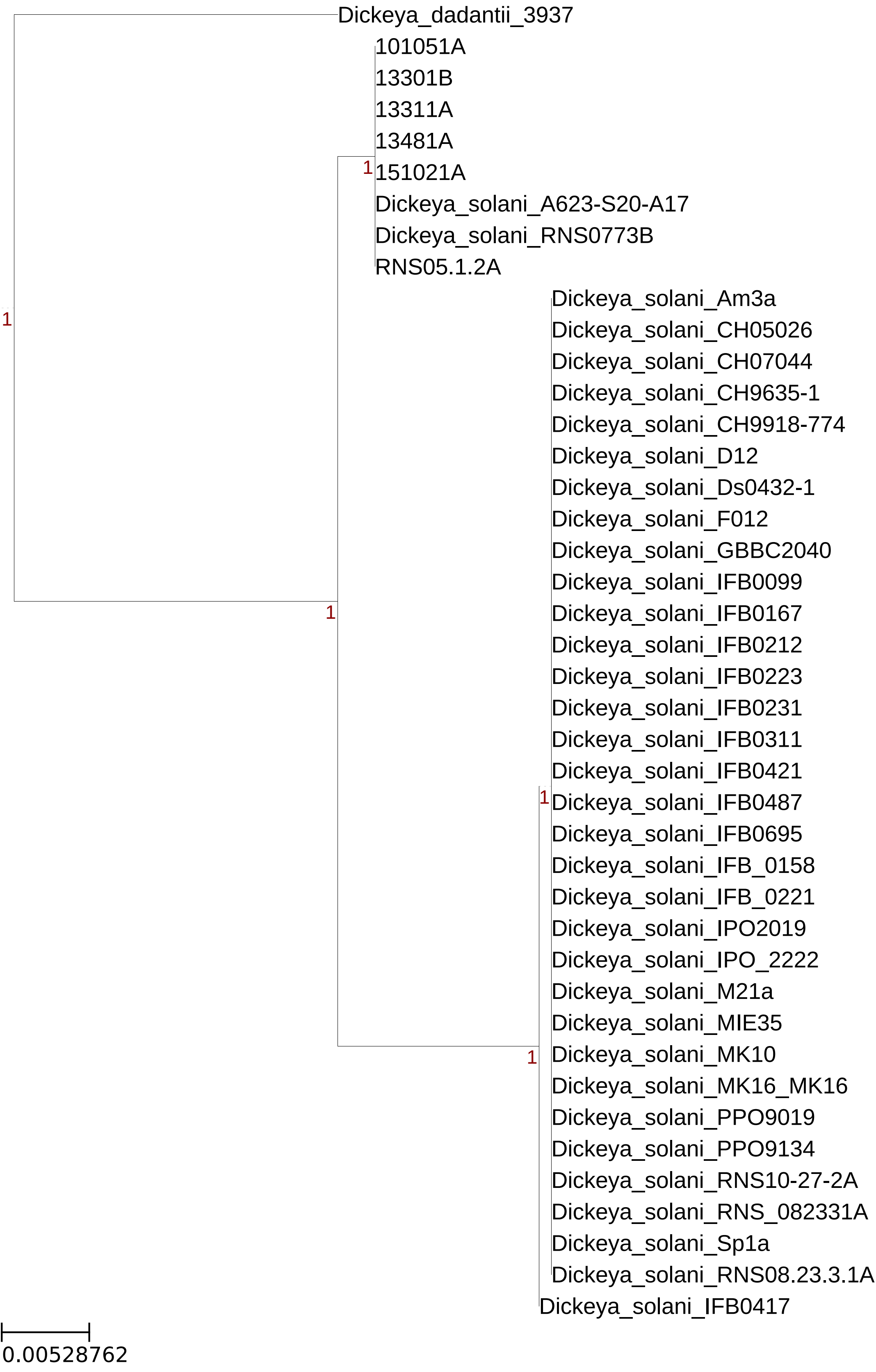

Dickeya_dadantii_3937
101051A
13301B
13311A
13481A
151021A
Dickeya_solani_A623-S20-A17
Dickeya_solani_RNS0773B
RNS05.1.2A
Dickeya_solani_Am3a
Dickeya_solani_CH05026
Dickeya_solani_CH07044
Dickeya_solani_CH9635-1
Dickeya_solani_CH9918-774
Dickeya_solani_D12
Dickeya_solani_Ds0432-1
Dickeya_solani_F012
Dickeya_solani_GBBC2040
Dickeya_solani_IFB0099
Dickeya_solani_IFB0167
Dickeya_solani_IFB0212
Dickeya_solani_IFB0223
Dickeya_solani_IFB0231
Dickeya_solani_IFB0311
Dickeya_solani_IFB0421
Dickeya_solani_IFB0487
Dickeya_solani_IFB0695
Dickeya_solani_IFB_0158
Dickeya_solani_IFB_0221
Dickeya_solani_IPO2019
Dickeya_solani_IPO_2222
Dickeya_solani_M21a
Dickeya_solani_MIE35
Dickeya_solani_MK10
Dickeya_solani_MK16_MK16
Dickeya_solani_PPO9019
Dickeya_solani_PPO9134
Dickeya_solani_RNS10-27-2A
Dickeya_solani_RNS_082331A
Dickeya_solani_Sp1a
Dickeya_solani_RNS08.23.3.1A
Dickeya_solani_IFB0417
1
1
1
1
1
0.00528762

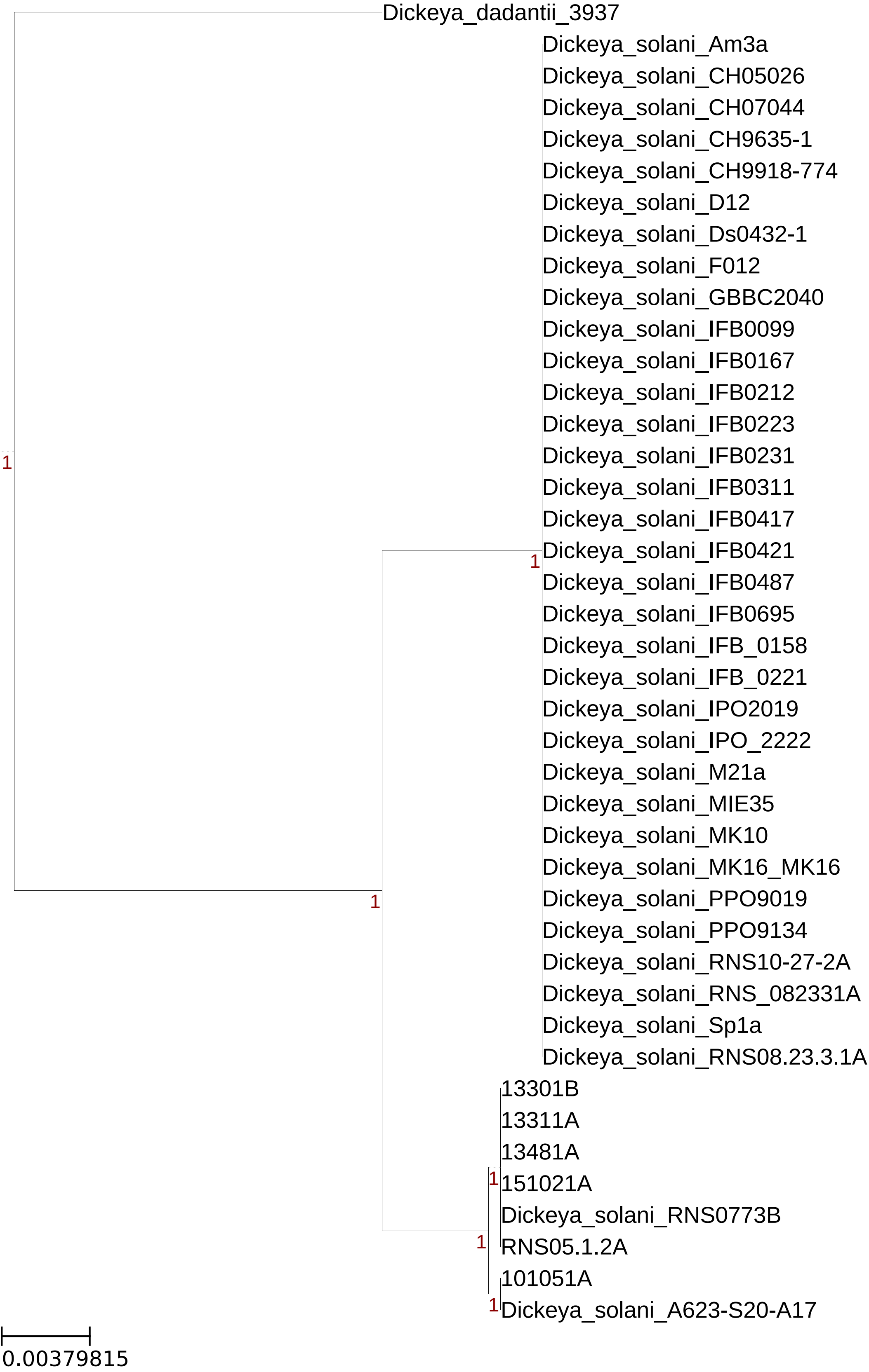
Dickeya_dadantii_3937
Dickeya_solani_Am3a
Dickeya_solani_CH05026
Dickeya_solani_CH07044
Dickeya_solani_CH9635-1
Dickeya_solani_CH9918-774
Dickeya_solani_D12
Dickeya_solani_Ds0432-1
Dickeya_solani_F012
Dickeya_solani_GBBC2040
Dickeya_solani_IFB0099
Dickeya_solani_IFB0167
Dickeya_solani_IFB0212
Dickeya_solani_IFB0223
Dickeya_solani_IFB0231
Dickeya_solani_IFB0311
Dickeya_solani_IFB0417
Dickeya_solani_IFB0421
Dickeya_solani_IFB0487
Dickeya_solani_IFB0695
Dickeya_solani_IFB_0158
Dickeya_solani_IFB_0221
Dickeya_solani_IPO2019
Dickeya_solani_IPO_2222
Dickeya_solani_M21a
Dickeya_solani_MIE35
Dickeya_solani_MK10
Dickeya_solani_MK16_MK16
Dickeya_solani_PPO9019
Dickeya_solani_PPO9134
Dickeya_solani_RNS10-27-2A
Dickeya_solani_RNS_082331A
Dickeya_solani_Sp1a
Dickeya_solani_RNS08.23.3.1A
13301B
13311A
13481A
151021A
Dickeya_solani_RNS0773B
RNS05.1.2A
101051A
Dickeya_solani_A623-S20-A17
1
1
1
1
1
1
0.00379815

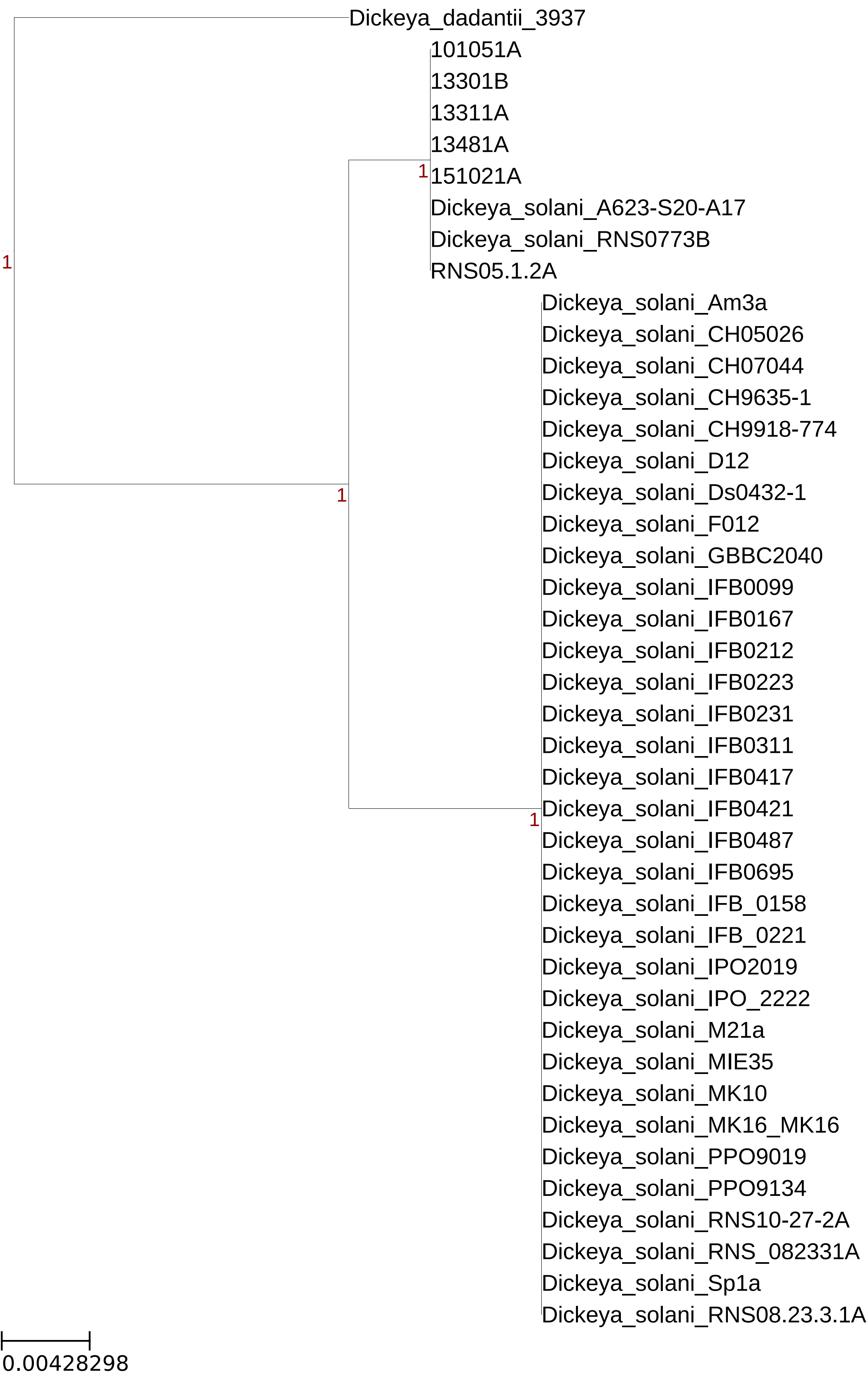
Dickeya_dadantii_3937
101051A
13301B
13311A
13481A
151021A
Dickeya_solani_A623-S20-A17
Dickeya_solani_RNS0773B
RNS05.1.2A
Dickeya_solani_Am3a
Dickeya_solani_CH05026
Dickeya_solani_CH07044
Dickeya_solani_CH9635-1
Dickeya_solani_CH9918-774
Dickeya_solani_D12
Dickeya_solani_Ds0432-1
Dickeya_solani_F012
Dickeya_solani_GBBC2040
Dickeya_solani_IFB0099
Dickeya_solani_IFB0167
Dickeya_solani_IFB0212
Dickeya_solani_IFB0223
Dickeya_solani_IFB0231
Dickeya_solani_IFB0311
Dickeya_solani_IFB0417
Dickeya_solani_IFB0421
Dickeya_solani_IFB0487
Dickeya_solani_IFB0695
Dickeya_solani_IFB_0158
Dickeya_solani_IFB_0221
Dickeya_solani_IPO2019
Dickeya_solani_IPO_2222
Dickeya_solani_M21a
Dickeya_solani_MIE35
Dickeya_solani_MK10
Dickeya_solani_MK16_MK16
Dickeya_solani_PPO9019
Dickeya_solani_PPO9134
Dickeya_solani_RNS10-27-2A
Dickeya_solani_RNS_082331A
Dickeya_solani_Sp1a
Dickeya_solani_RNS08.23.3.1A
1
1
1
1
0.00428298

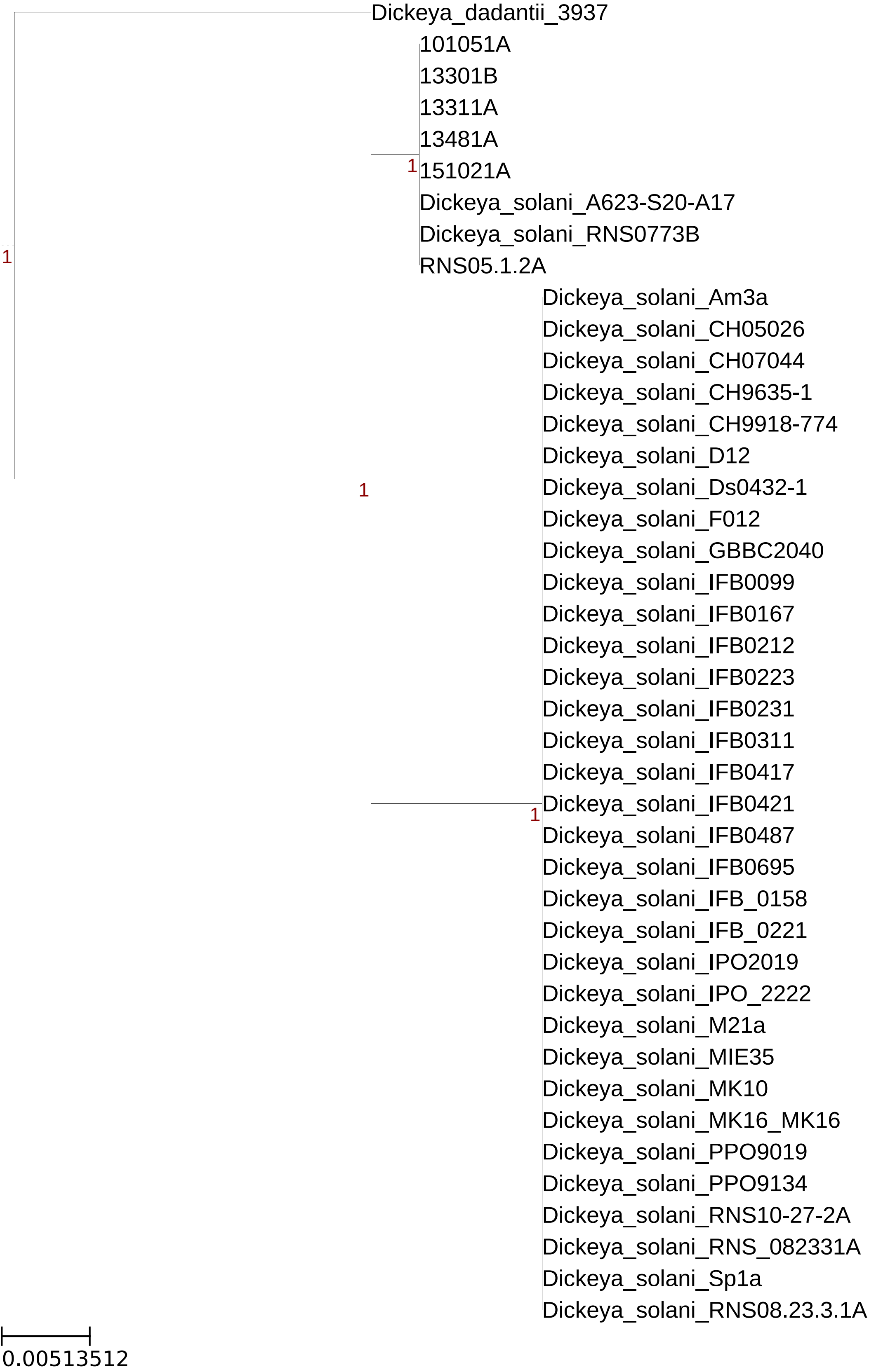

Dickeya_dadantii_3937
101051A
13301B
13311A
13481A
151021A
Dickeya_solani_A623-S20-A17
Dickeya_solani_RNS0773B
RNS05.1.2A
Dickeya_solani_Am3a
Dickeya_solani_CH05026
Dickeya_solani_CH07044
Dickeya_solani_CH9635-1
Dickeya_solani_CH9918-774
Dickeya_solani_D12
Dickeya_solani_Ds0432-1
Dickeya_solani_F012
Dickeya_solani_GBBC2040
Dickeya_solani_IFB0099
Dickeya_solani_IFB0167
Dickeya_solani_IFB0212
Dickeya_solani_IFB0223
Dickeya_solani_IFB0231
Dickeya_solani_IFB0311
Dickeya_solani_IFB0417
Dickeya_solani_IFB0421
Dickeya_solani_IFB0487
Dickeya_solani_IFB0695
Dickeya_solani_IFB_0158
Dickeya_solani_IFB_0221
Dickeya_solani_IPO2019
Dickeya_solani_IPO_2222
Dickeya_solani_M21a
Dickeya_solani_MIE35
Dickeya_solani_MK10
Dickeya_solani_MK16_MK16
Dickeya_solani_PPO9019
Dickeya_solani_PPO9134
Dickeya_solani_RNS10-27-2A
Dickeya_solani_RNS_082331A
Dickeya_solani_Sp1a
Dickeya_solani_RNS08.23.3.1A
1
1
1
1
0.00513512

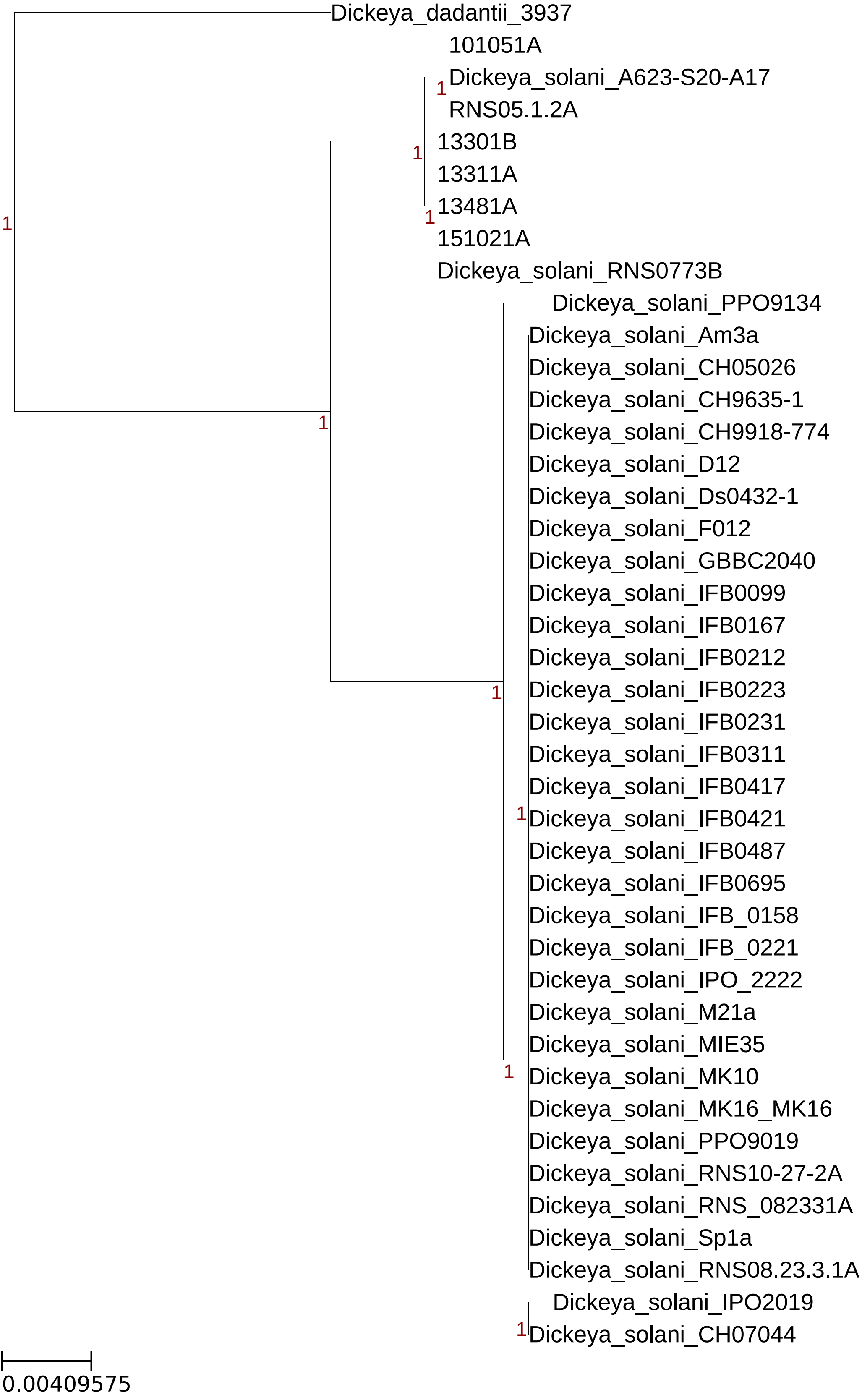
Dickeya_dadantii_3937
101051A
Dickeya_solani_A623-S20-A17
RNS05.1.2A
13301B
13311A
13481A
151021A
Dickeya_solani_RNS0773B
Dickeya_solani_PPO9134
Dickeya_solani_Am3a
Dickeya_solani_CH05026
Dickeya_solani_CH9635-1
Dickeya_solani_CH9918-774
Dickeya_solani_D12
Dickeya_solani_Ds0432-1
Dickeya_solani_F012
Dickeya_solani_GBBC2040
Dickeya_solani_IFB0099
Dickeya_solani_IFB0167
Dickeya_solani_IFB0212
Dickeya_solani_IFB0223
Dickeya_solani_IFB0231
Dickeya_solani_IFB0311
Dickeya_solani_IFB0417
Dickeya_solani_IFB0421
Dickeya_solani_IFB0487
Dickeya_solani_IFB0695
Dickeya_solani_IFB_0158
Dickeya_solani_IFB_0221
Dickeya_solani_IPO_2222
Dickeya_solani_M21a
Dickeya_solani_MIE35
Dickeya_solani_MK10
Dickeya_solani_MK16_MK16
Dickeya_solani_PPO9019
Dickeya_solani_RNS10-27-2A
Dickeya_solani_RNS_082331A
Dickeya_solani_Sp1a
Dickeya_solani_RNS08.23.3.1A
Dickeya_solani_IPO2019
Dickeya_solani_CH07044
1
0.00409575

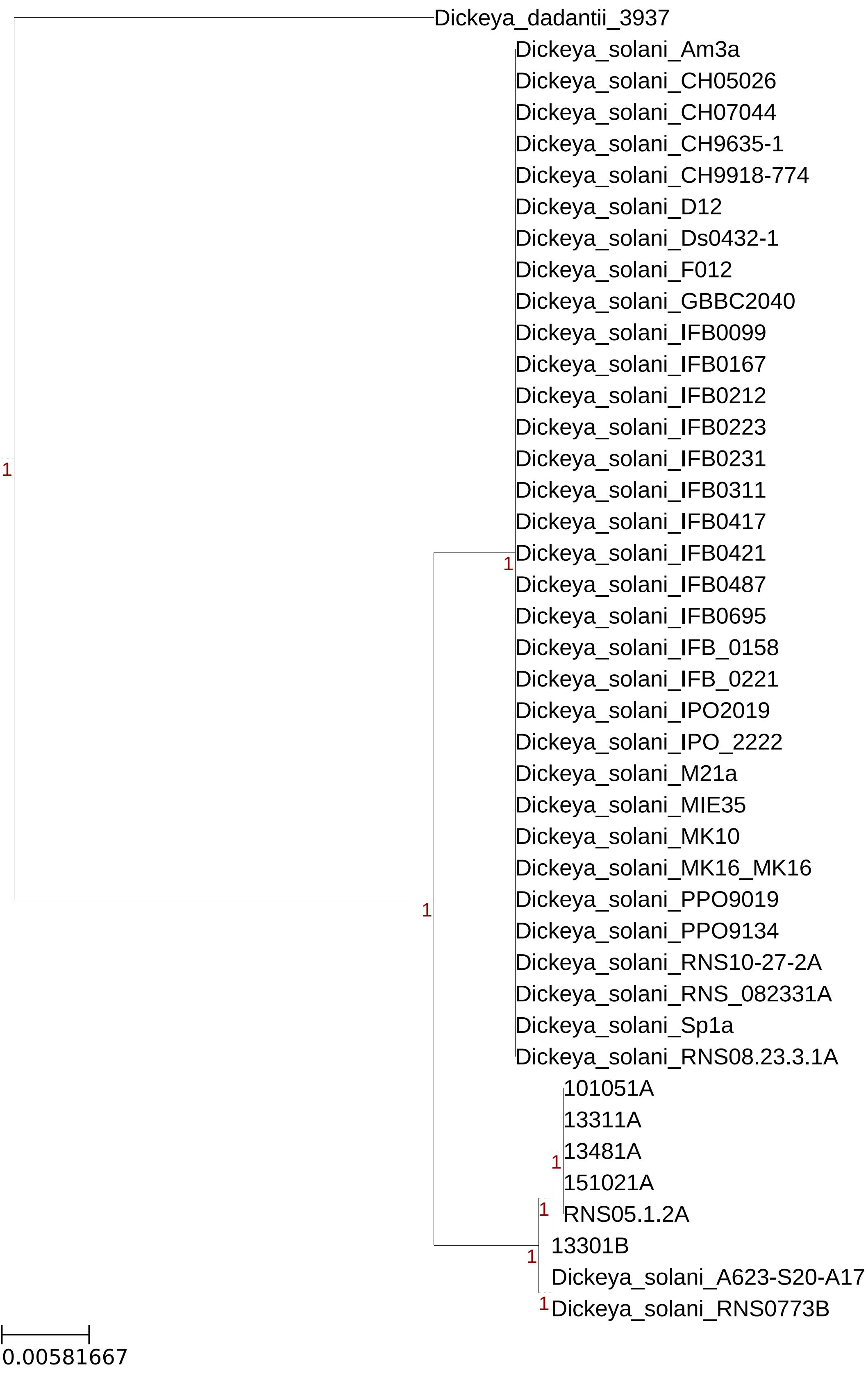
Dickeya_dadantii_3937
Dickeya_solani_Am3a
Dickeya_solani_CH05026
Dickeya_solani_CH07044
Dickeya_solani_CH9635-1
Dickeya_solani_CH9918-774
Dickeya_solani_D12
Dickeya_solani_Ds0432-1
Dickeya_solani_F012
Dickeya_solani_GBBC2040
Dickeya_solani_IFB0099
Dickeya_solani_IFB0167
Dickeya_solani_IFB0212
Dickeya_solani_IFB0223
Dickeya_solani_IFB0231
Dickeya_solani_IFB0311
Dickeya_solani_IFB0417
Dickeya_solani_IFB0421
Dickeya_solani_IFB0487
Dickeya_solani_IFB0695
Dickeya_solani_IFB_0158
Dickeya_solani_IFB_0221
Dickeya_solani_IPO2019
Dickeya_solani_IPO_2222
Dickeya_solani_M21a
Dickeya_solani_MIE35
Dickeya_solani_MK10
Dickeya_solani_MK16_MK16
Dickeya_solani_PPO9019
Dickeya_solani_PPO9134
Dickeya_solani_RNS10-27-2A
Dickeya_solani_RNS_082331A
Dickeya_solani_Sp1a
Dickeya_solani_RNS08.23.3.1A
101051A
13311A
13481A
151021A
RNS05.1.2A
13301B
Dickeya_solani_A623-S20-A17
Dickeya_solani_RNS0773B
1
1
1
1
1
1
1
0.00581667

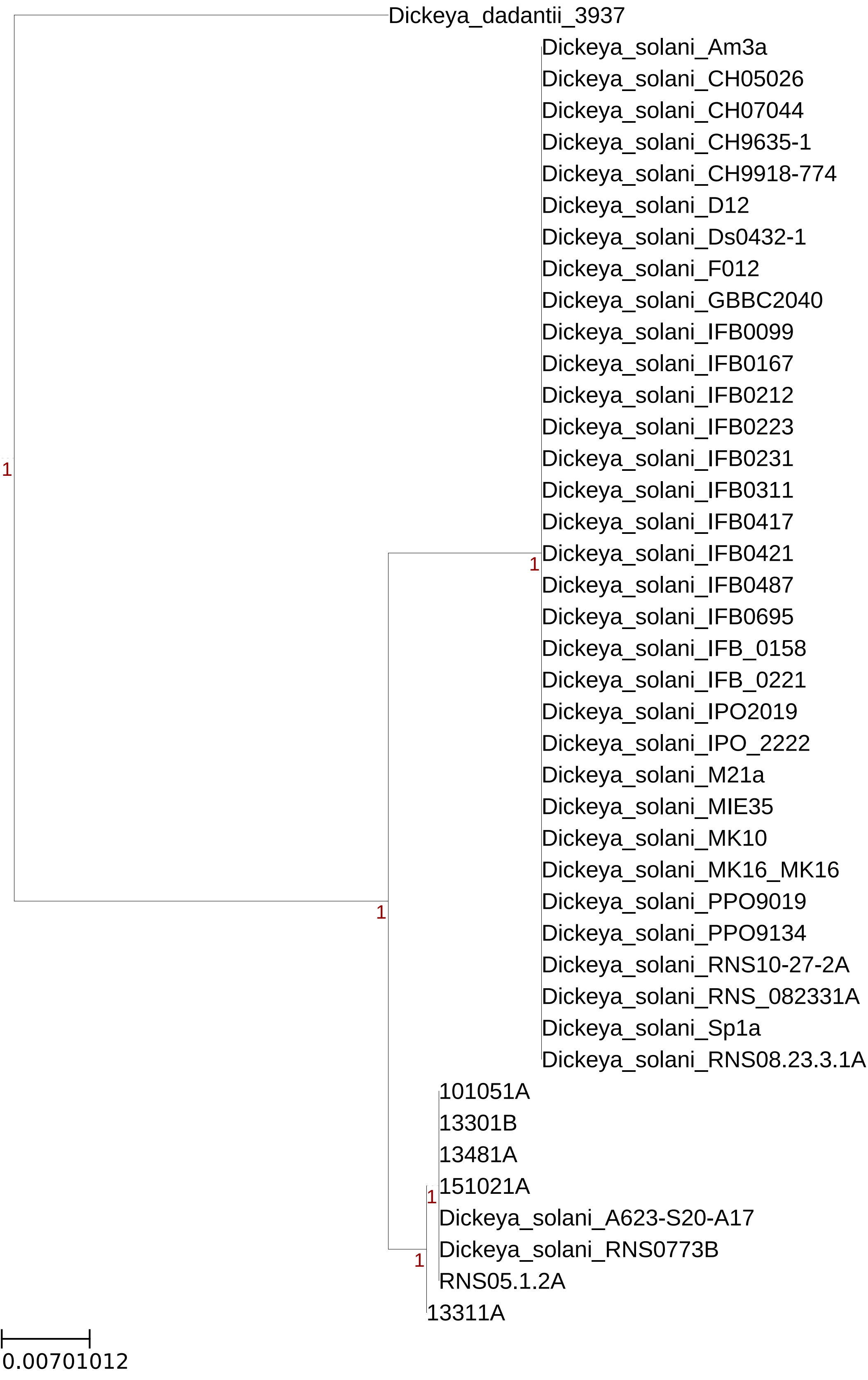
Dickeya_dadantii_3937
Dickeya_solani_Am3a
Dickeya_solani_CH05026
Dickeya_solani_CH07044
Dickeya_solani_CH9635-1
Dickeya_solani_CH9918-774
Dickeya_solani_D12
Dickeya_solani_Ds0432-1
Dickeya_solani_F012
Dickeya_solani_GBBC2040
Dickeya_solani_IFB0099
Dickeya_solani_IFB0167
Dickeya_solani_IFB0212
Dickeya_solani_IFB0223
Dickeya_solani_IFB0231
Dickeya_solani_IFB0311
Dickeya_solani_IFB0417
Dickeya_solani_IFB0421
Dickeya_solani_IFB0487
Dickeya_solani_IFB0695
Dickeya_solani_IFB_0158
Dickeya_solani_IFB_0221
Dickeya_solani_IPO2019
Dickeya_solani_IPO_2222
Dickeya_solani_M21a
Dickeya_solani_MIE35
Dickeya_solani_MK10
Dickeya_solani_MK16_MK16
Dickeya_solani_PPO9019
Dickeya_solani_PPO9134
Dickeya_solani_RNS10-27-2A
Dickeya_solani_RNS_082331A
Dickeya_solani_Sp1a
Dickeya_solani_RNS08.23.3.1A
101051A
13301B
13481A
151021A
Dickeya_solani_A623-S20-A17
Dickeya_solani_RNS0773B
RNS05.1.2A
13311A
1
1
1
1
1
0.00701012

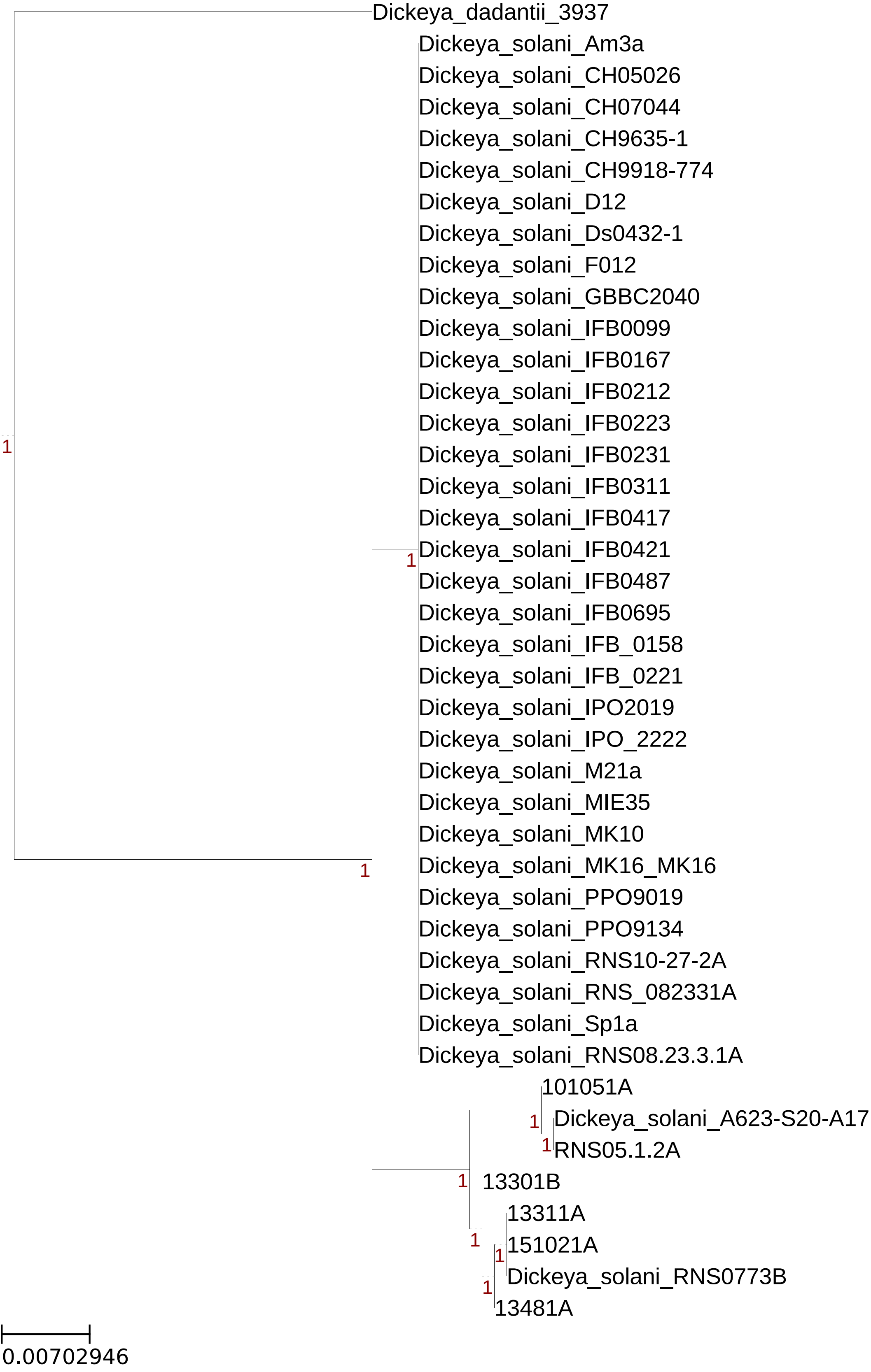
Dickeya_dadantii_3937
Dickeya_solani_Am3a
Dickeya_solani_CH05026
Dickeya_solani_CH07044
Dickeya_solani_CH9635-1
Dickeya_solani_CH9918-774
Dickeya_solani_D12
Dickeya_solani_Ds0432-1
Dickeya_solani_F012
Dickeya_solani_GBBC2040
Dickeya_solani_IFB0099
Dickeya_solani_IFB0167
Dickeya_solani_IFB0212
Dickeya_solani_IFB0223
Dickeya_solani_IFB0231
Dickeya_solani_IFB0311
Dickeya_solani_IFB0417
Dickeya_solani_IFB0421
Dickeya_solani_IFB0487
Dickeya_solani_IFB0695
Dickeya_solani_IFB_0158
Dickeya_solani_IFB_0221
Dickeya_solani_IPO2019
Dickeya_solani_IPO_2222
Dickeya_solani_M21a
Dickeya_solani_MIE35
Dickeya_solani_MK10
Dickeya_solani_MK16_MK16
Dickeya_solani_PPO9019
Dickeya_solani_PPO9134
Dickeya_solani_RNS10-27-2A
Dickeya_solani_RNS_082331A
Dickeya_solani_Sp1a
Dickeya_solani_RNS08.23.3.1A
101051A
Dickeya_solani_A623-S20-A17
RNS05.1.2A
13301B
13311A
151021A
Dickeya_solani_RNS0773B
13481A
1
1
1
1
1
1
1
1
1
0.00702946

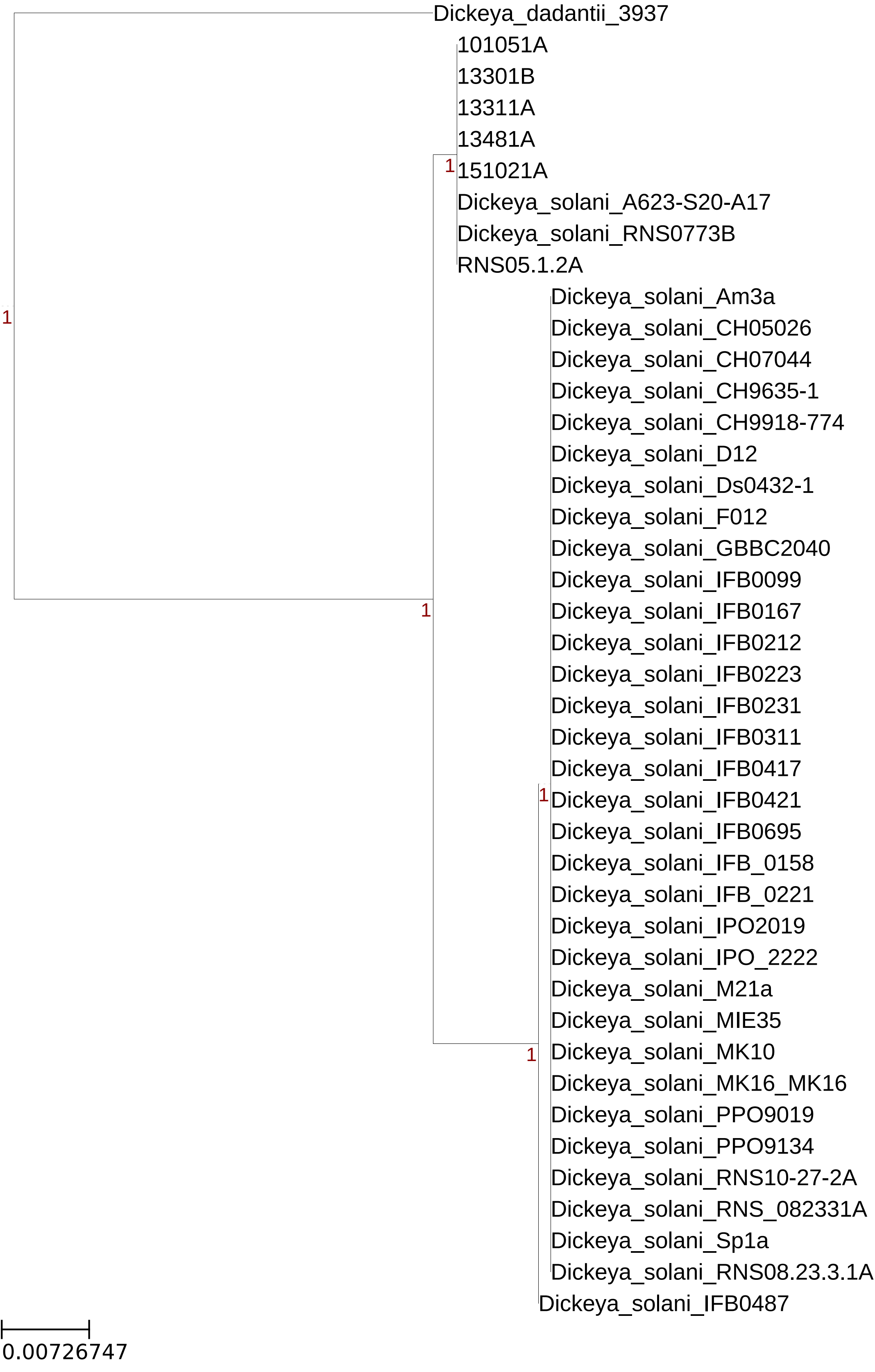
Dickeya_dadantii_3937
101051A
13301B
13311A
13481A
151021A
Dickeya_solani_A623-S20-A17
Dickeya_solani_RNS0773B
RNS05.1.2A
Dickeya_solani_Am3a
Dickeya_solani_CH05026
Dickeya_solani_CH07044
Dickeya_solani_CH9635-1
Dickeya_solani_CH9918-774
Dickeya_solani_D12
Dickeya_solani_Ds0432-1
Dickeya_solani_F012
Dickeya_solani_GBBC2040
Dickeya_solani_IFB0099
Dickeya_solani_IFB0167
Dickeya_solani_IFB0212
Dickeya_solani_IFB0223
Dickeya_solani_IFB0231
Dickeya_solani_IFB0311
Dickeya_solani_IFB0417
Dickeya_solani_IFB0421
Dickeya_solani_IFB0695
Dickeya_solani_IFB_0158
Dickeya_solani_IFB_0221
Dickeya_solani_IPO2019
Dickeya_solani_IPO_2222
Dickeya_solani_M21a
Dickeya_solani_MIE35
Dickeya_solani_MK10
Dickeya_solani_MK16_MK16
Dickeya_solani_PPO9019
Dickeya_solani_PPO9134
Dickeya_solani_RNS10-27-2A
Dickeya_solani_RNS_082331A
Dickeya_solani_Sp1a
Dickeya_solani_RNS08.23.3.1A
Dickeya_solani_IFB0487
1
1
1
1
1
0.00726747

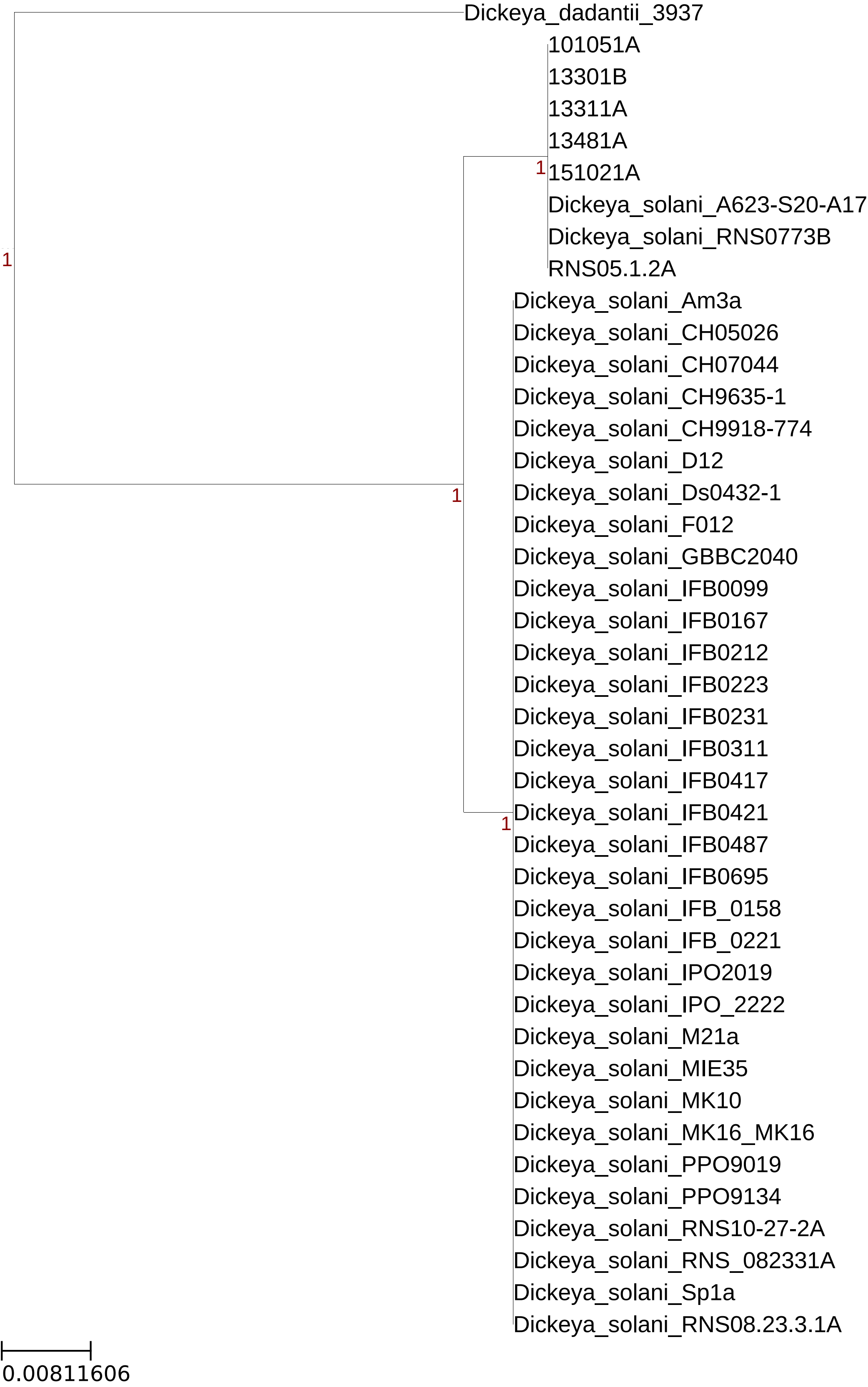
Dickeya_dadantii_3937
101051A
13301B
13311A
13481A
151021A
Dickeya_solani_A623-S20-A17
Dickeya_solani_RNS0773B
RNS05.1.2A
Dickeya_solani_Am3a
Dickeya_solani_CH05026
Dickeya_solani_CH07044
Dickeya_solani_CH9635-1
Dickeya_solani_CH9918-774
Dickeya_solani_D12
Dickeya_solani_Ds0432-1
Dickeya_solani_F012
Dickeya_solani_GBBC2040
Dickeya_solani_IFB0099
Dickeya_solani_IFB0167
Dickeya_solani_IFB0212
Dickeya_solani_IFB0223
Dickeya_solani_IFB0231
Dickeya_solani_IFB0311
Dickeya_solani_IFB0417
Dickeya_solani_IFB0421
Dickeya_solani_IFB0487
Dickeya_solani_IFB0695
Dickeya_solani_IFB_0158
Dickeya_solani_IFB_0221
Dickeya_solani_IPO2019
Dickeya_solani_IPO_2222
Dickeya_solani_M21a
Dickeya_solani_MIE35
Dickeya_solani_MK10
Dickeya_solani_MK16_MK16
Dickeya_solani_PPO9019
Dickeya_solani_PPO9134
Dickeya_solani_RNS10-27-2A
Dickeya_solani_RNS_082331A
Dickeya_solani_Sp1a
Dickeya_solani_RNS08.23.3.1A
1
1
1
1
0.00811606

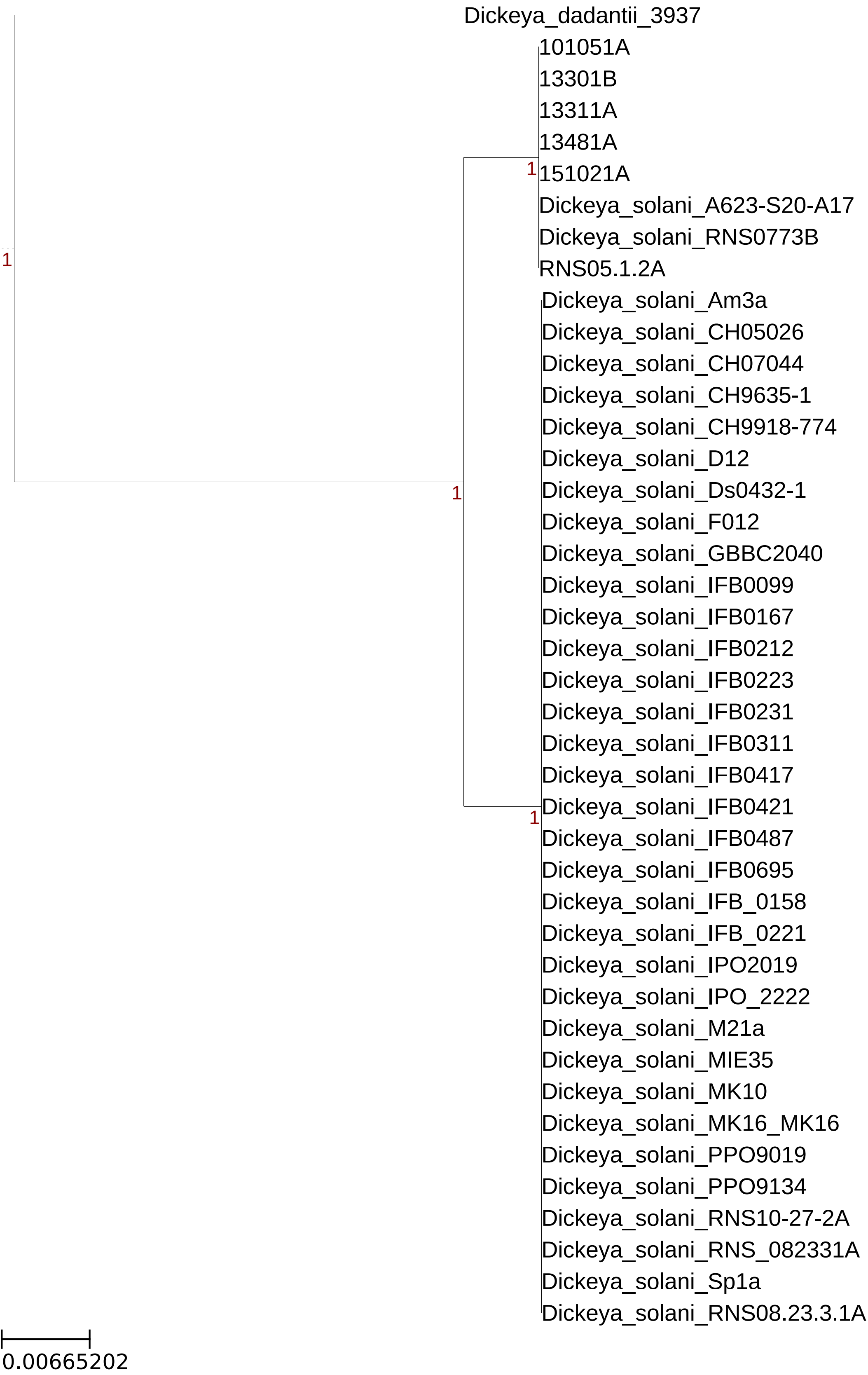
Dickeya_dadantii_3937
101051A
13301B
13311A
13481A
151021A
Dickeya_solani_A623-S20-A17
Dickeya_solani_RNS0773B
RNS05.1.2A
Dickeya_solani_Am3a
Dickeya_solani_CH05026
Dickeya_solani_CH07044
Dickeya_solani_CH9635-1
Dickeya_solani_CH9918-774
Dickeya_solani_D12
Dickeya_solani_Ds0432-1
Dickeya_solani_F012
Dickeya_solani_GBBC2040
Dickeya_solani_IFB0099
Dickeya_solani_IFB0167
Dickeya_solani_IFB0212
Dickeya_solani_IFB0223
Dickeya_solani_IFB0231
Dickeya_solani_IFB0311
Dickeya_solani_IFB0417
Dickeya_solani_IFB0421
Dickeya_solani_IFB0487
Dickeya_solani_IFB0695
Dickeya_solani_IFB_0158
Dickeya_solani_IFB_0221
Dickeya_solani_IPO2019
Dickeya_solani_IPO_2222
Dickeya_solani_M21a
Dickeya_solani_MIE35
Dickeya_solani_MK10
Dickeya_solani_MK16_MK16
Dickeya_solani_PPO9019
Dickeya_solani_PPO9134
Dickeya_solani_RNS10-27-2A
Dickeya_solani_RNS_082331A
Dickeya_solani_Sp1a
Dickeya_solani_RNS08.23.3.1A
1
1
1
1
0.00665202

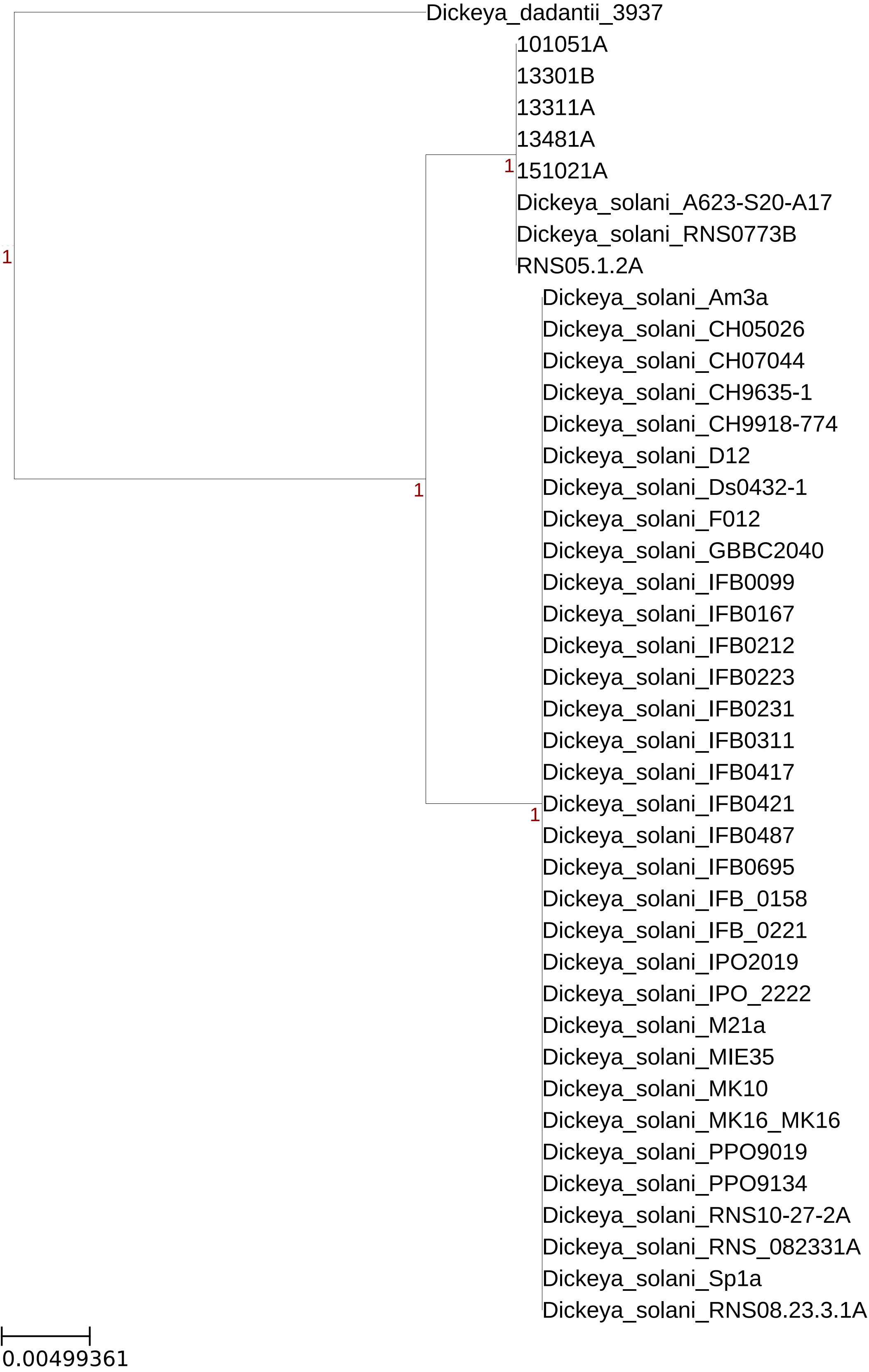
Dickeya_dadantii_3937
101051A
13301B
13311A
13481A
151021A
Dickeya_solani_A623-S20-A17
Dickeya_solani_RNS0773B
RNS05.1.2A
Dickeya_solani_Am3a
Dickeya_solani_CH05026
Dickeya_solani_CH07044
Dickeya_solani_CH9635-1
Dickeya_solani_CH9918-774
Dickeya_solani_D12
Dickeya_solani_Ds0432-1
Dickeya_solani_F012
Dickeya_solani_GBBC2040
Dickeya_solani_IFB0099
Dickeya_solani_IFB0167
Dickeya_solani_IFB0212
Dickeya_solani_IFB0223
Dickeya_solani_IFB0231
Dickeya_solani_IFB0311
Dickeya_solani_IFB0417
Dickeya_solani_IFB0421
Dickeya_solani_IFB0487
Dickeya_solani_IFB0695
Dickeya_solani_IFB_0158
Dickeya_solani_IFB_0221
Dickeya_solani_IPO2019
Dickeya_solani_IPO_2222
Dickeya_solani_M21a
Dickeya_solani_MIE35
Dickeya_solani_MK10
Dickeya_solani_MK16_MK16
Dickeya_solani_PPO9019
Dickeya_solani_PPO9134
Dickeya_solani_RNS10-27-2A
Dickeya_solani_RNS_082331A
Dickeya_solani_Sp1a
Dickeya_solani_RNS08.23.3.1A
1
1
1
1
0.00499361

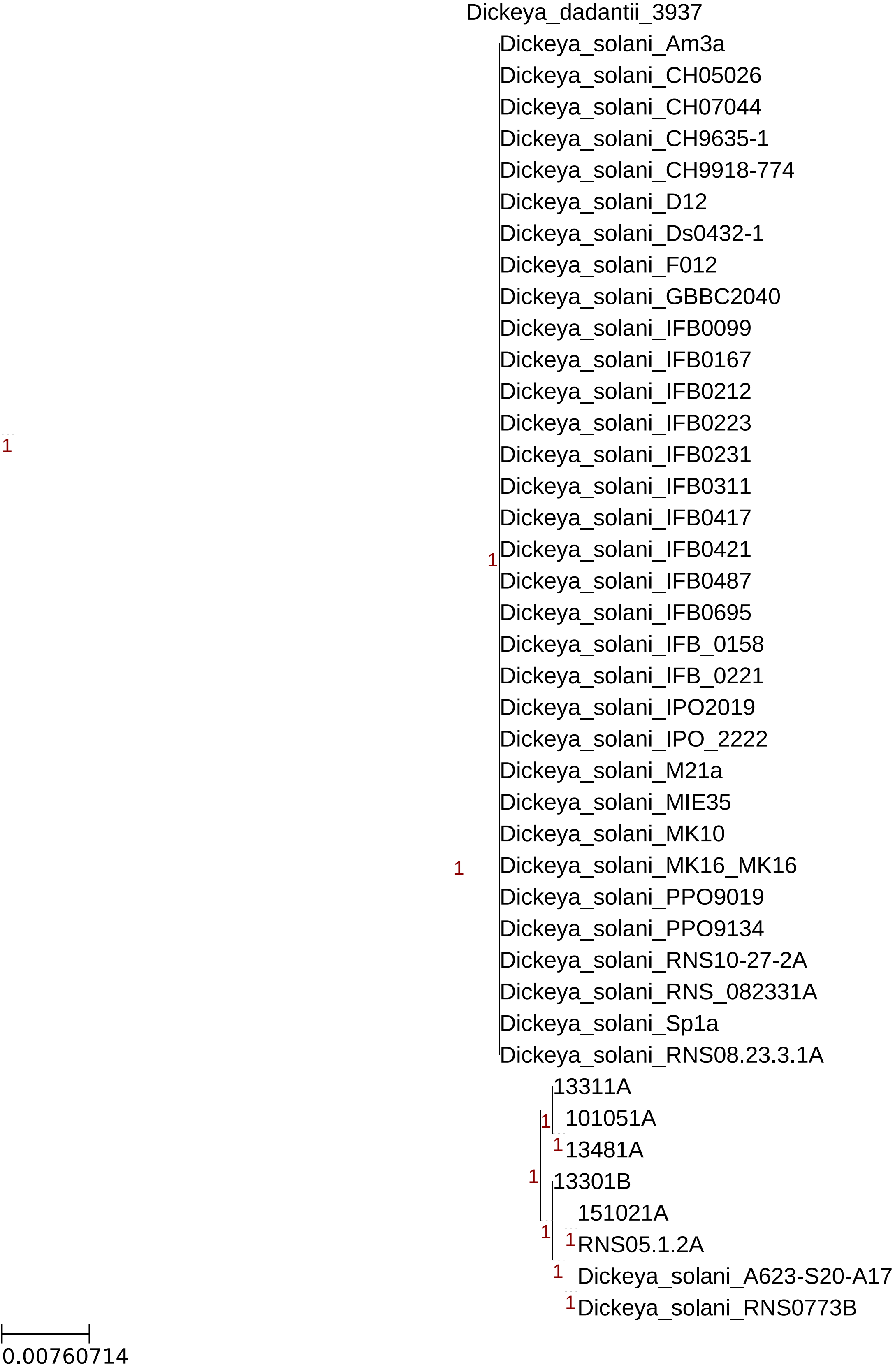

Dickeya_dadantii_3937
Dickeya_solani_Am3a
Dickeya_solani_CH05026
Dickeya_solani_CH07044
Dickeya_solani_CH9635-1
Dickeya_solani_CH9918-774
Dickeya_solani_D12
Dickeya_solani_Ds0432-1
Dickeya_solani_F012
Dickeya_solani_GBBC2040
Dickeya_solani_IFB0099
Dickeya_solani_IFB0167
Dickeya_solani_IFB0212
Dickeya_solani_IFB0223
Dickeya_solani_IFB0231
Dickeya_solani_IFB0311
Dickeya_solani_IFB0417
Dickeya_solani_IFB0421
Dickeya_solani_IFB0487
Dickeya_solani_IFB0695
Dickeya_solani_IFB_0158
Dickeya_solani_IFB_0221
Dickeya_solani_IPO2019
Dickeya_solani_IPO_2222
Dickeya_solani_M21a
Dickeya_solani_MIE35
Dickeya_solani_MK10
Dickeya_solani_MK16_MK16
Dickeya_solani_PPO9019
Dickeya_solani_PPO9134
Dickeya_solani_RNS10-27-2A
Dickeya_solani_RNS_082331A
Dickeya_solani_Sp1a
Dickeya_solani_RNS08.23.3.1A
13311A
101051A
13481A
13301B
151021A
RNS05.1.2A
Dickeya_solani_A623-S20-A17
Dickeya_solani_RNS0773B
1
1
1
1
1
1
1
1
1
1
0.00760714

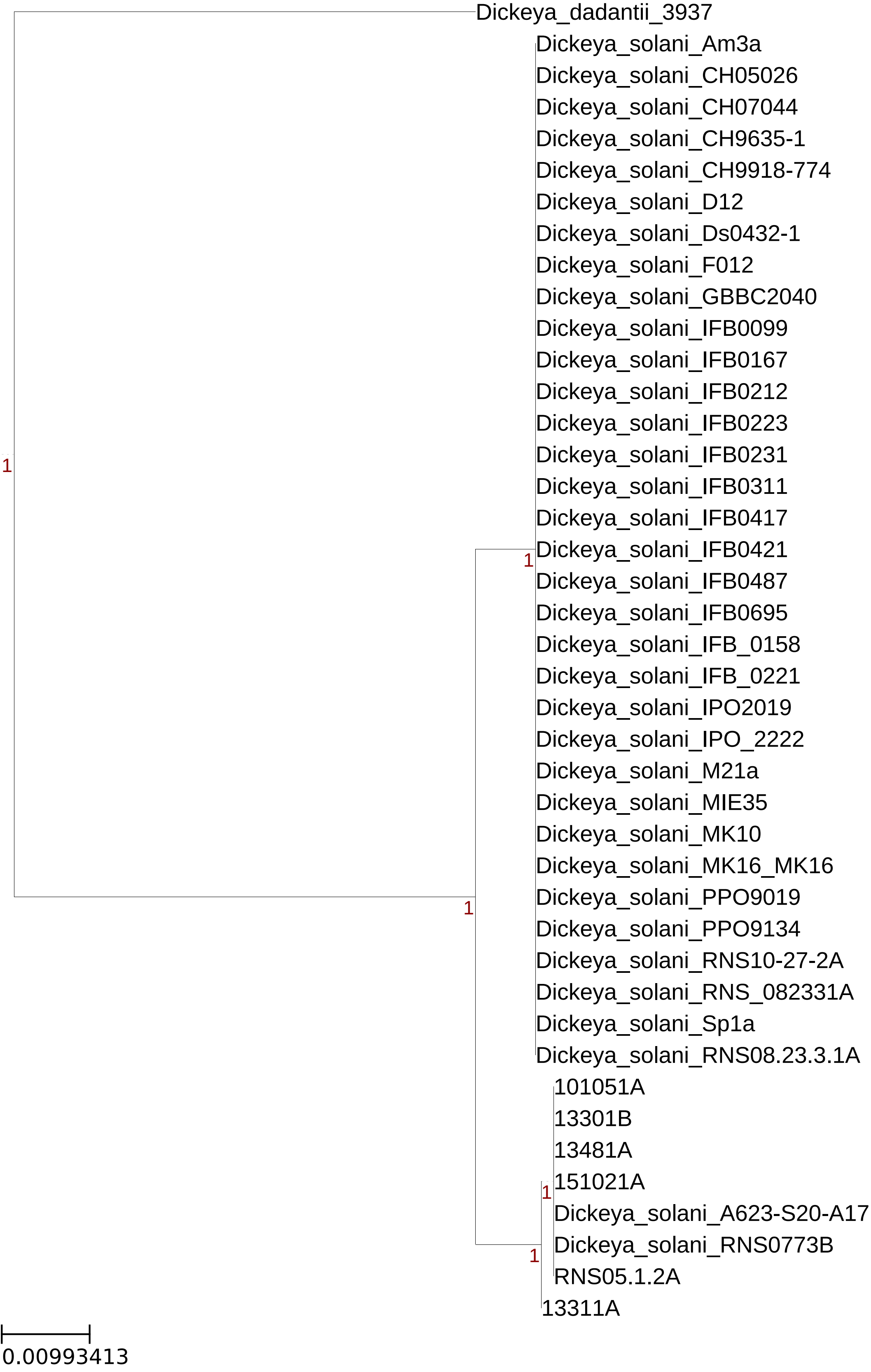
Dickeya_dadantii_3937
Dickeya_solani_Am3a
Dickeya_solani_CH05026
Dickeya_solani_CH07044
Dickeya_solani_CH9635-1
Dickeya_solani_CH9918-774
Dickeya_solani_D12
Dickeya_solani_Ds0432-1
Dickeya_solani_F012
Dickeya_solani_GBBC2040
Dickeya_solani_IFB0099
Dickeya_solani_IFB0167
Dickeya_solani_IFB0212
Dickeya_solani_IFB0223
Dickeya_solani_IFB0231
Dickeya_solani_IFB0311
Dickeya_solani_IFB0417
Dickeya_solani_IFB0421
Dickeya_solani_IFB0487
Dickeya_solani_IFB0695
Dickeya_solani_IFB_0158
Dickeya_solani_IFB_0221
Dickeya_solani_IPO2019
Dickeya_solani_IPO_2222
Dickeya_solani_M21a
Dickeya_solani_MIE35
Dickeya_solani_MK10
Dickeya_solani_MK16_MK16
Dickeya_solani_PPO9019
Dickeya_solani_PPO9134
Dickeya_solani_RNS10-27-2A
Dickeya_solani_RNS_082331A
Dickeya_solani_Sp1a
Dickeya_solani_RNS08.23.3.1A
101051A
13301B
13481A
151021A
Dickeya_solani_A623-S20-A17
Dickeya_solani_RNS0773B
RNS05.1.2A
13311A
1
1
1
1
1
0.00993413

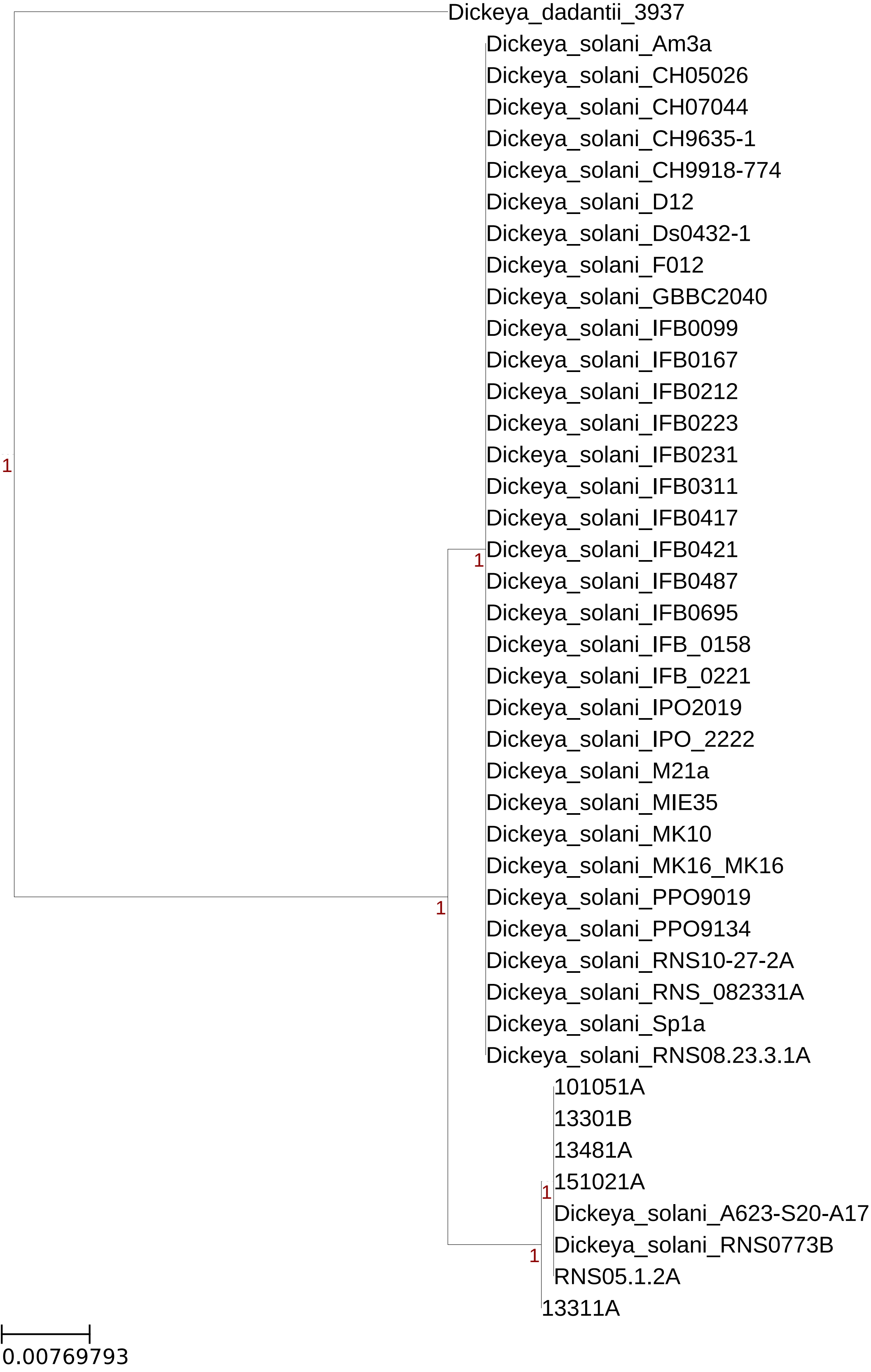
Dickeya_dadantii_3937
Dickeya_solani_Am3a
Dickeya_solani_CH05026
Dickeya_solani_CH07044
Dickeya_solani_CH9635-1
Dickeya_solani_CH9918-774
Dickeya_solani_D12
Dickeya_solani_Ds0432-1
Dickeya_solani_F012
Dickeya_solani_GBBC2040
Dickeya_solani_IFB0099
Dickeya_solani_IFB0167
Dickeya_solani_IFB0212
Dickeya_solani_IFB0223
Dickeya_solani_IFB0231
Dickeya_solani_IFB0311
Dickeya_solani_IFB0417
Dickeya_solani_IFB0421
Dickeya_solani_IFB0487
Dickeya_solani_IFB0695
Dickeya_solani_IFB_0158
Dickeya_solani_IFB_0221
Dickeya_solani_IPO2019
Dickeya_solani_IPO_2222
Dickeya_solani_M21a
Dickeya_solani_MIE35
Dickeya_solani_MK10
Dickeya_solani_MK16_MK16
Dickeya_solani_PPO9019
Dickeya_solani_PPO9134
Dickeya_solani_RNS10-27-2A
Dickeya_solani_RNS_082331A
Dickeya_solani_Sp1a
Dickeya_solani_RNS08.23.3.1A
101051A
13301B
13481A
151021A
Dickeya_solani_A623-S20-A17
Dickeya_solani_RNS0773B
RNS05.1.2A
13311A
1
1
1
1
1
0.00769793

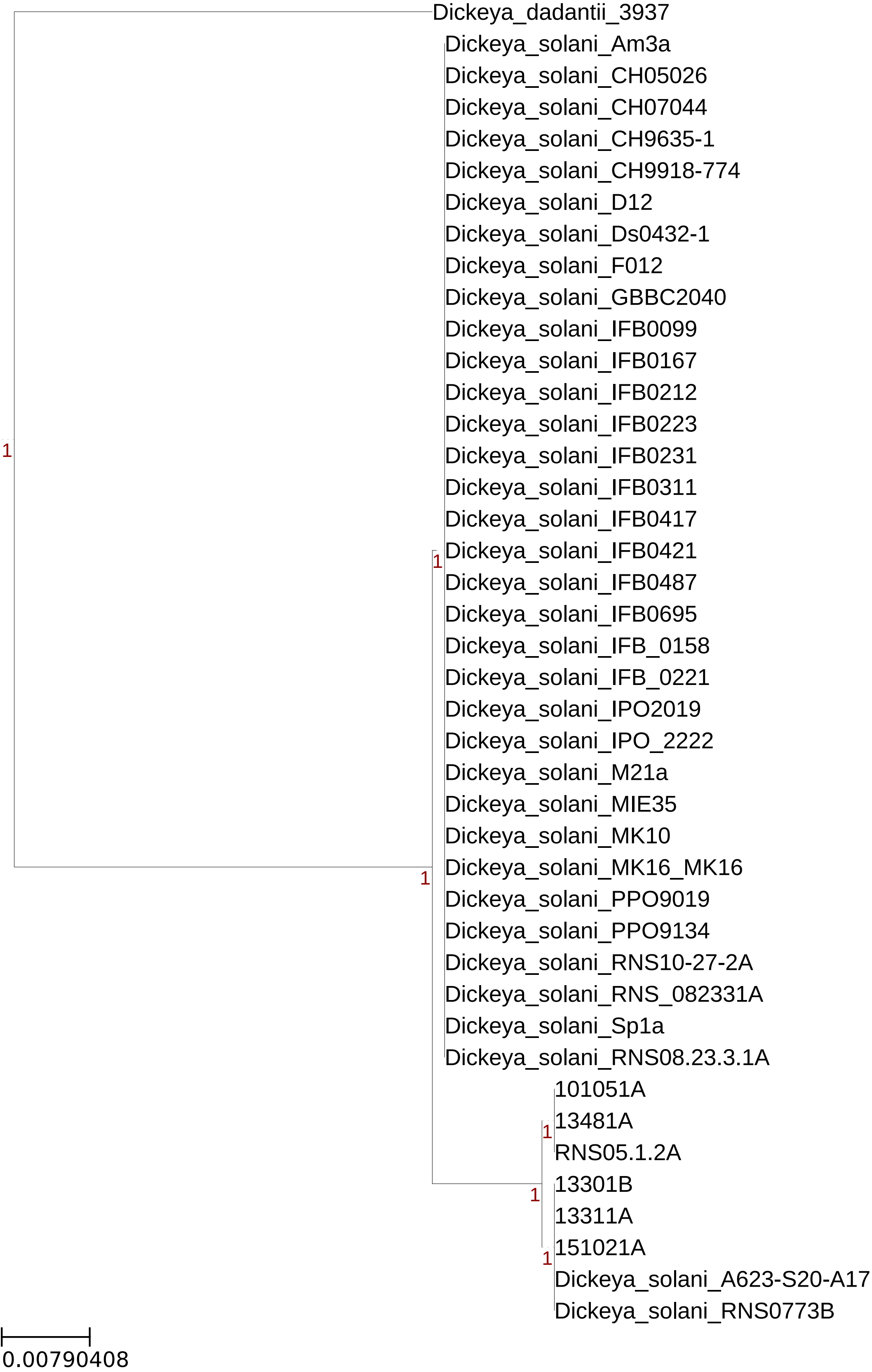
Dickeya_dadantii_3937
Dickeya_solani_Am3a
Dickeya_solani_CH05026
Dickeya_solani_CH07044
Dickeya_solani_CH9635-1
Dickeya_solani_CH9918-774
Dickeya_solani_D12
Dickeya_solani_Ds0432-1
Dickeya_solani_F012
Dickeya_solani_GBBC2040
Dickeya_solani_IFB0099
Dickeya_solani_IFB0167
Dickeya_solani_IFB0212
Dickeya_solani_IFB0223
Dickeya_solani_IFB0231
Dickeya_solani_IFB0311
Dickeya_solani_IFB0417
Dickeya_solani_IFB0421
Dickeya_solani_IFB0487
Dickeya_solani_IFB0695
Dickeya_solani_IFB_0158
Dickeya_solani_IFB_0221
Dickeya_solani_IPO2019
Dickeya_solani_IPO_2222
Dickeya_solani_M21a
Dickeya_solani_MIE35
Dickeya_solani_MK10
Dickeya_solani_MK16_MK16
Dickeya_solani_PPO9019
Dickeya_solani_PPO9134
Dickeya_solani_RNS10-27-2A
Dickeya_solani_RNS_082331A
Dickeya_solani_Sp1a
Dickeya_solani_RNS08.23.3.1A
101051A
13481A
RNS05.1.2A
13301B
13311A
151021A
Dickeya_solani_A623-S20-A17
Dickeya_solani_RNS0773B
1
1
1
1
1
1
0.00790408
